# Oral corticosteroids for post-infectious cough in adults: study protocol for a double-blind randomized placebo-controlled trial in Swiss family practices (OSPIC trial)

**DOI:** 10.1186/s13063-020-04848-4

**Published:** 2020-11-23

**Authors:** Christoph Merlo, Stefan Essig, D. Oana Brancati-Badarau, Jörg Daniel Leuppi, Benjamin Speich, Tobias E. Erlanger, Lars G. Hemkens, Andreas Zeller

**Affiliations:** 1 Institute of Primary and Community Care, Lucerne, Switzerland; 2 Centre for Primary Health Care, University of Basel, Basel, Switzerland; 3 University Clinic of Medicine, Cantonal Hospital Baselland, Liestal, Switzerland; 4 University of Basel, Basel, Switzerland; 5 Basel Institute for Clinical Epidemiology and Biostatistics, Department of Clinical Research, University Hospital Basel, and University of Basel, Basel, Switzerland; 6 Centre for Statistics in Medicine, Nuffield Department of Orthopaedics, Rheumatology and Musculoskeletal Sciences, University of Oxford, Oxford, UK; 7 Clinical Trial Unit, Department of Clinical Research, University Hospital Basel, and University of Basel, Basel, Switzerland; 8 Meta-Research Innovation Center Berlin (METRIC-B), Berlin Institute of Health, Berlin, Germany; 9 Meta-Research Innovation Center at Stanford (METRICS), Stanford University, Stanford, CA USA

**Keywords:** Corticosteroids, Post-infectious cough, Adults, Randomized controlled trial, Quality of life, Primary care

## Abstract

**Background:**

Cough is a common reason for patients to visit general practices. So-called post-infectious cough is defined as lasting 3 to 8 weeks after an upper respiratory tract infection. It can be disabling in daily activities, with substantial impact on physical and psychosocial health, leading to impaired quality of life and increased health care costs. Recommendations for the management of post-infectious cough in primary care are scarce and incoherent. A systematic review and meta-analysis of randomized clinical trials (RCT) assessing patient-relevant benefits and potential harms of available treatments identified six eligible RCTs assessing different treatment regimens (i.e. inhaled fluticasone propionate, inhaled budesonide, salbutamol plus ipratropium-bromide, montelukast, nociception-opioid-1-receptor agonist, codeine, gelatine). No RCT found clear patient-relevant benefits and most had an unclear or high risk of bias.

Post-infectious cough is thought to be mediated by inflammatory processes that are also present in exacerbations of asthma or chronic obstructive pulmonary diseases for which there is strong evidence that oral corticosteroids provide patient-relevant benefit without relevant harm. We therefore plan to conduct the first RCT evaluating the effectiveness of oral corticosteroids for post-infectious cough.

**Methods:**

We are conducting a triple-blinded randomized-controlled and multicentred superiority trial in primary health care practices in Switzerland. We will include 204 adult patients who consult their general practitioner (GP) for a cough lasting 3 to 8 weeks following an upper respiratory tract infection. Participants will be randomly allocated to either the 5-day treatment with oral corticosteroids or placebo. The *primary outcome* is cough-related quality of life assessed by the Leicester Cough Questionnaire score 14 days after randomization. *Secondary outcomes* include cough-related quality of life at several time points, overall cessation of cough and adverse events.

**Discussion:**

This RCT will provide evidence on whether oral corticosteroids are beneficial and safe in patients with post-infectious cough. Results can have a substantial impact on the well-being and management of these patients in Switzerland and beyond. An evidence-based treatment for this condition may reduce re-consultations with GPs and spending for antitussive drugs, thus possibly having an impact on health care spending.

**Trial registration:**

ClinicalTrials.gov NCT04232449. Prospectively registered on 18 January 2020.

## Administrative information

The order of the items has been modified to group similar items (see http://www.equator-network.org/reporting-guidelines/spirit-2013-statement-defining-standard-protocol-items-for-clinical-trials/).

**Table:** 

Title {1}	Oral corticosteroids for post-infectious cough in adults: study protocol for a double-blind randomized placebo-controlled trial in Swiss family practices (OSPIC trial)
Trial registration {2a and 2b}.	NCT04232449, ClinicalTrials.gov All items from the World Health Organization Trial Registration Data Set are provided in the supplemental material (Supplement 1).
Protocol version {3}	Version 2.1/ 29.01.2020
Funding {4}	Financial support: SNSF (Swiss National Science Foundation), Investigator Initiated Clinical Trials grant (IICT 2018 call, 33IC30_179657 3). Material and infrastructure support: Centre for Primary Health Care, University of Basel, Basel, Switzerland.
Author details {5a}	Dr. Christoph Merlo, Head of Institute of Primary and Community Care, Lucerne, Switzerland. Stefan Essig, MD, PhD, Institute of Primary and Community Care, Lucerne, Switzerland. D Oana Brancati-Badarau, PhD, Centre for Primary Health Care, University of Basel, Basel, Switzerland. Prof. Dr. med. Jörg Daniel Leuppi, University Clinic of Medicine, Cantonal Hospital Baselland, Liestal; and University of Basel, Basel, Switzerland. Benjamin Speich, PhD, Basel Institute for Clinical Epidemiology and Biostatistics, Department of Clinical Research, University Hospital Basel, University of Basel, Basel, Switzerland; and Centre for Statistics in Medicine, Nuffield Department of Orthopaedics, Rheumatology and Musculoskeletal Sciences, University of Oxford, Oxford, United Kingdom. Tobias E Erlanger, PhD, Clinical Trial Unit, Department of Clinical Research, University Hospital Basel, University of Basel, Basel, Switzerland. Lars G Hemkens, MD, MPH, Basel Institute for Clinical Epidemiology and Biostatistics, Department of Clinical Research, University Hospital Basel; and University of Basel, Basel, Switzerland. Prof. Dr. med. Andreas Zeller, MSc, Head of the Centre for Primary Health Care, University of Basel, Basel, Switzerland.
Name and contact information for the trial sponsor {5b}	Prof. Dr. med. Andreas Zeller Head of the Centre for Primary Health Care (uniham-bb) University of Basel Cantonal Hospital Baselland Rheinstrasse 26 CH-4410 Liestal Tel: +41 (0)61 925 20 75 Email: andreas.zeller@unibas.ch
Role of sponsor and funder {5c}	This is an Investigator-Initiated Trial (IIT) and Professor Dr. med. Andreas Zeller is acting as Sponsor-Investigator. Prof. Zeller led the study design, is responsible for project development and implementation, for overseeing collection, management, analysis and interpretation of data. Prof. Zeller will contribute to the writing of the report and will have ultimate authority over the decision to submit the report for publication. This study is funded by the Swiss National Science Foundation. The funder had no role in designing the study and will also have no role in conducting the study or analysing and reporting study results.

## Introduction

### Background and rationale {6a}

Cough as a symptom of respiratory infections is frequent in primary care and is one of the most common causes to seek medical advice in general practices (GP) [1]. Cough after an upper respiratory tract infection can be very bothersome and disabling in daily activities and has a significant impact on physical and psycho-social health, leading to impairment in quality of life (QoL) [2]. Post-infectious cough, also known as subacute cough, is defined as lasting between 3 and 8 weeks following an upper respiratory tract infection [3]. It results from a protracted inflammation of the bronchial mucosa after a viral infection, an epithelial damage with irritant-receptors laid open and/or a temporary bronchial hyper-responsiveness [3, 4]. The diagnosis is based on the patient’s clinical history, physical examination and exclusion of other causes such as chronic obstructive pulmonary disease (COPD) or asthma [5, 6].

Recommendations for the management of post-infectious cough in general practice are scarce and inconsistent [3, 4]. A previous systematic review and meta-analysis of randomized controlled trials (RCT) carried by our group provided a wide overview of treatment options for primary care patients with post-infectious cough and examined the patient-relevant benefits and potential harms of available therapies [7]. The review found only six RCTs assessing diverse treatment regimens, such as inhaled fluticasone propionate, inhaled budesonide, salbutamol plus ipratropium-bromide, montelukast, nociception-opioid-1-receptor agonist, codeine and gelatine. Most of the studies included in the review had an unclear or high risk of bias [7]. None of the individual RCTs found clear patient-relevant benefits for patients with post-infectious cough lasting 3 to 8 weeks. Two RCTs assessed inhaled corticosteroids for post-infectious cough [8, 9]. Pornsuriyasak et al. [9] included a total of 30 patients and found no benefit of inhaled steroids on cough outcomes at all. The trial by Ponsioen et al. [8], which included 135 patients with cough lasting for 2 weeks or more, indicated a potential benefit of inhaled steroids on cough in the overall study population that was explained by beneficial effects in the non-smoker sub-group. However, the study included a relevant number of patients (*n* = 44; 33%) without post-infectious cough (lasting less than 3 weeks) and did not report results for this group separately [8].

Clinical guidelines and recommendations on the use of inhaled corticosteroids are unclear [3, 4, 10]. A Cochrane review published in 2013 evaluated studies in which inhaled corticosteroids were tested in individuals with post-infectious or chronic cough [11]. A majority of the studies focused on patients with chronic cough and only two examined the benefits for post-infectious cough [11]. The authors concluded that no recommendation can be proposed due to the high heterogeneity and inconsistency of the studies and their results [11]. Additionally, an RCT in family practices in England found no benefit in terms of duration or severity of cough after a 5-day treatment with oral corticosteroids compared to placebo for adult patients with acute lower respiratory tract infection and without asthma [12]. Another RCT assessed the effectiveness of oral corticosteroids for patients with acute sore throat, 55.9% of which also reported a cough in the course of the illness. In this study, patients who received a single oral dose of 10 mg of dexamethasone were not more likely at 24 h to experience complete resolution of symptoms compared to patients on placebo [13].

Many of the symptoms in post-infectious cough are thought to be mediated by inflammatory processes that are also present in exacerbations of asthma or COPD [5, 6]. For these conditions, there is strong evidence that short-term oral corticosteroids provide patient-relevant benefits [14] and prednisone (tablets at a dose of 40 mg once daily for 5 to 7 days) is a well-established oral steroid for acute asthma or exacerbation of COPD [5, 6]. However, at present, there is no established evidence-based treatment option for post-infectious cough, despite it being a very frequent condition. There is also considerable uncertainty regarding patient benefits from using inhaled or oral corticosteroids. The systematic search of our group did not identify any published RCT that assessed short-term use of oral corticosteroids for post-infectious cough [7] (we updated our search in October 2018 and still found no pertinent trial). We screened multiple study registries using the International Clinical Trials Registry Platform from the World Health Organization (last search June 2020) and again found no trial investigating the use of oral corticosteroids for post-infectious cough. One registered trial aimed to assess the efficacy of inhaled budesonide in adult patients with chronic cough (registration ID NCT02715167) and several other studies planned to assess the efficacy of corticosteroids in children with acute or chronic cough (registration ID ACTRN12616001713482; ChiCTR-TRC-13003182; ACTRN12611000589987). A well-conducted randomized placebo-controlled trial is needed to determine the benefits and harms of using oral corticosteroids to treat post-infectious cough in patients in primary care.

## Objectives {7}

We will investigate whether a 5-day treatment with 40 mg (2 tablets of 20 mg) orally administered prednisone provides patient-relevant benefits for adults with post-infectious cough triggered by an upper respiratory tract infection and seeking care in adult primary care practices. We hypothesize that the prednisone treatment will be superior to placebo and improve patients’ cough-related QoL at 14 days from group allocation. This randomized placebo-controlled trial aims to assess whether the benefits and harms of a 5-day prednisone treatment differ from those of a 5-day course of placebo.

## Trial design {8}

We designed a protocol for a 1:1 randomized, parallel-group, placebo-controlled, triple-blinded, multicentred superiority trial in a primary health care setting, with blinded patients, physicians and outcome assessors. Recruitment will take place in general practices in Switzerland and participants in both the prednisone and the control groups will be followed-up at different time points: first at day 7, then day 14 and day 28 and at 3 months from the time of randomization. This protocol follows the Standard Protocol Items: Recommendations for Interventional Trials, 2013 statement [15].

## Methods: Participants, interventions and outcomes

### Study setting {9}

Patients with post-infectious cough will be recruited by participating doctors in primary practices from cantons in the German-speaking part of Switzerland. Patient recruitment will continue until the sample size is reached. A list of the general practices currently taking part in the study can be obtained from the Sponsor-Investigator. Study participants will be followed-up through phone calls carried by study research staff at the Clinical Trial Unit (CTU) at the University Hospital Basel.

### Eligibility criteria {10}

In order to be eligible for the study, patients will have to fulfil all the inclusion criteria:

1. Age ≥ 18 years
2. Seeing a GP for a dry or productive post-infectious cough (3 to 8 weeks) after an upper respiratory tract infection
3. Able and willing to give informed consent by themselves.

The presence of any one of the following exclusion criteria will lead to patient’s exclusion from the study:

1. Hypersensitivity to prednisone or to one of the adjuvants in the drug’s composition
2. Known or suspected diagnoses associated with cough, such as pneumonia, allergic rhinitis, sinusitis, bronchial asthma, COPD, gastroesophageal reflux disease
3. Other chronic diseases such as bronchiectasis, cystic fibrosis, cancer, tuberculosis, heart failure
4. Use of inhaled or oral corticosteroids within the last 4 weeks
5. Immunodeficiency/immunocompromised state (e.g. cancer chemotherapy, HIV infection)
6. Pregnancy/breastfeeding
7. Regular treatment known to be associated with cough (e.g. angiotensin-converting enzyme inhibitors)
8. A documented diagnosis of glaucoma or osteoporosis in the GP’s patient health record
9. History of fractures due to osteoporosis
10. Uncontrolled diabetes mellitus (as deemed by GPs who appraise whether the potential side effects of short-time corticosteroids on glucose levels exceed the hypothesized benefit on cough).

### Who will take informed consent? {26a}

The OSPIC study will be advertised through posters and information leaflets displayed in the collaborating GP practices. Patients with post-infectious cough presenting to their GP will be told about the OSPIC trial and provided with a study leaflet, participant information sheet and a consent form by their GP. They will be invited by the GP to take part after being given full written and verbal explanations of the trial purpose, potential benefits and risks and the procedures involved. Those who agree to join the study will be asked to provide written consent and will be screened against the full eligibility criteria described above. Participants will have sufficient time to ask questions and GPs will make sure to underscore that participation is voluntary and that declining to join the study does not influence in any way the standard of care provided to patients.

### Additional consent provisions for collection and use of participant data and biological specimens {26b}

During the informed consent process with the GP, participants will be asked to give written permission for the storage and future use of the data resulted from the study. The health-related data will be stored in an anonymized way by using the participant’s code and can be analysed for the purposes of future research projects. No biological specimens will be collected for the purpose of the OPSIC study.

## Interventions

### Explanation for the choice of comparators {6b}

Patients in the control group will receive 10 placebo pills of 20 mg (40 mg of placebo). Placebo pills are described in detail in the next section. Placebo will be used as a comparator in this study to prevent various biases (in particular as the primary endpoint is patient-reported). Potential implications on a limited applicability of the results are acknowledged and will be discussed in the study results publication. From an ethical point of view, an inactive control (placebo) seems justified since there is no established therapy for post-infectious cough and because the symptoms resolve over time due to the natural course of the disease [7, 12].

### Intervention description {11a}

The expected duration of participation in the study is around 3 months. This includes the day 0 (randomization) study activities, the treatment period of 5 days and four follow-up phone calls. At the baseline visit (day 0), patients will receive pre-randomized identically looking, individually labelled medication glass jars with daily doses of 40 mg (2 tablets of 20 mg per dose) of visually identical prednisone or placebo pills. Patients in the intervention group will receive 10 white tablets of PREDNISON Galepharm Tabl 20 mg and be asked to take 2 pills orally once a day during breakfast for 5 days. Patients in the control arm will also take 2 placebo tablets once daily for 5 days. The placebo tablets match in appearance, diameter and height the intervention medication. These are manufactured by Apotheke Hotz (Küsnacht, Zürich, Switzerland) and contain 140 mg Lactose monohydrate, 68 mg microcristalline cellulose, 5 mg Croscarmellose sodium and 2 mg Magnesium stearate. Verbal and written instructions on how the drugs should be taken will be provided to the study participants.

Pharmacokinetic evidence suggests that a minimum dose of 20 mg prednisone daily is required for non-asthmatic patients to achieve an adequate anti-inflammatory effect [16]. We select a dose of 40 mg (2 tablets of 20 mg) of prednisone which is well established as treatment in patients with acute asthma or exacerbation of a chronic obstructive lung disease [14]. A dose of 40 mg of prednisone will ensure sufficient pharmacokinetic activity to be able to reveal a potential treatment effect in post-infectious cough. We select a treatment duration of 5 days since post-infectious cough is thought to be mediated by inflammatory processes comparable to those in exacerbations of asthma or COPD. For these conditions, there is strong evidence that short-term oral corticosteroids for 5 days provide patient-relevant benefit without relevant harm [14].

### Criteria for discontinuing or modifying allocated interventions {11b}

Due to the short intervention of 5 days, no treatment modifications are planned unless in the (unlikely) occasion of any side effects. Even though the likelihood is very low, adverse events (AE), such as allergic reactions to the study drug, psychotic or pre-psychotic episode, or serious adverse events (SAE), sepsis, venous thromboembolism, fracture, can occur [17]. In any of these cases, the treatment will be stopped immediately. Medication will also be discontinued for other urgent reasons, such as pregnancy, a cancer diagnosis or an infection other than an upper respiratory tract infection.

### Strategies to improve adherence to interventions {11c}

In order to facilitate adherence to the study intake schedule, participants are given a written medication guide. GPs will inform patients in depth on the importance to adhere to the 5-day medication for ensuring the effectiveness of treatment. They will emphasize the value to the trial conduct of participants’ availability for the follow-up phone calls. Furthermore, the dosing schedule is very convenient as the drugs need to be taken only once a day during breakfast and for a clearly defined and limited timeframe. In the event of a missed dose, patients are instructed to continue to take the medication the next day. Adherence to the study procedures will be checked at the follow-up phone call on day 7 from randomization when research staff will ask participants about their medication intake. In case the study medication is prematurely stopped or discontinued patients are asked to return the empty drug glass jars to their GP. All these measures and participants’ specific details will be documented in the Case Report Form (CRF).

### Relevant concomitant care permitted or prohibited during the trial {11d}

Apart from the use of corticosteroids, any co-treatment or co-medication (i.e. antitussives, inhalation, herbal teas, and homoeopathic pharmaceuticals) is permitted. Any other medical intervention used by study participants will be recorded in the electronic Case Report Forms (eCRF) to analyse the potential influence on outcomes. Throughout the trial, participants’ medication can be re-evaluated by their GPs based on clinical needs. Cases may arise when the patients’ clinical condition is worsening or the patient presents to the GP for an additional consultation before the 5-day treatment is over. Treating doctors can independently decide to change to open-label treatment, adjust medication if they deem it necessary and for the benefit of their patients or choose additional therapeutic options. All participants will be asked at follow-up about concurrent medication, including if they started a treatment with antibiotics.

### Provisions for post-trial care {30}

Corticosteroid potential side effects and complications will be systematically recorded from the time of randomization until the last follow-up call at 3 months. In case of persistent coughing at 3 months from randomization, the patient will be advised to visit the GP again for a new assessment and necessary further investigations. GPs and research staff are instructed to document time of onset, duration, resolution, actions to be taken, assessment of intensity and relationship with study treatment. An insurance covering the study activities is contracted through the Sponsor’s institution, the University of Basel.

Participants will be advised that they need to use contraceptives for the duration of the treatment and that they should inform the GP or the study team in case they suspect they have become pregnant. Women with anamnestic risk of a pregnancy (unprotected sexual intercourse in the last 2 weeks) shall be excluded from this study. If a participant will become pregnant during follow-up, the participant will visit her gynaecologist. The GP will document the course and the outcome of the pregnancy.

### Outcomes {12}

The primary outcome is the cough-related QoL at 14 days after randomization. We will use the validated Leicester Cough Questionnaire (LCQ) score [18–21] to assess the impact of the study medication on patients’ QoL (mean difference between arms measured 14 days after randomization). Total and individual LCQ domain scores will be calculated. The LCQ is also suitable for capturing longitudinal developments in cough and cough-related well-being and can be useful in clinical trials assessing new medications for cough [20].

#### Secondary outcomes

On days 7, 14 and 28 and at 3 months, patients will be called by trained and experienced research staff and asked to complete the LCQ on the phone. Appointments for the next phone calls will be set during the previous phone call and will assess:

- Cough-related QoL assessed by the LCQ score at 7 and 28 days and 3 months after randomization (i.e. mean difference between arms measured 7 and 28 days and 3 months after randomization)
- Cough-related QoL sub-domains physical, psychological, and social at 7, 14 and 28 days and 3 months after randomization (i.e. mean difference between arms measured 7, 14 and 28 days and 3 months after randomization)
- Overall cessation of cough at 7, 14 and 28 days and 3 months after randomization (binary variable yes/no; comparison of proportions)
- Incidence rate of re-consultations at GP and/or hospitalisations for potential illness deterioration and the occurrence of side effects within 3 months following randomization (comparison of proportions between treatment arms)

The following safety outcomes will be captured:

- Incidence rate of re-consultations at GP and/or hospitalisations within 3 months following randomization
- Total AE within 3 months after randomization
- SAE within 3 months after randomization
- Changes in glucose levels for patients with pre-study controlled diabetes that are deemed by GP to exceed the hypothesized benefit on cough

Incidence rates of AE and SAE will be assessed according to the World Health Organization-Uppsala Monitoring Centre (WHO-UMC) [22] causality categories “certain”, “probable”, “possible” and “unlikely” during 3 months following randomization (further details in the “Adverse event reporting and harms {22}” section). Continuous outcomes will be assessed by comparing mean values. Medians will be considered in addition if we identify severe departures from normal distribution.

### Participant timeline {13}

General practitioners will enrol approximately 5 but not more than 10 participants from patients presenting for a consultation due to post-infectious cough (baseline visit) over a period of 18 months until the sample size is reached (*N* = 204). Eligible patients who consent to the study will be randomly assigned (1:1) by their GP to the active treatment or the control group. At baseline, the GP will decide what diagnostics are necessary and will complete an individual CRF with the participant’s baseline socio-demographic information. If performed, the GP will also record diagnostic test results. Participants will be asked to complete the standardized LCQ questionnaire and hand it to the GP on day 0. Participants will also be informed about the follow-up calls and that the next telephone appointment will be at day 7 of the trial. After inclusion in the study, it is at the discretion of the treating GP to re-assess each participant at the general practice, when and as often as clinically needed. Physical examinations, lab testing, performing X-rays (e.g. chest) or decisions to hospitalize patients, if indicated, can be carried by the GP.

**Fig. 1:**
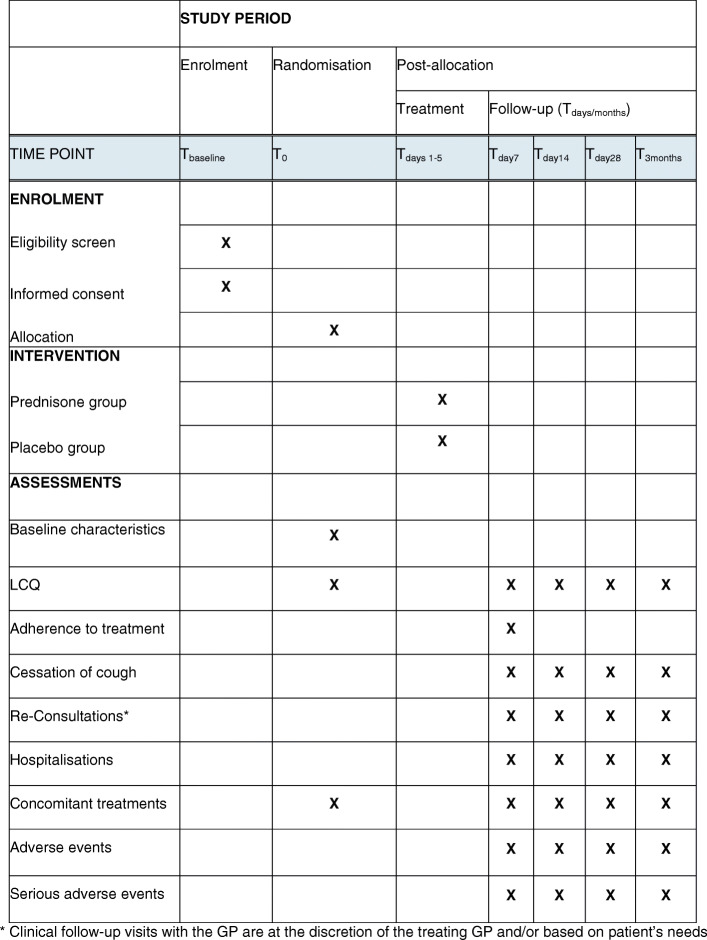
Study schedule.*Clinical follow-up visits with the GP are at the discretion of the treating GP and/or based on the patient’s needs

Follow-up calls lasting around 15 min each are carried by research staff at the CTU, University Hospital Basel on days 7, 14 and 28 and at 3 months after randomization. In case participants are not reached at the first call, follow-up phone calls will be performed several times and participants will be sent reminders by email. If participants are not reached for the follow-up calls at day 7, day 14 or day 28, then a call will be made in the next 2 days (day 7 + 2, day 14 + 2; day 28 + 2). Participants will be called during the next 7 days (3 months + 7 days) when research staff is unable to reach them at 3 months. At each follow-up, participants will be asked to complete the LCQ and answer other questions regarding cough status, side effects, concomitant therapy, cessation of cough, re-consultations/hospitalization, AE and SAE. (Fig. 1).

### Sample size {14}

Sample size was estimated to have 80% power to detect the minimal clinically important difference (MCID) set at 1.3 points LCQ [23]. To be able to detect an MCID of 1.3 points with a power of 80%, a total of 204 patients need to be recruited for both arms. This was calculated without considering intra-patient correlation (IPC) correlation rho (ρ) between baseline and follow-up and without potential intra-cluster correlation (ICC) of patients treated by the same physician. To further increase power, we ignored ρ by calculating the sample size for a two-sample *t* test with a two-sided alpha threshold of 5%. Due to the fact that the number of recruited patients per GP is limited to 10, ICC might remain small. We expect a drop-out rate of 10%, similar to that in the trial by Wang et al. [24]. Sample size estimation was based on the assumption that individual LCQ scores are normally distributed. Raj et al. [23] reported a standard deviation (SD) of 3.3 points. A recent trial with a design and study population similar to ours reported a SD of 2.9 [24]. We decided to use the more conservative assumption of 3.3 points. A less conservative choice of an SD of 2.9 and a ρ of 0.4, with the other parameters remaining the same, would have required a sample size of 120. Hence, we assume that by the conservative choice of SD and by neglecting ρ, our calculation will sufficiently compensate for the loss of power due to ICC and drop-outs. To compute the *t* test, the current version of the *R* language and environment (R Foundation, www.r-project.org) function “power.t.test” of the “stats” package was applied.

### Recruitment {15}

A total of 204 eligible patients with post-infectious cough will be recruited from general practices in North-western and Central Switzerland within a period of approximately 18 months. The geographical area is large enough to recruit the required number of patients in the indicated timeframe as nearly 40% of adults can be affected by post-infectious cough after an acute respiratory infection [24, 25]. It is envisaged that the recruitment period will last 18 months: First-patient-in in autumn 2020 and last-patient-out in spring 2022. In case of recruitment difficulties due to scheduled numbers of participants not being reached at predefined milestones, the limit of maximum of 10 randomized patients per GP can be increased. To include 204 participants, the recruitment period will cover two winter seasons when the incidence of upper respiratory tract infection is very high and post-infectious cough is very common. Even though coughing is prevalent throughout the year and patients can be affected in summer as well, we expect that most participants will be enrolled during the cold months. In case of unforeseen difficulties leading to lower number of participants than reasonably expected after 9 months (less than 1/3 of the target study population is enrolled), the recruitment areas can be enlarged as the investigators have established cooperation with institutes of general medicine in the Eastern and the French-speaking parts of Switzerland. If the enrolment goals are not met, the study will be submitted to other regional ethics committees in order to geographically expand the recruitment area.

#### Role of COVID-19

In January 2020, the respiratory disease outbreak caused by the novel coronavirus SARS-CoV-2 was declared a Public Health Emergency of International Concern by the World Health Organization [26, 27]. In the early stage of an infection with SARS-CoV-2, the most prevalent symptoms are fever and acute cough [28]. Therefore, patients with a SARS-CoV-2 infection have an acute cough (< 3 weeks duration) [29] and would not fulfil the inclusion criteria (cough lasting 3 to 8 weeks) for this trial. Further, patients with suspected SARS-CoV-2 infection in Switzerland are strongly recommended by the Federal Department of Public Health to directly present to specialized test centres and to avoid visiting their GPs [30]. Thus, it is unlikely that patients with COVID-19 would be suitable for inclusion in this study.

## Assignment of interventions: allocation

### Sequence generation {16a}

The randomization procedure will be implemented by the Clinical Trial Unit of the University Hospital Basel, which will generate a randomization list with a 1:1 treatment allocation. This list will be the basis for the University Hospital Basel Pharmacy to perform block randomization per practice, to label and to pack the study medication in glass jars. All GP practices will receive pre-randomized identically looking medication packages which will be handed to participants in the order of reception. Following this procedure, participants will therefore be randomly allocated to either prednisone or placebo.

### Concealment mechanism {16b}

With the randomization list being prepared by the CTU Basel and only accessible to the University Hospital Basel Pharmacy for preparing the sequentially numbered medication packages, the treatment allocation is concealed from patients, physicians, outcome assessors and other involved personal.

### Implementation {16c}

Participants will be enrolled by the GPs and will be assigned to the intervention randomly. GPs will distribute the randomized medication in the pre-established order set by the University Pharmacy Basel. For each medication package dispensed, the GP will record at the time of randomization the individual participants’ code, the allocated medication label and the dispensation date in a drug accountability log. Participants are required to return unused investigational treatments to their GPs.

## Assignment of interventions: blinding

### Who will be blinded {17a}

All GPs, clinical investigators, outcome assessors and research staff involved in the study, as well as all patients, will remain blinded with respect to the randomization throughout the trial. Participants in the study will receive identically looking medication jars with an accompanying guide stating that they are taking either placebo or prednisone tablets for a period of 5 days as part of the OSPIC study.

### Procedure for unblinding if needed {17b}

In case participants require hospitalizations or they consult a different doctor (not their GP), they are encouraged to take the medication guide with them. GPs will not have access to the randomization list and in case of urgency, they have to make an unblinding request with the OSPIC study team. In a next step, the OSPIC team (as soon as possible and during working hours) will request the unblinding from the CTU which will then break the code by using the study database secuTrial®. Each unblinding will be documented in the database’s integrated audit trail system.

## Data collection and management

### Plans for assessment and collection of outcomes {18a}

Data acquisition will be performed at the baseline visit by GPs and will be captured with a study developed questionnaire (CRF) and the LCQ. The GP will record individual socio-demographic characteristics and medical history, including age, sex, smoking behaviour, information on household smoking, symptoms, current treatment and doctor consultations. If performed, the GP will also record diagnostic test results such as CRP test, white blood cell count, body temperature, blood pressure, pulse, oxygen saturation, previous or present X-Ray, previous or present lung function assessments. The LCQ is a validated QoL measurement tool for non-specific cough, developed for self-administration and takes 5 to 10 min to complete [18, 19]. It has a total score (addition of domain scores) that ranges from 3 to 21 points, with a higher score corresponding to better health status [19–21]. The LCQ has already been used in a similar randomized-controlled trial assessing the effectiveness of montelukast in the treatment of post-infectious cough [24]. It is short, easy to administer and assesses the impact of cough on various aspects of life, including emotions, sleeping behaviour, work and relationships. The LCQ contains 19 items divided over 3 domains: physical (8 items), psychological (7 items) and social (4 items); with a 7-point Likert scale [19, 20].

At baseline, the GP will hand participants the LCQ and will be available to answer questions. Participants will complete the LCQ on paper at baseline and over the telephone at follow-up. The LCQ questions will be asked by trained research staff from the CTU Basel at follow-up and recorded electronically. We will use the validated German version of the original LCQ [18]. Data collection forms can be obtained from the OSPIC trial Sponsor-Investigator.

### Plans to promote participant retention and complete follow-up {18b}

Participants are encouraged by the GP to answer the follow-up questions posed by the research staff of the CTU Basel. If patients prematurely stop the study or do not answer the follow-up call, the study team can contact the GP to ask about possible GP visits, AE or SAE or hospitalizations (i.e. for pneumonia). Data will be collected until the time of withdrawal and will be analysed in the intention to treat analysis.

### Data management {19}

All study data will be stored at the CTU, including the paper questionnaire completed by GP, which will be imported into an eCRF by trained study nurses and captured via a secuTrial© database based at the University Hospital Basel. In case the paper CRF raises queries, these will be resolved through inquiries with individual GP. Read-out CRF data will be formatted and merged with phone interview data into the eCRF. Direct access to source documents will be permitted for purposes of monitoring, audits and inspections. Study data entered in the eCRF are only accessible to authorized persons and an integrated audit trail will maintain a record of initial entries and any changes made, time and date of entry and user name of the person authorizing the entry or change. The eCRF will be implemented by the data management group at the CTU of the University Hospital Basel.

### Confidentiality {27}

Confidentiality will be guaranteed during the study by the Sponsor-Investigator who will ensure the study’s compliance with national and international data security. All study data will be coded by the GP, stored and analysed in a coded manner. Password protection and user right management is used for the eCRF and ensures that only authorized study personal, data managers and local authorities, when permissible by law and necessary, will have access to the data during and after the study. Participant contact information will be collected for carrying follow-up calls and will be filled in the paper CRF form by the GP. Only research staff conducting the follow-up interviews will have access to the participants’ contact data. Participant lists will be kept at the GP practices for the entire duration of the study. After the end of the study, the lists will be sent to the Sponsor-Investigator and included in the Investigator Site File (ISF). The ISF will be archived for 10 years according to International Conference on Harmonisation – Good Clinical Practice (ICH-GCP) [31]. The study team at the CTU will maintain a separate participant/contact list, which will be included into the ISF at the end of the study. All involved parties must keep the participant data strictly confidential.

### Plans for collection, laboratory evaluation and storage of biological specimens for genetic or molecular analysis in this trial/future use {33}

Not applicable, no biological specimens are collected for the purposes of the OSPIC study.

## Statistical methods

### Statistical methods for primary and secondary outcomes {20a}

Detailed description of analyses will be defined in a statistical analysis plan (SAP) before unblinding the trial. The SAP will consider the ICH E9 Guideline Statistical Principles for Clinical Trials [32]. Changes to the SAP will be justified and reported under version control at the CTU, Basel. Analysis of the primary objective will follow the intention-to-treat (ITT) principle. It will be based on the full analysis set (FAS) which will include all patients who were randomized and gave informed consent. Patient data will be analysed according to their treatment allocation.

We will test if there is a difference in the LCQ score between the intervention and control group 14 days after randomization. A treatment effect of 1.3 points increase will be considered as MCID. The hypothesis will be tested using analysis of covariance (ANCOVA). We will report the treatment effect with 95% confidence intervals (CI). Covariates of the ANCOVA model will be baseline LCQ scores, duration of cough, age, sex and smoking status. Patients are considered being smokers when answering that they smoked “more than 100 cigarettes in their life”, they smoke “daily” or “sometimes” [33]. Baseline characteristics of patients in the FAS will be presented stratified by group and summarized in a table.

To assess the robustness of our primary analysis, an analysis of the primary outcome without imputing data (complete case analysis) will be performed. In order to estimate the effect of fully adhering to the study protocol, an analysis of the primary outcome using the per-protocol data set (PPS, including all patients with full (i) adherence to the allocated 5-day treatments (took all doses as defined in the study protocol) and (ii) complete primary outcome and LCQ [18, 20] score at baseline) will be conducted. We will also explore interactions between covariates of the ANCOVA model of the primary analysis and how the effect of the intervention varies among GP practices. For this, a linear mixed-model with treatment group as a fixed-effect and GP practices as a random effect will be fit.

Summary statistics of cough-related QoL assessed by the LCQ score at 7 and 28 days and 3 months after randomization, cough-related QoL sub-domains physical and psychological, and social at 7, 14 and 28 days and 3 months after randomization will be presented in tables and figures. Total number and percentages will be calculated for overall cessation of cough at 7, 14 and 28 days and 3 months after randomization, incidence rate of re-consultations at GP and/or hospitalizations within 3 months. Total number and percentages will be calculated for incidence rate of re-consultations at GP and/or hospitalizations, and total AE and SAE stratified by WHO-UMC causality categories [22] within 3 months after randomization. Statistical analysis will be performed by the CTU of the University Hospital Basel using R language and environment (R Foundation, www.r-project.org).

### Interim analyses {21b}

Not applicable, no interim analyses are planned.

### Methods for additional analyses (e.g. subgroup analyses) {20b}

We will conduct one subgroup analysis, comparing effects on the primary outcome in current smokers vs. current non-smokers. Subgroup effects will be analysed by interaction tests and interpreted fully exploratory. We expect that effects are more pronounced in non-smokers according to reports by Ponsioen et al. [8].

### Methods in analysis to handle protocol non-adherence and any statistical methods to handle missing data {20c}

Protocol non-adherence and impact of missing data will be assessed using a dataset that includes all patients with full (i) adherence to the allocated 5-day treatments (took all doses as defined by the study protocol) and (ii) complete primary outcome and LCQ [18, 20] score at baseline. We will consider adjustments for time-varying post-randomization confounding [34] which will be predefined in the statistical analysis plan.

Based on a recent similar study, we assume that for patients who completed the study, only few data will be missing [12] and we expect 5–10% dropouts. The reason for missing data and whether it might be at-random or not will be examined according to the European Medicines Agency (EMA) guidelines [35]. If missing data is assumed to be “not at random”, sensitivity analyses will be performed. If missing data is of type “missing completely at random” or “missing at random” data will be imputed using the method of multiple imputation by chained equations (MICE). Missing data of all variables that are used in the statistical model to test the hypothesis will be imputed. Data of all available variables will be used for imputation. Multiple imputation will be performed using the R package “mice” [36]. The imputation procedure will be further defined in the SAP.

### Plans to give access to the full protocol, participant level-data and statistical code {31c}

The full study protocol which was approved by the Ethics Committee for North-western and Central Switzerland is available in the Supplementary material. Metadata describing the type, size and content of the datasets will be shared along with the study protocol and eCRF in a public repository (*dataverse.harvard.edu*). Additionally, the OSPIC eCRF templates designed for the study will be uploaded on the MDM-Portal (Medical Data Models) at medical-data-models.org. All variables in the OSPIC study eCRF will be annotated by their Unified Medical Language System Concept Unique Identifier (UMLS CUI) to improve findability for other clinicians.

The Department of Clinical Research of the University Hospital Basel (DKF) will act as an independent Data Access Committee (DAC) and store on secure servers the Clinical Data Management Application (CDMA) at the time of publication. Independent and external researchers from the study team can seek to access the data for reuse in other projects by submitting a study synopsis to the DFK curator at dkf.unibas.ch/contact. It is the responsibility of those researchers to seek a new approval for future studies from the ethics committee.

## Oversight and monitoring

### Composition of the coordinating centre and trial steering committee {5d}

The study is coordinated by the Centre for Primary Health Care at the University of Basel, led by Prof. Dr. med. Andreas Zeller. For the purposes of the OSPIC trial, the Centre for Primary Health Care works in collaboration with research partners at the Institute of Primary and Community Care, Lucerne, and the Department of Clinical Research, University Hospital Basel. This is an investigator-driven study conducted under the supervision of Prof. Dr. med. Andreas Zeller, Prof. Dr. Jörg Daniel Leuppi and Dr. Stefan Essig. Prof. Dr. Andreas Zeller is the Principal Investigator for the study and the main responsible for the entire project. Prof. Dr. Leuppi and Dr. Essig are also responsible with overseeing the conduct of the study.

The CTU at the University Hospital Basel is tasked with handling the data management system and performing monitoring activities. The CTU will provide an electronic data capture solution (secuTrial® database) for the storage of the participant CRFs. The CTU Basel is also responsible with the development, testing and deployment of the Clinical Data Management Application (CDMA) and with the preparation and implementation of a Data Management Plan (DMP), as reviewed and approved in their final versions by the Sponsor-Investigator.

Prof. Dr. med. Andreas Zeller will be involved in every step connected to this study including being responsible for project development and implementation, obtaining the collaboration of general practices for recruitment and enrolment of participants, interpretation of data, writing of scientific papers and study reports, etc.

### Composition of the data monitoring committee, its role and reporting structure {21a}

Data monitoring will be performed by the CTU of the University Hospital Basel and will be carried out according to the Standard Operating Procedures (SOPs) of the CTU and on the basis of the monitoring plan, agreed upon with the Sponsor.

### Adverse event reporting and harms {22}

Potential side effects and complications from corticosteroid will be systematically recorded during the trial. A recently published retrospective cohort study showed that even a short time use of corticosteroids increases the incidence of severe adverse events such as sepsis or venous thromboembolism [25]. Safety issues, even with short time corticosteroids regimens, are crucial and the study uses a follow-up schedule lasting up to 3 months from randomization to capture and assess risks to participants. Data about AE, including the events of special interest listed in the follow-up CRF (e.g. increase in appetite, weight gain, insomnia, fluid retention, and changes such as feeling irritable and anxious), and all SAE will be collected, fully investigated and documented in source documents and individual participants’ CRF for the entire duration of the study. The occurrence of AE and SAE will be routinely recorded by study staff at the 7, 14 and 28 days and at the 3 months follow-up calls. Participants will be informed and asked to immediately contact the GP or the study team in the event of any possible side-effects. In case the GPs cannot be reached, participants should visit the nearest hospital. For safety reasons, the study team will inform the corresponding GP about every reported event to ensure patient follow-up is arranged as soon as possible. GPs will be asked to follow-up all patients with suspected AE of interest or SAE. GPs and research staff will be instructed to document time of onset, duration, resolution and actions to be taken, as well as an assessment of intensity and relationship of event with study treatment. All participant SAEs captured during the follow-up interviews or reported to the GP will be transmitted within a maximum of 24 h to the Sponsor-Investigator. New information on participants becoming pregnant during the study intervention or within 30 days after taking the medication must also be reported to the Sponsor within 24 h and requires safety-related measures. The course and outcome of the pregnancy should be followed up carefully with the GP, and any abnormal outcome regarding the mother or the child should be documented and reported. Moreover, suspected new risks to participants and new relevant aspects regarding any known adverse reactions that require safety-related measures must be reported to the Sponsor within 24 h. All participating Investigators must also be informed by the Sponsor about all safety signals, including the occurrence of suspected unexpected serious adverse reactions. The study team is responsible for evaluating AE of interest and SAE according to the WHO-UMC causality categories [22]. When necessary, these will be reported to the EKNZ within 7 days. SAE assessments will be carried according to the severity grading scale used for adverse events occurring during trials: grade 1—mild, grade 2—moderate, grade 3—severe, grade 4—life-threatening, grade 5—death [37]. An annual safety report based on information from all participating GP practices will be prepared by the Sponsor-Investigator and submitted every year for the duration of the study to the EKNZ and to Swissmedic.

### Frequency and plans for auditing trial conduct {23}

Access to study documentation and data is allowed for the purposes of audit by regulatory authorities, which is independent from the investigators and Sponsor. Data and sites monitoring will be carried by the CTU of the University Hospital Basel according to the study monitoring plan.

### Plans for communicating important protocol amendments to relevant parties (e.g. trial participants, ethical committees) {25}

Mandatory reporting to the EKNZ and the regulatory authority (Swissmedic) will be carried, and we will seek approval prior to implementing any changes to the research protocol or to research activities. We will report changes to eligibility criteria, outcomes and unanticipated problems involving risks to participants, including the planned or premature study end. All changes will have to be first approved by the Sponsor and then reported. Necessary changes made to the protocol that are meant to eliminate apparent immediate risks to participants will be reported as soon as possible after they occur. After ethics review and prior to implementation, investigators will be informed in writing about any changes to protocol. Additionally, the OSPIC trial is registered on the international trial register clinicaltrials.gov (NCT04232449) and the Swiss national register kofam.ch (SNCTP000003644) where we will provide a publicly available synopsis of the study protocol. Information in the trial registry entries will be kept up-to-date and completed with study results after the completion of the trial.

## Dissemination plans {31a}

Study results will be published in a peer-reviewed medical journal, independent of the outcomes and conclusions. All results from this study will be published in aggregated and anonymized way. Manuscripts submitted for peer-review and presentations of any results will adhere to relevant reporting guidelines for publication as put forth by the EQUATOR-network [15, 31, 38–40]. Authorship to publications will be granted according to the rules of the International Committee of Medical Journal Editors (ICMJE) [41]. Additionally, at the end of the study, the research team will also update the systematic review on treatments for subacute cough [7] to include the data from the OSPIC trial.

## Discussion

Cough is often a nonspecific symptom of respiratory disease requiring complex differential diagnosis strategies, which raise challenges for both physicians and patients [42]. A recent RCT reported the effectiveness of a chronic cough management algorithm in paediatric community care and its usefulness in easily identifying causes of chronic cough by using this tool. Although the trial showed the algorithm’s potential in ensuring quick and appropriate management, it did not report outcomes from drug interventions [43]. Seeking medical advice for cough is the most common reason for presentation to primary care practices worldwide [1], and in the USA alone between 2001 and 2002, there were around 600,000 general practitioner or outpatient setting visits made due to cough associated with a previous respiratory infection [44]. Post-infectious cough has a broad impact on personal health and well-being [2] and bears relevant socioeconomic costs. Worldwide yearly estimated spending for over-the-counter (OTC) antitussives was around $4 billion in 2008 [45]. While the price of OTC drug purchases and the costs to the healthcare system, including the number of doctor’s appointments and associated expenses, are significant, a disproportionate higher cost is incurred from loss of productivity. In the UK, estimates for healthcare and medication costs are at £104 million, while losses resulting from leave of absence from work reach approximately £900 million [46].

Treatment decisions for patients with prolonged cough are complicated by patients’ anxiety and expectations. Physicians may experience pressure to prescribe antibiotics, despite no supporting recommendations for this course of treatment [4]. Inhaled corticosteroids and orally administered montelukast are available treatment options for post-infectious cough. The German Respiratory Society’s updated guidelines from 2020 also recommend taking inhaled corticosteroids for about 2 weeks for treating subacute cough [47], but evidence of benefit is weak [7, 11]. In a study in general practice, no benefit from montelukast therapy was found in patients with post-infectious cough [24]. As of April 2020, the Food and Drug Administration issued a fifth and its highest warning (boxed warning) for risk of neuropsychiatric events associated with montelukast [48]. This regulatory measure has a potentially considerable impact on physician’s risk-benefit analysis for prescribing montelukast, including for patients with post-infectious cough. As there is no established evidence-based treatment option for this very frequent condition in primary care, only a well-conducted randomized placebo-controlled trial can determine a safe and efficient therapy. Our study aims to fill this gap by determining the benefits and harms of oral corticosteroids in the treatment of patients with post-infectious cough enrolled in an RCT carried in a primary care setting. We selected prednisone for this trial because it is a well-established oral treatment for asthma, different allergic and other respiratory conditions [49]. Additionally, it is a low-cost therapy and proving its effectiveness and safety has significant cost reduction implications for treating cough.

Recruitment for the OSPIC trial, planned for early spring 2020, has been delayed due to the current SARS-CoV-2 pandemic [26]. The first reported case in Switzerland was at the end of February and was followed by extreme public health measures to stop the spread. General practitioners’ activity experienced disruption following the recommendation that patients should call their doctor in case of symptoms and for referrals for testing or hospitalization [30]. At the same time, research activities, administrative services and management for clinical studies are severely impacted by this public health emergency. New clinical studies were suspended, running trials were halted and research reviews prioritized protocol submissions on SARS-CoV-2 [50]. Assessing the pandemic situation over the summer and early autumn, we decided to open the GP practices recruitment by end of September 2020. Provided that researchers strictly adhere to hygiene measures, the University of Basel encouraged the research teams to resume study activities in July 2020. In order to respect the on-going pandemic situation, we added a question to the GPs’ baseline questionnaire asking whether a SARS-CoV-2 nasopharyngeal swab specimen had been obtained and asked the result of the test (positive/negative). A positive SARS-CoV-2 PCR test is not considered as an exclusion criterion. We did not change the schedule of the follow-up visits. There are limitations to this trial. We use a self-reporting tool (the LCQ) to measure cough-related quality of life and cough resolution, which is a subjective measurement. Other tools, such as frequency monitors, can improve cough measurement objectivity, but have significant limitations. Cough monitors are unreliable in distinguishing cough sounds from speech and other noises and require manual assessments that are impractical in the context of a trial in a primary care setting [51]. The LCQ is one of the most widely used health status questionnaires for adults suffering from cough [51] and is appraised by users as highly relevant, scoring above other similar and commonly used cough measures [20]. It is a patient-derived, valid, reliable and useful questionnaire for outcome measurement in clinical care and research activity [20]. This makes results from this study comparable with those from other studies and mitigates the substantial limitation that would result from an artificial research environment involving cough monitors.

Overall, short-term use of oral corticosteroids for post-infectious cough was not previously assessed in an RCT [7]. This trial will determine the clinical effectiveness of oral corticosteroids for the treatment of post-infectious cough and may establish the first treatment option with clear patient-relevant benefits and at low-costs for this common condition.

## Trial status

The Protocol version approved by the responsible ethics committee at the time of submission is 2.1/29.01.2020 (see Supplement 2). Participant recruitment is anticipated to begin in fall of 2020 and is estimated to resume after 18 months, around March 2022.

## Supplementary Information

**Additional file 1.** Ethical approval documents: a copy translated into English and a copy of the original document in German

**Additional file 2.** Copy of the original funding documentation, Swiss National Science Foundation (SNSF), Investigator Initiated Clinical Trials grant (IICT 2018 call, 33IC30_179657 3)

**Additional file 3.**

## Abbreviations

AE: Adverse events
CRF: Case report form
COPD: Chronic obstructive pulmonary disease
CDMA: Clinical data management application
CTU: Clinical Trial Unit
CI: Confidence intervals
DAC: Data Access Committee
DMP: Data Management Plan
DKF: Department of Clinical Research of the University Hospital Basel
eCRF: Electronic Case Report Forms
EKNZ: Ethics Committee for North-western and Central Switzerland
EMA: European Medicines Agency
FAS: Full analysis set
GP: General practitioners/practices
ITT: Intention-to-treat
ICC: Intra-cluster correlation
IPC: Intra-patient correlation
ICH-GCP: International Conference on Harmonisation – Good Clinical Practice
ISF: Investigator Site File
LCQ: Leicester Cough Questionnaire
MDM: Medical data models
MCID: Minimal clinically important difference
OTC: Over-the-counter
PPS: Per-protocol data set
RCT: Randomized controlled trial
SAE: Serious adverse events
SD: Standard deviation
SOP: Standard Operating Procedures
SAP: Statistical analysis plan
UMLS CUI: Unified Medical Language System Concept Unique Identifier
QoL: Quality of life
WHO-UMC: World Health Organization-Uppsala Monitoring Centre
