# Supplementary material for: Oral corticosteroids for post-infectious cough in adults: study protocol for a double-blind randomized placebo-controlled trial in Swiss family practices (OSPIC trial)
Source: Trials. 2020 Nov 23;21:949. doi: 10.1186/s13063-020-04848-4 (PMC7681763; doi:10.1186/s13063-020-04848-4)
Supplement: Supplementary file 1 — Additional file 1. Ethical approval documents: a copy translated into English and a copy of the original document in German [file 13063_2020_4848_MOESM1_ESM.pdf]

„Orale Kortikosteroide für die Behandlung von post-infektiösem Husten bei Erwachsenen“

“Oral corticosteroids for post-infectious cough in adults: A double-blind randomised placebo-controlled trial in Swiss family practices (OSPIC Trial)”

Diese Studie wird organisiert von: **Prof. Dr. med. Andreas Zeller**
Leiter Universitäres Zentrum für Hausarztmedizin beider Basel
Universität Basel
4410 Liestal

Sehr geehrte Dame, sehr geehrter Herr

Wir möchten Sie anfragen, ob Sie an einer klinischen Studie teilnehmen wollen. Im Folgenden wird Ihnen dieses Studienvorhaben dargestellt: Zunächst in einer kurzen Zusammenfassung, damit Sie wissen, um was es geht, anschliessend in einer detaillierten Beschreibung.
Es wird dabei die männliche Form verwendet, selbstverständlich sind alle Geschlechter angesprochen.

Zusammenfassung

1	**Ziel der Studie**<br>Die Studie untersucht die Behandlung eines störenden Hustens, dem sogenannten post-infektiösen Husten, der im Anschluss an eine Infektion der oberen Atemwege auftreten kann und mehr als 3 aber weniger als 8 Wochen andauert. Bisher ist für diese Hustenform keine wissenschaftlich nachgewiesen wirksame Therapie verfügbar. Wir führen diese Studie mit einer Kortison Therapie durch, um eine effektive Behandlung für diesen Husten zu finden.
2	**Auswahl**<br>Wir lassen Ihnen diese Informationsschrift zukommen da Sie momentan unter post-infektiösem Husten leiden. Wenn der Husten bereits mehr als 2 Monate bei Ihnen anhält, können Sie nicht an dieser Studie teilnehmen. Auch nicht an dieser Studie teilnehmen können Sie, falls bei Ihnen eine Schwangerschaft besteht oder Sie stillen. Ihr Hausarzt prüft, ob Sie zur Teilnahme an der Studie berechtigt sind.
3	**Allgemeine Informationen zur Studie**<br>Bislang gibt es keine Standardtherapie für Patienten die unter post-infektiösem Husten leiden. Sollten Sie sich für die Teilnahme an unserer Studie entscheiden, werden Sie zufällig einer von zwei Behandlungsgruppen zugeteilt. Entweder erhalten Sie über 5 Tage eine Behandlung mit Kortison (40mg) oder Sie erhalten Placebo-Tabletten (Scheinmedikament). Weder Sie noch der behandelnde Arzt wissen, welche der beiden Möglichkeiten Sie bekommen werden. Das Medikament enthält den Wirkstoff Prednisonum. Es handelt sich dabei um ein in der Schweiz zugelassenes Medikament. Es wird bislang jedoch nicht für die Behandlung von post-infektiösem

	Husten eingesetzt.<br>Im Rahmen dieser Studie sollen im Ganzen 204 Teilnehmende aus 42 Hausarztpraxen in der Deutschschweiz rekrutiert werden.
4	**Ablauf**<br>Wenn Sie sich nach einer Infektion der oberen Atemwege mit post-infektiösem Husten bei Ihrem Hausarzt vorstellen, wird Ihnen Ihr Hausarzt von dieser Studie berichten und bei Interesse, dieses Studieninformation aushändigen. Sollten Sie sich für die Teilnahme an unserer Studie entscheiden, prüft der Hausarzt, ob Sie alle Kriterien für die Teilnahme an der Studie erfüllen. Wenn Sie berechtigt sind, erhalten Sie entweder eine fünftägige Therapie mit Prednison oder 5 Tage mit Placebo-Tabletten. Die Einteilung in die Gruppe «Prednison» oder «Placebo» wird zufällig (randomisiert) erfolgen.<br><br>**<u>Für Frauen, die an der Studie teilnehmen möchten</u>: Wenn Sie schwanger sind oder schwanger sein könnten oder eine Schwangerschaft planen in den nächsten 3 Monaten, können Sie nicht an dieser Studie teilnehmen.**<br><br>Sie werden auch einen Fragebogen ausfüllen (Dauer: 5-10 Minuten). Sie bekommen das Studienmedikament mit nach Hause und nehmen es während der folgenden 5 Tage selbstständig ein. Danach werden sie am 7., 14. und 28. Tag sowie 3 Monate nach Ihrem Arztbesuch telefonisch von einem Studienmitarbeiter kontaktiert, um den Fragebogen erneut zu beantworten. Dies dient der Verlaufskontrolle und ermöglicht abzuschätzen, ob die Kortisontherapie die gewünschte Wirkung hat. Weiter werden Ihnen Fragen zur Medikamenteneinnahme, zum Hustenverlauf und zu Nebenwirkungen gestellt (Zeitaufwand jeweils etwa 15 Minuten).<br>Die eventuell im Verlauf nötigen Besuche bei Ihrem Hausarzt sind unabhängig von der Studie. Während der telefonischen Verlaufskontrollen werden Sie nach eventuellen Arztbesuchen gefragt.
5	**Nutzen**<br>Mit Ihrer Studienteilnahme können Sie dazu beitragen, dass Sie und andere Patienten mit post-infektiösem Husten künftig von einer effektiven Therapie profitieren können.
6	**Rechte**<br>Sie entscheiden freiwillig, ob Sie an der Studie teilnehmen wollen oder nicht. Ihre Entscheidung hat keinen Einfluss auf Ihre medizinische Behandlung und Sie müssen diese Entscheidung nicht begründen.
7	**Pflichten**<br>Wenn Sie teilnehmen, bitten wir Sie, sich strikt an die vorgegebene Dosierung und Einnahmedauer der Medikamente zu halten, sowie an den gemeinsam abgestimmten Terminen für die telefonischen Befragungen zur Verfügung zu stehen.
8	**Risiken**<br>Wie bei fast jeder medikamentösen Behandlung, besteht das Risiko, dass Nebenwirkungen auftreten können. Bei der Behandlung mit Prednison sind folgende Nebenwirkungen bekannt: Appetitsteigerung, Antriebssteigerung; Störung des Zuckerstoffwechsels; Entgleisung eines gut eingestellten Diabetes, Kopfschmerzen, leicht und schwere Infekte oder Thrombosen. Diese Nebenwirkungen sind aber bei einer kurzen Therapiedauer von 5 Tagen unwahrscheinlich.
9	**Andere Behandlungsmöglichkeiten**<br>Ihr Hausarzt wird Sie beraten, welche anderen Möglichkeiten zu Ihrer Behandlung bestehen.
10	**Ergebnisse**

	Bei Studienergebnissen während der Studie werden Sie informiert, wenn diese Ergebnisse für Sie gesundheitlich wichtig sind. Falls Sie dies nicht möchten, informieren Sie bitte Ihren Hausarzt.
11	**Vertraulichkeit von Daten**<br>Wir halten alle gesetzlichen Regeln des Datenschutzes ein und alle Beteiligten unterliegen der Schweigepflicht. Ihre persönlichen und medizinischen Daten werden verschlüsselt verwendet und geschützt. Die Daten werden für andere Forschungsprojekte weiterverwendet, wenn Sie Ihr Einverständnis dafür geben.
12	**Rücktritt**<br>Sie können jederzeit von der Studie zurücktreten. Die bis dahin erhobenen Daten werden noch ausgewertet.
13	**Entschädigung**<br>Sie erhalten keine Entschädigung für die Teilnahme an dieser Studie. Jedoch wird Ihnen das Studienmedikament, sowie die Betreuung durch das Studienteam kostenfrei zur Verfügung gestellt.
14	**Haftung**<br>Die Versicherung Helvetia, Dufourstrasse 40, 9001 St. Gallen, kommt für Schäden im Rahmen der Studie auf.
15	**Finanzierung**<br>Die Studie wird vom Schweizerischen Nationalfonds (SNF) unterstützt und finanziert..
16	**Kontaktperson:**<br>Sie erhalten jederzeit auf alle Ihre Fragen Auskunft.<br><br>Prof. Dr. med. Andreas Zeller<br>Leiter Universitäres Zentrum für Hausarztmedizin beider Basel, Universität Basel<br>Kantonsspital Baselland<br>Rheinstr. 26<br>4410 Liestal<br>Tel: 061 925 20 75 oder 061 692 88 11<br>Email: OSPIC@ksbl.ch<br><br>**Wenn Sie Symptome haben** wie Fieber, Kopfschmerzen, Störungen des Zuckerstoffwechsels, Schlaflosigkeit, Schwindel oder Schwäche, Bauch- oder Brustschmerzen, Angst oder Depression, Hautausschlag, Nasennebenhöhlenentzündung, Halsschmerzen, Schnupfen, Juckreiz, Muskelschmerzen, Engegefühl in der Brust, Herzrasen, geschwollene Lymphknoten, Bindehautentzündung, unklare Infektion oder das Gefühl eine Thrombose zu haben, oder andere (unerwartete) Symptome auftreten, dann<br><br>1. → **Wenden Sie sich bitte unverzüglich an Ihren Hausarzt oder Hausärztin**<br>2. → **Falls dieser nicht verfügbar ist**, suchen Sie bitte eine Notfallstation in Ihrer Nähe auf.<br>3. → Sie haben auch die Möglichkeit, **die Notfallnummer der OSPIC Studie** zu wählen. Diese lautet: **077 508 39 39**. Sie erhalten jederzeit Auskunft auf Ihre Fragen.

Detailliertere Information zur Studie

„Orale Kortikosteroide für die Behandlung von post-infektiösem Husten bei Erwachsenen“

Wir sind Mitarbeitende der Medizinischen Universitätsklinik des Kantonsspitals Baselland, des Instituts für Hausarztmedizin beider Basel (uniham-bb) der Universität Basel und des Instituts für Hausarztmedizin der Universität Luzern. Gemeinsam sind wir für die Ihnen im Folgenden vorgestellte Studie verantwortlich.

1. Ziel der Studie

Wir wollen untersuchen, ob Prednison bei post-infektiösem Husten wirksam ist. Das Medikament enthält den Wirkstoff *Prednisonum*. Für die Studie wird vor allem der entzündungshemmende Effekt von Prednison ausgenützt. Es handelt sich dabei um ein in der Schweiz zugelassenes Medikament. Es wird bislang jedoch nicht für die Behandlung von post-infektiösem Husten eingesetzt, wird aber zum Beispiel in gleicher Dosierung und Therapiedauer erfolgreich für die Behandlung bei einem Asthmaanfalles eingesetzt.

2. Auswahl

Es können alle Personen teilnehmen, welche sich bei ihrem Hausarzt vorstellen und an post-infektiösem Husten leiden. Ein post-infektiösen Husten dauert mehr als 3, aber weniger als 8 Wochen nach einer Infektion der oberen Atemwege dauert. Ausserdem müssen sie älter als 18 Jahre alt sein und in der Lage, der Teilnahme an dieser Studie zuzustimmen. Nicht teilnehmen hingegen dürfen Personen, die bereits eine andere Atemwegserkrankung haben, immun-geschwächt sind oder im letzten Monat eine Kortikosteroid-Behandlung erhalten haben. Schwangere und stillende Frauen und Leute mit bewiesener Osteoporose können ebenfalls nicht teilnehmen.

3. Allgemeine Informationen

- Bei dieser Studie handelt es sich um eine nationale Studie in der Nordwest- und Zentralschweiz, die in Zusammenarbeit mit verschiedenen Hausärztinnen und Hausärzten durchgeführt wird. Bisher ist für diesen post-infektiösen Husten keine wissenschaftlich nachgewiesen wirksame Therapie verfügbar ist. Als Teilnehmender werden Sie entweder eine fünftätige Behandlung mit Prednison oder mit Placebo-Tabletten erhalten. Sie müssen einmal am Tag zwei Tabletten (Prednison oder Placebo) einnehmen. Die Einteilung in die Gruppe «Prednison» oder «Placebo» wird zufällig erfolgen und weder Sie noch ihr Hausarzt wissen, zu welcher Gruppe Sie gehören.

- Die Studie wird ab Juni 2019 über 36 Monate durchgeführt. Im Rahmen dieser Studie sollen im Ganzen 204 Teilnehmende rekrutiert werden. Wir machen diese Studie so, wie es die Gesetze in der Schweiz vorschreiben. Ausserdem beachten wir alle international anerkannten Richtlinien. Die zuständige Kantonale Ethikkommission und Swissmedic (Schweizerische Arzneimittelbehörde)

haben die Studie geprüft und bewilligt. Eine Beschreibung dieser Studie finden Sie auch auf der Internetseite des Bundesamtes für Gesundheit: www.kofam.ch.

4. Ablauf

Wenn Sie sich nach einer Infektion der oberen Atemwege mit post-infektiösem Husten bei Ihrem Hausarzt vorstellen, wird Ihnen Ihr Hausarzt von dieser Studie berichten und bei Interesse, dieses Studieninformation aushändigen. Sollten Sie sich für eine Teilnahme an unserer Studie entscheiden, prüft der Hausarzt, ob Sie alle Kriterien für die Teilnahme an der Studie erfüllen.

Für Frauen, die an der Studie teilnehmen möchten:

Wenn Sie schwanger sind oder schwanger sein könnten oder eine Schwangerschaft planen in den nächsten 3 Monaten, können Sie nicht an dieser Studie teilnehmen.

Wenn Sie berechtigt für die Teilnahme an der Studie sind, wird Sie Ihr Hausarzt detailliert über die Studie informieren und alle bestehenden Fragen beantworten.

Im Anschluss wird Ihnen die Studienmedikation übergeben, die Sie dann fünf Tage, einmal am Tag, einnehmen müssen. Bei diesem Termin werden Sie auch einen Fragebogen zum Thema Husten ausfüllen. Diese Befragung wird ungefähr 5-10 Minuten dauern. Sie werden gebeten am Schluss der Befragung Ihre Telefonnummer und Email für die telefonische Nachbefragung anzugeben. Ihre Telefon- und E-Mail-Informationen werden bei Ihrem Hausarzt gespeichert und anschliessend sicher an das Studienzentrum in Liestal und an die sogenannte «Clinical Trial Unit» (Studienkoordinationsstelle) des Universitätsspital Basel weitergeleitet.

Die telefonische Nachbefragung wird am Tag 7, Tag 14, am Tag 28 und nach 3 Monaten nach Studieneinschluss statt und in der Regel ungefähr 15 Minuten dauern. Insgesamt umfasst sie Gesamtbefragungszeit circa eine Stunde. Nach Beendigung der Einnahme der Studien-Tabletten (nach 5 Tagen) bitten wir Sie, das leere Medikamentenglas ihrem Hausarzt zurückzubringen. Sie können das auch anlässlich einer nächsten Konsultation bei ihrem Hausarzt tun. Wenn Sie die Studie vorzeitig beenden oder die nachträglichen Telefonate nicht beantworten, kann sich das Studienteam an den Hausarzt wenden, um Informationen zu möglichen Hausarztbesuchen oder Spitalaufenthalten zu erhalten. Auch dann möchten wir sie bitten, dass Glas mit den Studien-Medikamenten in Ihre Hausarztpraxis zurückzubringen.

Allenfalls zusätzlich nötige Besuche bei Ihrem Hausarzt sind unabhängig von der Studie. Während der telefonischen Verlaufskontrollen werden Sie nach eventuellen Arztbesuchen gefragt.

Es kann sein, dass wir Sie von der Studie vorzeitig ausschliessen müssen. Dies kann geschehen, wenn Sie beispielsweise schwanger werden oder bei Ihnen eine (schwerwiegende) Erkrankung festgestellt wird. In diesem Fall werden Sie zu Ihrer Sicherheit noch einmal von Ihrem Hausarzt untersucht. Bitte bringen Sie dann alle Studien-Medikamente mit, welche wir Ihnen gegeben haben.

Auf der nächsten Seite ist der Studienablauf ist grafisch dargestellt:

Der Patient/ Die Patientin kommt mit einem störenden Husten (sogenannter post-infektiöser Husten)* zum Hausarzt

*der im Anschluss an einer Infektion der oberen Atemwege aufgetreten ist und mehr als 3 aber weniger als 8 Wochen andauert

↓

Diagnose beim Hausarzt

Ausgabe Studieninformation	Umfassende Information und Beantwortung aller Fragen zur Studie durch den Hausarzt

↓

Studieneinschluss mit Einverständnis des Patienten

Ausgabe Studienmedikation (Prednison- oder Placebo-Tabletten) für 5 Tage	Einen Fragebogen mit dem Hausarzt ausfüllen (Dauer: 5-10 min)	Terminabsprache für das 1. Telefonat (Fragebogenerhebung durch die Forschungsmitarbeiterin)

↓

4 Telefonate mit Forschungsmitarbeiterin

Fragebogenerhebung und weitere Fragen zu Medikamenteneinnahme, Hustenverlaufes, Nebenwirkungen (Gesamtbefragungszeit: ca. 1 Stunde)

↓

**1. Telefonat 7 Tage nach Studieneinschluss** (Dauer: ca. 15min)	**2. Telefonat 14 Tage nach Studieneinschluss** (Dauer: ca. 15min)	**3. Telefonat 28 Tage nach Studieneinschluss** (Dauer: ca. 15min)	**4. Telefonat (3 Monate nach Studieneinschluss)** (Dauer: ca. 15min)

5. Nutzen

Sie werden persönlich keinen Nutzen von der Teilnahme an der Studie haben. Wenn Sie bei dieser Studie mitmachen, können Sie jedoch dazu beitragen, dass Sie und andere Patienten mit post-infektiösem Husten künftig von einer effektiven Therapie profitieren könnten. Die Resultate können wichtig sein für andere, die dieselbe Krankheit haben. Bislang gibt es keine Standardtherapie für Patienten mit diesem Krankheitsbild.

6. Rechte

Sie nehmen freiwillig teil. Wenn Sie nicht mitmachen oder später Ihre Teilnahme zurückziehen wollen, müssen Sie dies nicht begründen. Ihre medizinische Behandlung/Betreuung ist dann unabhängig von Ihrem Entscheid in jeder Hinsicht gewährleistet. Sie dürfen jederzeit Fragen zur Studienteilnahme stellen. Wenden Sie sich dazu bitte an die Person, die am Ende dieser Information genannt ist.

7. Pflichten

Als Teilnehmer ist es notwendig, dass Sie

- sich an die notwendigen Vorgaben und Anforderungen der Studie durch den Prüfplan halten. Besonders wichtig ist die korrekte und vollständige Einnahme der Studienmedikamente.
- ihren Hausarzt über den Verlauf der Erkrankung informieren und ihm neue Symptome, neue Beschwerden und Änderungen im Befinden zu melden (auch nach Studienende/-abbruch, z.B. bis die unerwünschte Wirkung abklingt);
- ihren Hausarzt über die gleichzeitige Behandlung und Therapie bei einem anderen Arzt und über die Einnahme von Medikamenten (auch Medikamente der Komplementärmedizin) zu informieren.

8. Risiken und Belastungen für die Teilnehmenden

Wesentliche Unannehmlichkeiten: Durch die Visite beim Hausarzt und die telefonische Befragung entstehen für Sie eine gewisse zusätzliche zeitliche Belastung. Das Forschungsteam bemüht sich den Aufwand für die Studienteilnehmenden so gering wie möglich zu halten.

Unerwünschte Wirkungen des Medikamentes: Glukokortikoide - wie das in der Studie verwendete Prednison - beeinflussen viele Organe und Organsystem. Deswegen sind auch die möglichen Nebenwirkungen vielfältig. Die Nebenwirkungen unter Therapie hängen stark von Dosis und Dauer der Therapie ab. Die Anwendungsdauer von 5 Tagen in dieser Studie ist sehr kurz. Nebenwirkungen bei der systemischen (Einnahme als Tablette) Anwendung von Prednison sind bei der kurzzeitigen Einnahme unwahrscheinlich, aber nicht ausgeschlossen. Bei einer kurzfristigen Behandlung mit Prednison in einer Dosierung von 40mg ist das Risiko von unerwünschten Wirkungen grundsätzlich gering.

- Mögliche Nebenwirkungen *von Prednison*: Es kann zu einer Erhöhung des Blutzuckers, in der Regel am höchsten 4–6 Stunden nach Einnahme, kommen. Bei Patienten mit gut eingestelltem Diabetes kann es durch die kurzzeitige (5 Tage) Einnahmen zu einer Entgleisung (Erhöhung) des Blutzuckers kommen. Weitere Nebenwirkungen sind Appetitsteigung, Antriebssteigerung, Schlafstörungen oder erhöhte Blutdruckwerte.

- Schwere Nebenwirkungen sind selten und treten vermehrt bei länger (>2-3 Wochen) dauernder Therapie auf. Hierzu gehören Infektionen jeglicher Art und Schweregrad, Thrombosen, erhöhte Wasseransammlung im Körper Hautveränderungen (wie Gewebeschwund,

Wundheilungsstörungen, Steroidakne), Muskelabbau, Knochenabbau (Osteoporose), Magengeschwüre und Depressionen.

Wenn Sie Symptome haben wie Fieber, Kopfschmerzen, Störungen des Zuckerstoffwechsels, Schlaflosigkeit, Schwindel oder Schwäche, Bauch- oder Brustschmerzen, Angst oder Depression, Hautausschlag, Nasennebenhöhlenentzündung, Halsschmerzen, Schnupfen, Juckreiz, Muskelschmerzen, Engegefühl in der Brust, Herzrasen, geschwollene Lymphknoten, Bindehautentzündung, unklare Infektion oder das Gefühl eine Thrombose zu haben, oder andere (unerwartete) Symptome auftreten, dann

1. → **Wenden sie sich bitte unverzüglich an Ihren Hausarzt oder Hausärztin.**
2. → **Fall dieser nicht verfügbar ist**, suchen Sie bitte eine Notfallstation in Ihrer Nähe auf.
3. → Sie haben auch die Möglichkeit, **die Notfallnummer der OSPIC Studie** zu wählen. Diese lautet: **077 508 39 39**. Sie erhalten jederzeit Auskunft auf Ihre Fragen.

Für Frauen, die schwanger werden können

Frauen im gebärfähigen Alter mit einem bestehenden Wunsch oder Risiko einer Schwangerschaft und ungenügender Verhütung können nicht in die Studie eingeschlossen werden.

Frauen, die zum Einschlusszeitpunkt stillen, sind von einer Studienteilnahme ausgeschlossen.

Frauen, welche eine der folgenden Schwangerschaftsverhütungen anwenden, setzen eine genügende Verhütung ein und können in die Studie eingeschlossen werden:

- «Antibabypille»
- Verhütungsstäbchen unter der Haut (Implanon®)
- Hormonspirale (z.B. Mirena®)- oder Kupferspirale
- «Dreimonatsspritze» (z.B. Depo Provera®) erhalten
- Frauen, deren Partner konsequent Kondome verwendet

Frauen, die an der Studie teilnehmen, müssen diese Schwangerschaftsverhütung während der gesamten Studiendauer ohne Unterbrechung anwenden.

Sollten Sie im Verlauf der Studie dennoch schwanger werden, bitten wir Sie darum, Ihren Hausarzt über eine allfällige Schwangerschaft, deren Verlauf und Ausgang genau zu informieren.

9. Andere Behandlungsmöglichkeiten

Sie müssen bei dieser Studie nicht teilnehmen. Wenn Sie nicht mitmachen, kann Sie Ihr Hausarzt bezüglich anderer Behandlungsmöglichkeiten beraten.

10. Ergebnisse aus der Studie

Der Hausarzt wird Sie während der Studie über alle neuen Erkenntnisse informieren, die den Nutzen der Studie oder Ihre Sicherheit und somit Ihre Einwilligung zur Teilnahme an der Studie beeinflussen können. Sie werden die Information mündlich und schriftlich erhalten.

11. Vertraulichkeit der Daten

Für diese Studie werden Ihre persönlichen und medizinischen Daten erfasst. Nur sehr wenige Fachpersonen werden Ihre unverschlüsselten Daten sehen, und zwar ausschliesslich, um Aufgaben im Rahmen der Studie zu erfüllen.

Bei der Datenerhebung zu Studienzwecken werden die Daten verschlüsselt. Verschlüsselung bedeutet, dass alle Bezugsdaten, die Sie identifizieren könnten (Name, Geburtsdatum), gelöscht und durch eine Schlüssel-Liste ersetzt werden. Diese Schlüssel-Liste bleibt immer beim Hausarzt. Um die telefonischen Verlaufskontrollen durchführen zu können, wird eine Kopie der Schlüssel-Liste im Studienzentrum in Liestal und eine Kopie im Universitätsspital Basel aufbewahrt. Diejenigen Personen, die den Schlüssel nicht kennen, können daher keine Rückschlüsse auf Ihre Person ziehen. Bei einer Publikation sind die zusammengefassten Daten daher auch nicht auf Sie als Einzelperson rückverfolgbar. Ihr Name taucht niemals im Internet oder einer Publikation auf. Manchmal gibt es die Vorgabe bei einer Zeitschrift zur Publikation, dass Einzel-Daten (sogenannte Roh-Daten) übermittelt werden müssen. Wenn Einzel-Daten übermittelt werden müssen, dann sind die Daten immer verschlüsselt und somit ebenfalls nicht zu Ihnen als Person rückverfolgbar. Alle Personen, die im Rahmen der Studie Einsicht in Ihre Daten haben, unterliegen der Schweigepflicht. Die Vorgaben des Datenschutzes werden eingehalten und Sie als teilnehmende Person haben jederzeit das Recht auf Einsicht in Ihre Daten.

Es ist möglich, dass Ihre Daten für andere Untersuchungen zu einem späteren Zeitpunkt weiterverwendet werden oder später an eine andere Datenbank in der Schweiz oder ins Ausland für noch nicht näher definierte Untersuchungen (Weiterverwendung) versandt und verwendet werden. Diese andere Datenbank muss die gleichen Standards einhalten wie die Datenbank zu dieser Studie.

Möglicherweise wird diese Studie durch die zuständige Ethikkommission, die Arzneimittelbehörde Swissmedic oder durch die Institution, die die Studie veranlasst hat, überprüft. Der Hausarzt muss eventuell Ihre persönlichen und medizinischen Daten für solche Kontrollen offenlegen. Ebenso kann es sein, dass bei Schäden ausnahmsweise auch ein Vertreter der Versicherung Ihre Daten ansehen muss. Alle Personen müssen absolute Vertraulichkeit wahren.

Es ist möglich, dass Ihr Hausarzt kontaktiert wird, um Auskunft über Ihren Gesundheitszustand zu geben.

12. Rücktritt

Sie können jederzeit aufhören und von der Studie zurücktreten, wenn Sie das wünschen. Die bis dahin erhobenen Daten werden noch verschlüsselt auswertet, weil das ganze Projekt sonst seinen Wert verliert. Anschliessend werden die Daten anonymisiert, das heisst, der Code, der die Daten mit Ihnen verbindet, wird zerstört.

13. Entschädigung für Teilnehmende

Wenn Sie an dieser Studie teilnehmen, bekommen Sie dafür keine Entschädigung. Es entstehen Ihnen oder Ihrer Krankenkasse keine Kosten durch die Teilnahme.

14. Haftung

Die Institution (das Universitäre Zentrum für Hausarztmedizin beider Basel, Universität Basel, der Sponsor), die die Studie veranlasst hat und für die Durchführung verantwortlich ist, haftet für Schäden, welche Ihnen im Zusammenhang mit der getesteten Substanz oder Forschungshandlungen entstehen könnten. Die Voraussetzungen und das Vorgehen dazu sind

gesetzlich geregelt. Das Universitäre Zentrum für Hausarztmedizin (Rheinstrasse 26, 4410 Liestal) hat daher eine Versicherung bei der Versicherung (Helvetia Schweizerische Versicherungsgesellschaft AG, Dufourstrasse 40,9001 St. Gallen) abgeschlossen, um in einem möglichen Schadenfall für die Haftung aufkommen zu können.

Bei Schäden, die auf ein zugelassenes und gemäss dem medizinischen Standard angewendetes Heilmittel zurückzuführen sind, welche im Rahmen der Placebo-Verwendung auftraten oder auch bei Anwendung einer üblichen Therapie aufgetreten wären, greifen dieselben Haftungsregelungen wie bei einer Behandlung ausserhalb einer Studie.

Falls Sie einen Schaden erlitten haben, so wenden Sie sich bitte an Ihren Hausarzt oder rufen Sie die Studien Nummer an (077 508 39 39).

15. Finanzierung der Studie

Die Studie wird vollständig vom Schweizerischen Nationalfonds (SNF) bezahlt.

16. Kontaktperson(en)

Bei Fragen, Unsicherheiten oder Notfällen, die während der Studie oder danach auftreten, können Sie sich jederzeit an eine dieser Kontaktpersonen wenden.

Prof. Dr. med. Andreas Zeller
Leiter Universitäres Zentrum für Hausarztmedizin beider Basel, Universität Basel
Kantonsspital Baselland
Rheinstr. 26
4410 Liestal
Tel: 061 925 20 75 oder 061 692 88 11
Email: OSPIC@ksbl.ch

Die Notfallnummer der OSPIC Studie lautet: **077 508 39 39**

17. Glossar (erklärungsbedürftige Begriffe)

- **Was heisst „Placebo“?**
  Manche Menschen, die ein Medikament bekommen, werden nicht durch das Medikament gesund, sondern erfahren alleine durch die die Zuwendung und Fürsorge des Arztes eine Besserung. Das kann man daran erkennen, dass es ihnen besser geht, selbst wenn sie ein sogenanntes Scheinmedikament bekommen. Dieses Scheinmedikament sieht aus wie ein echtes Medikament und ist auch gleich verpackt. Tatsächlich ist aber in diesem Scheinmedikament gar kein Wirkstoff. Man nennt es „Placebo“. Manchmal behandelt man einen Teil der Teilnehmenden an einer klinischen Studie mit dem richtigen Medikament (mit dem Wirkstoff) und den anderen Teil mit einem solchen Placebo (ohne Wirkstoff). Dann kann man im Vergleich besser abschätzen, wie gut das Medikament tatsächlich wirkt oder ob die Besserung nur eintritt, weil die Menschen Zuwendung und Fürsorge erhalten. Manchmal entspricht die Besserung auch einfach dem natürlichen Verlauf der Krankheit.

- **Was heisst „randomisiert“?**
  Bei vielen Studien werden zwei oder mehrere unterschiedliche Arten der Behandlung verglichen. Zum Beispiel vergleicht man ein echtes Medikament mit einem Placebo. Man bildet dann zwei Gruppen von Teilnehmenden, die einen bekommen das echte Medikament und die anderen das Placebo. „Randomisieren“ bedeutet dann, dass ausgelost wird, wer in welche Gruppe kommt. Es ist bei einem solchen Test also Zufall, ob man das echte Medikament erhält oder das Placebo.

- **Was heisst "einfachblind" bzw. "doppelblind"?**
  Eine Studie zu "verblinden" (einfach oder doppelt) dient dazu, bessere und genauerer Ergebnisse zu erhalten. Von einer "einfachblinden" Studie spricht man, wenn bei der Studie entweder die Studienteilnehmer oder die Forschenden nicht wissen, ob ein Teilnehmer das echte oder das Scheinmedikament erhält. Wer was bekommt, lost eine Person aus, die nicht bei dem Test mitmacht.
  "Doppelblind" ist eine Studie dann, wenn weder die Teilnehmenden noch die Forschenden wissen, ob ein Studienteilnehmer das echte Medikament oder das Placebo erhält. Nur die unabhängige Person, die diese Zuordnung ausgelost hat, weiss, wer was erhält. Wenn der Test zu Ende ist, wird die "Verblindung" aufgelöst. In einem Notfall kann die "Verblindung" jederzeit auch früher aufgehoben werden.
  Eine Person, die weiss, dass sie das echte Medikament und nicht das Placebo erhält, achtet ganz anders auf Reaktionen des Körpers als jemand, der weiss, dass er nur das Placebo erhält. Dies kann dazu führen, dass Personen, die das echte Medikament erhalten, die Wirkung des Medikaments im Vergleich zu denjenigen, die nur das Placebo erhalten, überschätzen.

- **"doppelblind randomisierter, Placebo-kontrollierter klinischer Versuch":**
  Bei der Studie wird untersucht, wie gut das neue Medikament wirkt. Dazu werden die Teilnehmenden in (meist) zwei verschiedene Gruppen eingeteilt: die Teilnehmenden der einen Gruppe erhalten das Medikament, das untersucht wird. Die Teilnehmenden der anderen Gruppe bekommen das Placebo, also ein Scheinmedikament (welches aussieht wie ein richtiges Medikament, aber keinen Wirkstoff enthält). So kann man herausfinden, ob das Medikament eine Wirkung hat oder nicht.

Einwilligungserklärung

Schriftliche Einwilligungserklärung zur Teilnahme an einem Studienprojekt

- Bitte lesen Sie dieses Formular sorgfältig durch.
- Bitte fragen Sie, wenn Sie etwas nicht verstehen oder wissen möchten. Für die Teilnahme ist Ihre schriftliche Einwilligung notwendig.

**BASEC-Nummer (nach Einreichung):**	
**Titel der Studie**<br>**(wissenschaftlich und Laiensprache)**:	Oral corticosteroids for post-infectious cough in adults: A double-blind randomised placebo-controlled trial in Swiss family practices (OSPIC Trial)<br><br>Orale Kortikosteroide für die Behandlung von post-infektiösem Husten bei Erwachsenen
**verantwortliche Institution**<br>**(Sponsor mit Adresse**):	Prof. Dr. med. Andreas Zeller<br>Leiter Universitäres Zentrum für Hausarztmedizin beider Basel<br>Kantonsspital Baselland<br>Rheinstr. 26<br>4410 Liestal
**Ort der Durchführung**:	
**Verantwortlicher Prüfarzt am Studienort:**<br>Name und Vorname in Druckbuchstaben:	
**Teilnehmerin/Teilnehmer:**<br>Name und Vorname in Druckbuchstaben:<br>Geburtsdatum:	☐ weiblich ☐ männlich

- Ich wurde vom unterzeichnenden Hausarzt mündlich und schriftlich über den Zweck, den Ablauf der Studie mit dem Medikament Prednison oder Placebo über mögliche Vor- und Nachteile sowie über eventuelle Risiken informiert.
- Ich nehme an dieser Studie freiwillig teil und akzeptiere den Inhalt der abgegebenen schriftlichen Information. Ich hatte genügend Zeit, meine Entscheidung zu treffen.
- Meine Fragen im Zusammenhang mit der Teilnahme an dieser Studie sind mir beantwortet worden. Ich behalte die schriftliche Information und erhalte eine Kopie meiner schriftlichen Einwilligungserklärung.
- Ich wurde über mögliche andere Behandlungen und Behandlungsverfahren aufgeklärt.
- Ich bin einverstanden, dass die zuständigen Fachleute des Sponsors, der zuständigen Ethikkommission und der Arzneimittelbehörde Swissmedic zu Prüf- und Kontrollzwecken in meine unverschlüsselten Daten Einsicht nehmen dürfen, jedoch unter strikter Einhaltung der Vertraulichkeit.
- Bei Studienergebnissen, die direkt meine Gesundheit betreffen, werde ich informiert. Wenn ich das nicht wünsche, informiere ich meinen Hausarzt.
- Ich weiss, dass meine gesundheitsbezogenen und persönlichen Daten nur in verschlüsselter Form zu Forschungszwecken für diese Studie weitergegeben werden können.

- Ich bin einverstanden, dass sich das Studienteam an meinen Hausarzt wenden kann, wenn ich einen oder mehrere nachträgliche Telefonate für die Studie nicht beantwortet.
- Im Fall einer Weiterbehandlung ausserhalb des Prüfzentrums ermächtige ich meinen nachbehandelnden Arzt, meine für die Studie relevanten Nachbehandlungsdaten dem Hausarzt zu übermitteln.
- Sollten Sie im Verlauf der Studie dennoch schwanger werden, bitten wir Sie darum, Ihren Hausarzt über eine allfällige Schwangerschaft, deren Verlauf und Ausgang genau zu informieren.
- Ich kann jederzeit und ohne Angabe von Gründen von der Studienteilnahme zurücktreten. Meine weitere medizinische Behandlung ist unabhängig von der Studienteilnahme immer gewährleistet. Die bis zum Rücktritt erhobenen Daten werden für die Auswertung zur Studie verwendet.
- Ich bin einverstanden, dass meine anonymisierten Daten für andere Untersuchungen zu einem späteren Zeitpunkt weiterverwendet werden. Ich erlaube, dass meine anonymisierten Daten später an eine andere Datenbank in der Schweiz oder im Ausland für noch nicht näher definierte Untersuchungen (Weiterverwendung) verschlüsselt versandt und verwendet werden kann. Diese andere Datenbank muss die gleichen Standards einhalten wie die Datenbank zu dieser Studie.
- Ich bin darüber informiert, dass eine Versicherung Schäden deckt, die auf die Studie zurückzuführen sind.
- Ich bin mir bewusst, dass die in der Teilnehmerinformation genannten Pflichten einzuhalten sind. Im Interesse meiner Gesundheit kann mich der Hausarzt jederzeit von der Studie ausschliessen.

Ort, Datum	Unterschrift Teilnehmerin/Teilnehmer

Bestätigung des Hausarztes/der Prüfperson: Hiermit bestätige ich, dass ich dieser Teilnehmerin/ diesem Teilnehmer Wesen, Bedeutung und Tragweite der Studie erläutert habe. Ich versichere, alle im Zusammenhang mit dieser Studie stehenden Verpflichtungen gemäss dem geltenden Recht zu erfüllen. Sollte ich zu irgendeinem Zeitpunkt während der Durchführung der Studie von Aspekten erfahren, welche die Bereitschaft der Teilnehmerin/ des Teilnehmers zur Teilnahme an der Studie beeinflussen könnten, werde ich sie/ ihn umgehend darüber informieren.

Ort, Datum	Name und Vorname der Hausärztin/ des Hausarztes in Druckbuchstaben<br><br>Unterschrift der Hausärztin/des Hausarztes
