# Supplementary material for: Oral corticosteroids for post-infectious cough in adults: study protocol for a double-blind randomized placebo-controlled trial in Swiss family practices (OSPIC trial)
Source: Trials. 2020 Nov 23;21:949. doi: 10.1186/s13063-020-04848-4 (PMC7681763; doi:10.1186/s13063-020-04848-4)
Supplement: Supplementary file 2 — Additional file 2. Copy of the original funding documentation, Swiss National Science Foundation (SNSF), Investigator Initiated Clinical Trials grant (IICT 2018 call, 33IC30_179657 3) [file 13063_2020_4848_MOESM2_ESM.docx]

# Part I: Study-specific Monitoring Plan

## Study Details

| **Study Title** | Oral corticosteroids for post-infectious cough in adults: A double-blind randomised placebo-controlled trial in Swiss family practices  **(OSPIC TRIAL)** |
| --- | --- |
| Product Name, if available | Prednison |
| Study Protocol Version and Date | Version 2.1, 29-Jan-2020 |
| Dossier ID of service provider | ex16Zeller |
| Sponsor Study Code | **OSPIC** |

| **Sponsor** | Universitäres Zentrum für Hausarztmedizin Beider Basel |
| --- | --- |
| Contact Person  (Title, First name, Name, Function) | Prof. Dr. med. Andreas Zeller |
| Address | Rheinstrasse 26, 4410 Liestal |
| Tel., Fax, e-mail | + 41 (0) 61 925 20 75  [andreas.zeller@unibas.ch](mailto:andreas.zeller@unibas.ch) |

| **Contact Person for Monitoring** | DKF/CTU-Universitätsspital Basel |
| --- | --- |
| Contact Person  (Title, First name, Name, Function) | Klaus Ehrlich  Head of Monitoring-Group |
| Address | Schanzenstrasse 55, 4031 Basel |
| Tel., Fax, e-mail | +41 61 556 56 26  +41 61 265 94 10  [klaus.ehrlich@usb.ch](mailto:klaus.ehrlich@usb.ch) |

## Approval Details

| **Clinical Monitoring Plan Approval** | |
| --- | --- |
| Version/ Date of Monitoring Plan | Version 0.3, 13-Oct-2020 |
| Author of Monitoring Plan | Petra Forst |
| Reviewer of Monitoring Plan | Klaus Ehrlich |

| **Service Provider** | |
| --- | --- |
| Contact Name: | Klaus Ehrlich |
| Contact Function: | Head of Monitoring-Group |

| **Sponsor** | |
| --- | --- |
| Sponsor Contact Name: | Prof. Dr. med. Andreas Zeller |
| Sponsor Contact Function: | Sponsor-Investigator |

## General Information

The Monitoring Plan consists of two parts. General monitoring terms are listed in part II (Attachment to Monitoring Plan). In part I -in the actual Monitoring Plan- study specific conditions are regulated.

Study specific agreements overrule standard statements of the attachment.

This Monitoring Plan is based on the clinical study-specific requirements and the agreed upon commitments between

Universitäres Zentrum für Hausarztmedizin Beider Basel

Rheinstrasse 26

4410 Liestal

and

DKF/CTU-Universitätsspital Basel

Schanzenstrasse 55

CH-4031 Basel

Switzerland

For further details see contract of 06^th^ November 2019.

If agreed commitments change after study start, the contract will be amended and if necessary a new version of Monitoring Plan will be issued. Service provider will act as Monitor on behalf of Sponsor for this study.

Monitor (administrative) and Principal Investigator (medical questions) are the first line of contact for study site.

## Study Design

### Study sites

40 practitioners located in 28 sites in the Switzerland will participate in this study. Each practitioner committed to enrol 10 patients. 16 practitioners will be monitored once.

The first 8 practitioner who have enrolled their first 3 subjects, which have completed day 14 (primary endpoint) will be monitored. After last patient out, additional 8 practitioners with the highest numbers of completed subjects (day 14) and who have not been monitored before will be monitored too.

This Monitoring Plan is valid for the above-mentioned sites, based on the participants list dated
10-Nov-2019. If new sites are added to the study, an amendment to this plan will be written.
For contact details see topic 8.

### Summary

This is a prospective, multicentre, randomized, placebo-controlled Study in patients with subacute, post-infectious cough. It will be examined, how a 5-day treatment with prednisone p.o. will influence the cough-associated quality of life of the patients positively.

- **Primary endpoint***
  - Cough-related QoL assessed by the LCQ score 14 days after randomisation
- **Secondary endpoints***

• Cough-related QoL assessed by the LCQ score at 7 and 28 days and at 3 months after randomisation

• Cough-related QoL sub-domains physical, psychological, and social at 7 and 28 days and at 3 months after randomisation

• Overall cessation of cough 7, 14, 28 days and 3 months after randomization

• Incidence rate of re-consultations with the treating GP and/or hospitalisations within 3 months following randomisation

• Total AE within 3 months after randomization

• SAE within 3 months after randomization.

- **Inclusion criteria***

• Age ≥ 18 years

• Patients seeing a GP for a dry or productive post-infectious cough (3 to 8 weeks) after URTI

• Patients able and willing to give informed consent by themselves and to fill in the LCQ on day 0 with the GP and to answer phone calls from the research staff/study nurse at day 7, 14, and 28, and at 3 months for outcome assessment

- **Exclusion criteria***

**•** Patients with hypersensitivity to prednisone or to one of the adjuvants in the drug’s composition,

• Patients with known or suspected diagnoses associated with cough, such as: pneumonia or suggestive symptoms and signs (abnormal vital signs, i.e. heart rate >100/min, respiratory rate >25/min, fever), allergic rhinitis, sinusitis, bronchial asthma, chronic pulmonary disease (COPD), or gastroesophageal reflux disease,  Patients with other chronic disease such as bronchiectasis, cystic fibrosis, cancer, tuberculosis, heart failure.

• Use of inhaled or oral corticosteroids within the last four weeks

• Immunodeficiency/immunocompromised state (e.g. cancer chemotherapy, HIV infection, administration of immune-suppressive agents)

• Pregnancy/breastfeeding, as evaluated through screening

• Regular treatment known to be associated with cough (e.g. angiotensin-converting enzyme inhibitors)

• Patients with a documented diagnosis of glaucoma or osteoporosis in the GP’s patient health record

• Experienced fractures due to osteoporosis

•Patients with uncontrolled diabetes (as deemed by GPs who appraise whether the potential side effects of short-time corticosteroids on glucose levels exceed the hypothesised benefit on cough)

* Relevant is the last approved version of the protocol or protocol amendment

### Investigational Medicinal Product (IMP) or Medical Device (MD) (N/A )

Prednisone/Placebo has been notified by Swissmedic: yes  no

As per contract defined, the Monitor will ensure during the monitoring visits:

IMP/MD accountability:  no  yes  IVRS-System

IMP/MD storage:  no  yes

Handling of Medical Product/ Medical Device:

| IMP/MD packaging | na |
| --- | --- |
| Shipment to site | na |
| Return IMP/MD address | na |
| Return packaging instructions | na |

### Study Timeline

| Monitor training (study specific) by Sponsor  no  yes |  |
| --- | --- |
| Investigator Meeting (organized by Sponsor)  no  yes |  |
| Initiation Visit(s) | Approx. Q4 2020 |
| First patient first visit | Q4 2020 |
| Last patient first visit | Approx. Q4 2021 |
| Last patient last visit (including follow up period if applicable) | Approx. Q2 2022 |
| Query resolution complete | Approx. Q2 2022 |
| Database lock | TBD |
| Close-out visit (after database lock) | Approx. Q3 2022 |

### Extend of Monitoring based on risk-adapted approach

Details and amount of monitoring are in general defined by the risk category according to the Human Research Act and confirmed by the SCTO-“Monitoring Risk Analysis”. Further criteria (such as experience of a site, use of a validated database, function as the coordinating site) could increase or decrease the extend of monitoring for a study.

| HRA risk category | B |
| --- | --- |
| Risk-adapted Monitoring Class  according to SCTO Monitoring Risk Analysis | Mid risk |
| Risk score  based on evaluation of further criteria | na |

| Recommended extend of monitoring  Based on above documented risk analysis | • Site Initiation Visit  • 2 Routine monitoring visits  • Close Out Visit |
| --- | --- |
| Contracted extend of monitoring | • 2 Routine monitoring visits |
| Explanation in case of discrepancy between recommendation and contract | Site Initiation and Close Out Visits will be done by the Sponsor team |

### Monitoring Timeline

| **Study Period** | **Schedules** | **Hours on site/ in-house** |
| --- | --- | --- |
| Site initiation visit | Prior to inclusion of the first patient | Done by the Sponsor team |
| Monitoring visit 1 | First 8 practitioners with 3 enrolled subjects, who have completed day 14 (primary endpoint): | 12h including preparation and reporting |
| Monitoring visit 2 | After last patient out of the study, the 8 practitioners with the highest number of completed subjects (day14) and not monitored before.  Limit for monitoring will be 10 subjects per practitioner: | 12h including preparation and reporting |
| Close-out visit | After database lock | Done by the Sponsor team |
| Central monitoring / online monitoring | non | na |
| Regular communication agreed? | non | na |
| Additional comments | non | |

### Scope of Monitoring

| **Action** | **Extent (%)** | **Comments** |
| --- | --- | --- |
| Informed consent | 100% | At monitored practitioners |
| Inclusion/exclusion | 100% | At monitored practitioners |
| Primary Endpoint | 100% | At Study Nurses CTU Basel |
| Secondary Endpoint | non | na |
| Safety (AE/SAE) | «see comment» | First documented SAE at monitored practitioner |
| TMF/ISF | «see comments» | Trial Master File (TMF) binders with content list and labelled dividers will be compiled by the CTU.  Investigator Site Files (ISFs) will be prepared by the sponsor/study team.  During the routine monitoring visits only the Delegation Log and the Subject-ID-Log will be used as reference documents from the ISF.  Monitored practitioner will be checked, if «GCP-Video-Training» has been done/documented on the training log before the first subject has been enrolled.  Visit Log will be completed during the visit |
| Sample storage & logistics | na | - |
| Device/Disposable accountability | na | - |
| Data set | 100% | 100 % SDV in secuTrial for the following data points:  **Baseline:**  - In/Exclusion Criteria based on medical records and OSPIC GP questionnaire  - QoL questionnaire subject  **Day 7:** - Date, visit window - One SAE, if occured  **Day 14:**  - Date, visit window - One SAE, if not occurred until day 7  -QoL questionnaire subject |

The Monitor must notify line management and Sponsor if additional time or resources are necessary.

## Documentation and Communication of Protocol Deviations

A protocol violation or deviation is any failure to comply with the protocol. If applicable the following protocol violations/deviations (as well as the necessary corrective actions) need to be reported in the Monitoring Report and discussed with Investigator.

In case of severe violations line management and Sponsor must be informed immediately during the same visit:

- Informed consent process not adequately performed
- Violation of Inclusion/Exclusion criteria
- Failure to comply with IMP storage, dispensation, use or return requirements
- IMP misallocation
- Use of prohibited concomitant medications
- Serious Adverse Event & Adverse Event reporting requirements not followed
- Subject follow ups and visits not performed correctly as described in the protocol
- Required tests and sampling procedures incorrectly or not performed
- Any GCP non-compliance

All deviations/violations must be reported by monitor in follow-up letters & monitoring visit reports. The Monitor must confirm that the site has properly notified Sponsor and Independent Ethics Committee (IEC).

If applicable the sponsor will inform Competent Authority (Swissmedic).

## Safety Reporting (if applicable)

### Adverse Event (AE)

Adverse Events (AE) must be documented according to the valid version of the GCP Basisprozess “Visitenplanung, Erfassung von Adverse Events und Drug/Device Accountability”. If an AE is continuing, the Monitor will follow it up at the next visit and each subsequent visit, as applicable, until the AE is resolved.

### Serious Adverse Event (SAE)

Serious Adverse Events must be reported immediately (within 24 hours after detection) to Sponsor. Independent Ethic Committee and Competent Authority have to be informed according to the valid version of the GCP Basisprozess “Meldung von unerwünschten Ereignissen (AEs, SAEs und SUSARs)”.

Source document verification of the information captured on the SAE form has to be performed during the next monitoring visit at the site.

If the Monitor identifies a SAE during the monitoring visit, which has not been previously reported, the Monitor will request the site to complete a SAE form and to inform Sponsor and Independent Ethics Committee as per legally defined timelines.

Investigator and Sponsor have to be informed by the monitor accordingly and have to be advised that they must comply with all reporting requirements as described in the valid version of the GCP Basisprozess “Meldung von unerwünschten Ereignissen (AEs, SAEs und SUSARs)”.

### Pregnancy

A Pregnancy has to be handled like a SAE. SAE forms or if used Pregnancy report templates, should be filed in the TMF/ISF.

## Audit/ Inspection

If any of the parties involved in the study becomes aware that an audit/ inspection by a competent Authority or others is planned, all other parties have to be informed immediately in order to prepare for the visit.

## Contact Details

### Sponsor-Investigator

| Site Name & No. | Universitäres Zentrum für Hausarztmedizin Beider Basel |
| --- | --- |
| Contact Person | **Prof. Dr. med. Andreas Zeller** |
| Function | Sponsor-Investigator |
| Address | Rheinstrasse 26, CH-4410 Liestal |
| Tel., Fax, e-mail | Tel.: +41 61 925 20 75+41 61 265 73 44+41 61 265 73 44  E-Mail: [andreas.zeller@unibas.ch](mailto:andreas.zeller@unibas.ch) |

### Study Sites

| Site Name & No. | 40 Hausärzte (practitioner) in 28 Praxen (sites) gemäss Teilnehmerliste vom 10. Nov. 2019 |
| --- | --- |
| Contact Person |  |
| Function |  |
| Address |  |
| Tel., Fax, e-mail |  |

### Pharmacy

| Name | Universitätsspital Basel, Spital-Pharmazie |
| --- | --- |
| Contact Person | Anne Henn |
| Function | Studienkoordinatorin |
| Address |  |
| Tel., Fax, e-mail | Email: [Anne.henn@usb.ch](mailto:Anne.henn@usb.ch) |

### Ambulatens Studienzentrum (ASZ)

| Name | Unversitätsspital Basel, DKF |
| --- | --- |
| Contact Person | Silke Purschke |
| Function | Leitende Study Nurse |
| Address | Schanzenstrasse 55, 4031 Basel |
| Tel., Fax, e-mail | + 41 61 32 87709, email: [silke.purschke@usb.ch](mailto:silke.purschke@usb.ch) |

### Ambulantes Studienzentrum (ASZ)

| Name | Unversitätsspital Basel, DKF |
| --- | --- |
| Contact Person | Vanessa Grassedonio |
| Function | Senior Study Nurse |
| Address | Schanzenstrasse 55, 4031 Basel |
| Tel., Fax, e-mail | + 41 61 32 85541, email: [vanessa.grassedonio@usb.ch](mailto:vanessa.grassedonio@usb.ch) |

### Monitoring

| Name | Unversitätsspital Basel, DKF |
| --- | --- |
| Contact Person | Petra Forst |
| Funktion | Monitor |
| Address | Schanzenstrasse 55, 4031 Basel |
| Tel., Fax, e-mail | + 41 61 55 65625, email: [petra.forst@usb.ch](mailto:petra.forst@usb.ch) |

### Data Management

| Name | Unversitätsspital Basel, DKF |
| --- | --- |
|  | Dr. Nicole Bruni |
| Function | Data Manager |
| Address | Spitalstrasse 12, 4031 Basel |
| Tel., Fax, e-mail | + 41 61 [32 85413](mailto:32%2085413), email: [constantin.sluka@usb.ch](mailto:constantin.sluka@usb.ch) |

### Statistics

| Name | Unversitätsspital Basel, DKF |
| --- | --- |
| Contact Person | Dr. phil. nat. Tobias Erlanger |
| Function | Statistiker |
| Address | Spitalstrasse 12, 4031 Basel |
| Tel., Fax, e-mail | + 41 61 [32 85414](mailto:32%2085414), email: [tobias.erlanger@usb.ch](mailto:tobias.erlanger@usb.ch) |

# Part II: Attachment to Monitoring Plan

## Introduction

## Applicable Standard Operating Procedures (SOP)

All Monitoring Visits will be conducted in accordance with the below listed SOPs, if not otherwise contracted.

| **Category** | **Title** |
| --- | --- |
| GCP-Basisprozess | Anlegen des Trial Master File (TMF) |
| GCP-Basisprozess | Anlegen des Investigator Site File (ISF) |
| GCP-Basisprozess | Erstellung des Monitoringplans und Durchführung der Initiierungsvisite |
| GCP-Basisprozess | Patientenscreening und –einschluss |
| GCP-Basisprozess | Aufklärung des Patienten und Einholen der Einwilligung |
| GCP-Basisprozess | Visitenplanung, Erfassung von Adverse Events und Drug/Device Accountability |
| GCP-Basisprozess | Meldung von unerwünschten Ereignissen (AEs, SAEs und SUSARs) |
| GCP-Basisprozess | Durchführung einer Monitoringvisite |
| GCP-Basisprozess | Durchführung der Abschlussvisite (Close-out Visite) |
| GCP-Basisprozess | Meldung bei Abschluss, Abbruch oder Unterbruch der Studie |
| GCP-Basisprozess | Archivierung |

### Monitoring Training

Compliance with protocol requirements is important to ensure ethical conduct of clinical trial, the scientific validity, and accuracy and completeness of data produced during the clinical study. Monitoring helps to ensure compliance.

In particular, the monitor(s) have to be trained in:

- Any general monitoring requirements, e.g. protocol, Case Report Form (CRF), Source Data Verification (SDV)
- GCP Basisprozesse
- Regulations and Guidelines in the actual valid version
- International Conference on Harmonization – Good Clinical Practice
  - Humanforschungsgesetz (HFG)
  - Verordnung über klinische Versuche KlinV
  - Medizinprodukteverordnung (MepV)

As employer of the initially assigned monitor the service provider will be responsible for study specific training/ handover from assigned Monitor to any Monitor later joining the project.

### Study Specific Training

Sponsor will be responsible for study specific training. Training has to be documented in a signed study training log which will be filed in the Trial Master File (TMF)/ Investigator site File (ISF). A copy of all training related presentations and further information has to be filed as well.

The training should cover at least:

- Medical background and endpoint training
- Study design
- Study timelines
- Logistics (shipment, blood samples, drug supply, etc.)
- (e)Case Report Form (CRF) composition, completion and shipment (in case of paper version)
- Query-processing/ Data base lock

Study specific training is basically meant for the site. In addition, Monitor should be trained by the sponsor with respect to monitoring issues.

## Course of Events

### Pre-study Activities

All pre-study activities are normally performed by sponsor (site selection, site feasibility, regulatory). If a service provider (e.g. a CTU) is involved in pre-study activities, this has to be specified in the contract.

### Correspondence

All relevant study related correspondence (between sponsor/ site/ service provider and external parties) by letter, e-mail or telephone should be documented and filed into TMF/ ISF. If sponsor requests a list for contacts, this must be contracted.

### Confirmation of Visit

All visits will be preceded by a confirmation letter including agenda (date, time, place of visit, requested documents and source data attendees, etc.) sent to the investigator (and other staff, if applicable).

It is acceptable to fax or e-mail the confirmation letter.

### Initiation Visit

If determined per contract, the site will be initiated. According to GCP the site initiation visit has to be performed after study approval but prior to subject enrolment.

During the initiation visit the Monitor can be supported by other team members that are delegated by the sponsor.

The following topics are taken into account during study initiation:

- Set up **personal contacts** with site staff
- **Train** whole study team focussed on study specific procedures, safety and regulatory issues (i.e. protocol, Investigator’s brochure (IB), responsibilities, timelines, study procedures, monitoring procedures, administrations, protocol deviations and consequences)
- Inform about the requirement to document critical findings (e.g. on a Note to File)
- Discuss **GCP** and remind on regulatory requirements
- Discuss anticipated **schedule of patient visits** and which activities will occur at each visit (routine work/ additional work for the study)
- Review **logistics** (CRF, IMP/MD, material, technics, etc.). Assure that site received all necessary study supplies and is trained about supply forms and handling
- If applicable, check availability, storage and limited access to **Investigational Medical Product** (IMP)/ **Medical Device** (DM) in compliance with study specific requirements
- If applicable, review and explain **record keeping** (drug accountability) per site and subject
- If applicable, review **facilities** for IMP/ MD storage, local laboratory and examination rooms (ECG, spirometry, etc.)
- Check availability of appropriate equipment (freezers, centrifuge, ECG, etc.)
- Review laboratory sampling procedures and documentation as specified in the protocol (laboratory manual, reports etc.)
- Review and explain **TMF/ ISF completeness** (e.g. IB, signed clinical protocol, etc.) and availability of all required regulatory documents with responsible site personnel. Monitor will provide instructions to site personnel on organization and maintenance of documents in TMF/ISF
- Ensure authorisation of study staff by investigator (log of functions and responsibilities)
- Instruct site personnel in **Informed Consent process** and requirements
- Review and explain **AE/ SAE/ pregnancy reporting** procedures and documentation
- Remind Investigator’s **reporting responsibilities** to Independent Ethics Committee/ Authorities/Sponsor (safety, annual report, closure of site)
- Review **CRF and completion procedures**
- Review source documentation and **Source Data Verification** (SDV)
- Discuss **Data Clarification Form** (DCF) procedure and Data management process
- Ensure **Site Visit Log** is signed

Once the site initiation visit is completed and all necessary material is available, the site will be authorized by the sponsor to start enrolment (“green light”-process).

After the visit Monitor will write a visit report and a follow-up letter/e-mail.

In case that the monitor is involved into the study after enrolment has already started, an official study initiation visit cannot be performed. However, topics that are usually discussed during study initiation will be dealt with in the first contracted monitoring visit.

### Interim Monitoring Visit

Monitoring visits will be conducted in order to help the site following all study requirements as specified in the protocol and in ICH/GCP and to assist with anything needed to ensure the rights, wellbeing and safety of the subjects as well as to establish good data quality. Additional persons (co-monitor, designee of sponsor, EC or authority) may support the Monitor during the visit.

During the Monitoring Visit the Monitor will:

- Check whether **changes in responsibilities of site personnel** have occurred
- If necessary, train new site personnel on the protocol and study procedures
- Confirm that **site facility** remains adequate for performing the study
- Assure that a site has sufficient **study supplies**
- Check and confirm that site is compliant with **patient assessments** as per protocol
- Check and confirm **adherence to protocol** and document protocol violations/deviations in a Note to File (NTF). Monitor must communicate violations/deviations to the Investigator/ sponsor
- Review **enrolment** including number of subjects screened, enrolled, completed and withdrawn. If necessary, discuss options to increase patient recruitment.
- Check that there is a correctly signed **Informed Consent Form** (ICF) for each patient in the approved version. Remind site to hand over a copy of the signed ICF to patients.
- Check that withdrawals are appropriately documented including the reasons for **withdrawal**
- Verify that subjects meet **inclusion and exclusion criteria** by comparing with health records as defined in the scope of monitoring (Section 4.7; Part I)
- If applicable, perform **IMP/ MD accountability** per site/ patient
- Verify that there is no change in normal **laboratory values** for local laboratory safety parameters, replace version if needed
- Review **biological samples** storage and shipment
- Review **AE/ SAE/ pregnancies** documentation and reporting and note any serious and/or unexpected adverse effects, including all follow-up actions taken. Ensure that for each SAE a complete SAE report has been submitted to the responsible persons/ authorities as specified in the GCP Basisprozess “Meldung von unerwünschten Ereignissen (AEs, SAEs und SUSARs)”.
- Perform **source data verification** (SDV) on CRF data including queries in order to assess subject compliance, safety, and integrity of data
- Check **query resolution** by site and verify source data for query resolution
- **Collect** completed and verified **CRFs and DCFs**
- Review TMF/ **ISF completeness** as defined in the scope of monitoring (Section 4.7; Part I)
- Identify **action items** for site personnel and/or Monitor
- **Discuss findings with investigator/ site staff**
- Record visit in **site visit log**

All ongoing issues from the last Monitoring visit should be reviewed and resolved, if possible.

After the visit Monitor will write a visit report and a follow-up letter.

### Source Data Verification (SDV)

The Monitor verifies CRF data by comparing with corresponding source data (medical charts -paper or online- data or source documents including e.g.: laboratory reports, print outs of electronic data, etc.).

The Monitor:

- Ensures that discrepancies are documented in the appropriate comments section of the monitoring visit report.
  Same applies if a subject has completed participation, but site has not completed the CRF: also in this case the discrepancy has to be documented and the site is instructed to complete the CRF.
- Confirms that AEs, concomitant medications and concurrent illnesses have been entered into the appropriate sections and that cross-references to AEs and concomitant medications are correct.
- Confirms that missed patient visits, tests or examinations are adequately documented in CRF.
- If agreed with sponsor, paper CRFs respectively print-outs of eCRFs have to be collected. Collection and transmission of paper CRFs have to be documented, e.g. in a document transmission form.
  Completed electronic CRF are frozen after validation. Print-outs are stored at site.
- Performs administrative review regarding internal logic, consistency and completeness of data.

For any scans and X-rays taken during the study, the Monitor must review the associated report for source data verification.

Screening failure subjects must be listed in a log. For these subjects only ICF and reason for failure will be verified/ monitored against source data (In-/ Exclusion Criteria). No data will be recorded in CRF.

All unused paper-CRFs will be returned to Sponsor or destroyed at site (decision of Sponsor). Any destruction has to be documented; documentation will be filed in TMF/ ISF.

### Data Clarification Form (DCF)

DCFs (queries) that are distributed to site must be resolved and returned to data management (DM) within the requested timeline. In case of paper CRFs copies of the DCFs will be filed at site together with the corresponding CRFs, originals will be forwarded to DM.

The DCFs must be verified against source data and signed by PI or designee. (Signatures only if paper is used).

If any DCF is sent to DM without being previously reviewed, the Monitor will monitor the data during the next monitoring visit.

### Close-out Visit

Close-out Visit will be conducted to close the study at a site after data base lock.

During the Close-out Visit Monitor will:

- Ensure **completeness of TMF/ ISF** and update file if necessary
- Ensure that **all forms and logs** **are completed correctly** and signed by PI
- Verify that **subject identification** is complete and filed anonymously in TMF. The original is filed in ISF. For investigator-initiated studies TMF and ISF may be combined as one file and maintained at the site, but all non-anonymous documents must be filed in a separate chapter within the file.
- Ensure that all **study related documents** are on file and remind investigator of archiving requirements and retention times (e.g. ISF, Patient Data, Diaries, copies of CRF).
- Check that all **IMP/ MD are returned or destroyed** as specified in the protocol
- Check that all **randomization codes**/ blinding envelopes are returned to sponsor
- Arrange **return or destruction of any study related supply** as specified in the protocol or per decision of Sponsor
- **Inform investigator** about:
  - Responsibility for ongoing AE and SAE
  - Obligation to inform ethic committee in writing about closure of site
  - Obligation to inform Competent Authority (if involved) in writing about closure of site and end of study (Sponsor-Investigator) including final SAE information
  - Possibility of audits or inspections by authorities, ethic committees or sponsor

After the visit Monitor will write a visit report and a follow-up letter.

### Visit Report

Following each Site Visit (Initiation Visit, Interim Monitoring Visit, Close out Visit), the Monitor writes a Visit Report. All Study Visit Reports issued by the Monitor will be reviewed internally before submitting them to Sponsor for final approval and signature.

The Visit Report is submitted to Sponsor within 15 business days, internal review included.

### Follow-up Letter

After each visit the Monitor also writes a follow-up letter to the site that includes comments to the following items:

- Review of all study issues discussed with Investigator/ site staff
- Scope of monitoring
- Protocol and/or regulatory deviations identified
- Action items that need to be addressed by Principal Investigator (all action items must be directed to the PI, however he/ she can delegate to other study staff as needed)

The follow-up letter must be forwarded to the investigator within 5 business days after the monitoring visit. It is acceptable to fax or e-mail the follow up letter to the site.

### Filing of Documents

|  | **Trial Master File** | **Investigator Site File** |
| --- | --- | --- |
| Monitoring Plan | Original | ---- |
| Confirmation letters | Copy | Original |
| Pre-study visit | Original | ---- |
| Initiation Visit Report | Original | Copy |
| Interim Monitoring Visit Report | Original | ---- |
| Close-out Visit Report | Original | Copy |
| Follow-up letters | Copy | Original |
| Correspondence | Original/ Copy | Original/ Copy |
