# Supplementary material for: Oral corticosteroids for post-infectious cough in adults: study protocol for a double-blind randomized placebo-controlled trial in Swiss family practices (OSPIC trial)
Source: Trials. 2020 Nov 23;21:949. doi: 10.1186/s13063-020-04848-4 (PMC7681763; doi:10.1186/s13063-020-04848-4)
Supplement: Supplementary file 3 — Additional file 3. [file 13063_2020_4848_MOESM3_ESM.pdf]

Clinical Protocol for Investigator initiated trials (IIT)

Protocol: Interventional study with investigational medicinal product (IMP)
Clinical Study Protocol

ORAL CORTICOSTEROIDS FOR POST-INFECTIOUS COUGH IN ADULTS: A DOUBLE-BLIND RANDOMISED PLACEBO-CONTROLLED TRIAL IN SWISS FAMILY PRACTICES (OSPIC TRIAL)

ORAL CORTICOSTEROIDS FOR POST-INFECTIOUS COUGH IN ADULTS / OSPIC Trial / Orale Kortikosteroide für die Behandlung von post-infektiösem Husten bei Erwachsenen

Study Type:	Clinical trial with Investigational Medicinal Product (IMP)
Study Categorisation:	Risk category according to HRA (B)
Study Registration:	Swiss National Clinical Trials Portal (SNCTP), registration under submission<br>Clinicaltrials.gov, registration under submission
Study Identifier:	Study ID AZ-1-2019
Sponsor, Sponsor-Investigator or Principal Investigator:	Prof. Dr. med. Andreas Zeller<br>Head of the Centre for Primary Health Care (uniham-bb)<br>University of Basel<br>Kantonsspital Baselland<br>Rheinstrasse 26<br>CH-4410 Liestal<br>Tel: +41 (0)61 925 20 75<br>Email: andreas.zeller@unibas.ch
Investigational Product:	Prednisone, PREDNISON Galepharm Tabl. 20 mg (Swissmedic authorization 50821)
Protocol Version and Date:	Version 2.1/ 29.Jan.2020

CONFIDENTIAL
The information contained in this document is not confidential and is the property of Prof. Dr. med. Andreas Zeller and the Centre for Primary Health Care (uniham-bb) (or "sponsor"). The information may not - in full or in part - be reproduced or published without prior written authorisation from the sponsor except to the extent necessary to obtain informed consent from those who will participate in the study.

Signature Page(s)

Study number	Study will be registered on clinicaltrials.gov and kofam.ch
Study Title	Oral corticosteroids for post-infectious cough in adults: a double-blind randomised placebo-controlled trial in Swiss family practices (OSPIC TRIAL)

The Sponsor-Investigator and trial statistician have approved the protocol Version 2.1 dated 29.01.2020, and confirm hereby to conduct the study according to the protocol, current version of the World Medical Association Declaration of Helsinki, ICH-GCP guidelines or ISO 14155 norm if applicable and the local legally applicable requirements.

Sponsor-Investigator:

Prof. Dr. med. Andreas Zeller
Head of the Centre for Primary Health Care (uniham-bb)
University of Basel
Kantonsspital Baselland
Rheinstrasse 26
CH-4410 Liestal
Tel: +41 (0)61 925 20 75
Email: andreas.zeller@unibas.ch

Liestal/ 29.01.2020

Place/Date Signature

Trial statistician:

Dr. phil. nat. Tobias E. Erlanger

Basel / 20.11.2019

Place / Date Signature

Local Principal Investigator at study site*:

I have read and understood this trial protocol and agree to conduct the trial as set out in this study protocol, the current version of the World Medical Association Declaration of Helsinki, ICH-GCP guidelines or ISO 14155 norm and the local legally applicable requirements.

Site	Centre for Primary Health Care (uniham-bb)<br>University of Basel<br>Kantonsspital Baselland<br>Rheinstrasse 26<br>4410 Liestal
Principal investigator	Prof. Dr. med. Andreas Zeller

Basel/ 29.01.2020

Place/Date Signature

Table of Contents

STUDY SYNOPSIS **8**
ABBREVIATIONS **14**
STUDY SCHEDULE **16**
1. STUDY ADMINISTRATIVE STRUCTURE **17**
1.1 Sponsor, Sponsor-Investigator 19
1.2 Principal Investigator(s) 19
1.3 Statistician ("Biostatistician") 20
1.4 Laboratory 20
1.5 Monitoring institution 20
1.6 Data Safety Monitoring Committee 21
1.7 Any other relevant Committee, Person, Organisation, Institution 21
2. ETHICAL AND REGULATORY ASPECTS **22**
2.1 Study registration 22
2.2 Categorisation of study 22
2.3 Competent Ethics Committee (CEC) 22
2.4 Competent Authorities (CA) 22
2.5 Ethical Conduct of the Study 22
2.6 Declaration of interest 23
2.7 Patient Information and Informed Consent 23
2.8 Participant privacy and confidentiality 23
2.9 Early termination of the study 24
2.10 Protocol amendments 24
3. BACKGROUND AND RATIONALE **25**
3.1 Background and Rationale 25
3.2 Investigational Product (treatment) and Indication 26
3.3 Preclinical Evidence 26
3.4 Clinical Evidence to Date 26
3.5 Dose Rationale: Rationale for the intended purpose in study 26
3.6 Explanation for choice of comparator (or placebo) 26
3.7 Risks / Benefits 26
3.8 Justification of choice of study population 27
4. STUDY OBJECTIVES **28**
4.1 Overall Objective 28
4.2 Primary Objective 28
4.3 Secondary Objectives 28
4.4 Safety Objectives 28
5. STUDY OUTCOMES **29**
5.1 Primary Outcome 29
5.2 Secondary Outcomes 29
5.3 Other Outcomes of Interest 29
5.4 Safety Outcomes 29
• Incidence rate of re-consultations at GP and/or hospitalisations within 3 months following randomisation 29

6. **STUDY DESIGN** **30**
6.1 General study design and justification of design 30
6.2 Methods of minimising bias 31
6.2.1 Randomisation 31
6.2.2 Blinding procedures 31
6.2.3 Other methods of minimising bias 31
6.3 Unblinding Procedures (Code break) 31
7. **STUDY POPULATION** **33**
7.1 Eligibility criteria 33
7.2 Recruitment and screening 34
7.3 Assignment to study groups 34
7.4 Criteria for withdrawal / discontinuation of participants 34
8. **STUDY INTERVENTION** **36**
8.1 Identity of Investigational Products (treatment) 36
8.1.1 Experimental Intervention (treatment) 36
8.1.2 Control Intervention (standard/routine/comparator treatment) 36
8.1.3 Packaging, Labelling and Supply (re-supply) 36
8.1.4 Storage Conditions 37
8.2 Administration of experimental and control interventions 37
8.2.1 Experimental Intervention 37
8.2.2 Control Intervention 37
8.3 Dose modifications 37
8.4 Compliance with study intervention 38
8.5 Data Collection and Follow-up for withdrawn participants 38
8.6 Trial specific preventive measures 38
8.7 Concomitant Interventions (treatments) 38
8.8 Study Drug Accountability 38
8.9 Return or Destruction of Study Drug 39
9. **STUDY ASSESSMENTS** **40**
9.1 Study flow chart(s) / table of study procedures and assessments 40
9.2 Assessments of outcomes 41
9.2.1 Assessment of primary outcome 42
9.2.2 Assessment of secondary outcomes 42
9.2.3 Assessment of other outcomes of interest 42
9.2.4 Assessment of safety outcomes 42
9.2.5 Assessments in participants who prematurely stop the study 43
9.3 Procedures at each visit 43
9.3.1 Baseline Day 0, Visit 1 43
9.3.2 Day 7 (+2 days), Phone Call 1 43
9.3.3 Day 14 (+2 days), Phone Call 2 43
9.3.4 Day 28 (+2 days), Phone Call 3 43
9.3.5 Month 3 (+7 days), Phone Call 4 44
10. **SAFETY** **44**
10.1 Drug studies 44

10.1.1 Definition and assessment of (serious) adverse events and other safety related events 44
10.1.2 Reporting of serious adverse events (SAE) and other safety related events 45
10.1.3 Follow up of (Serious) Adverse Events 47
11. STATISTICAL METHODS 48
11.1 Hypothesis 48
11.2 Determination of Sample Size 48
11.3 Statistical criteria of termination of trial 48
11.4 Planned Analyses 48
11.4.1 Datasets to be analysed, analysis populations 48
11.4.2 Primary Analysis 49
11.4.3 Secondary Analyses 49
11.4.4 Interim analyses 49
11.4.5 Safety analysis 49
11.4.6 Deviation(s) from the original statistical plan 49
11.5 Handling of missing data and drop-outs 49
12. QUALITY ASSURANCE AND CONTROL 50
12.1 Data handling and record keeping / archiving 50
12.1.1 Case Report Forms 50
12.1.2 Specification of source documents 50
12.1.3 Record keeping / archiving 50
12.2 Data management 50
12.2.1 Data Management System 51
12.2.2 Data security, access and back-up 51
12.2.3 Analysis and archiving 51
12.2.4 Electronic and central data validation 51
12.3 Monitoring 51
12.4 Audits and Inspections 52
12.5 Confidentiality, Data Protection 52
12.6 Storage of biological material and related health data 52
13. PUBLICATION AND DISSEMINATION POLICY 53
14. FUNDING AND SUPPORT 54
14.1 Funding 54
14.2 Other Support 54
15. INSURANCE 54
16. REFERENCES 55
17. APPENDICES 57

STUDY SYNOPSIS

**Sponsor / Sponsor-Investigator**	Prof. Dr. med. Andreas Zeller<br>Head of the Centre for Primary Health Care (uniham-bb)
**Study Title:**	Oral corticosteroids for post-infectious cough in adults: A double-blind randomised placebo-controlled trial in Swiss family practices (OSPIC Trial)
**Short Title / Study ID:**	Oral corticosteroids for post-infectious cough in adults / Study ID AZ-1-2019
**Protocol Version and Date:**	Version 2.1/ 29.01.2020
**Trial registration:**	Swiss National Clinical Trials Portal (SNCTP), registration under submission<br>ClinicalTrials.gov, registration under submission
**Study category and Rationale**	Category B Clinical Trial with medicinal products authorized in Switzerland and not used as specified.<br>A double-blind randomised placebo-controlled trial which investigates the effectiveness of a 5-day prednisone treatment compared to a 5-day placebo treatment to treat post-infectious cough.
**Clinical Phase:**	Clinical study Phase 3
**Background and Rationale:**	Cough is one of the most common causes for seeking medical advice in general practices (1). Post-infectious cough is defined as lasting 3 to 8 weeks after an upper respiratory tract infection (URTI) (2). It can be very bothersome and disabling in daily activities and with significant impact on physical and psycho-social health, leading to impaired quality of life (QoL) (3). Recommendations for the management of post-infectious cough in primary care are scarce and incoherent (2, 4-5). We conducted a systematic review (PROSPERO ID: CRD42017059442) and meta-analysis of randomised clinical trials (RCTs) assessing the patient-relevant benefits and potential harms of available treatments (6). None of the six eligible RCTs found clear patient-relevant benefits and most had an unclear or high risk of bias (6).<br>There is strong evidence that oral corticosteroids for 5 days provide patient-relevant benefits without relevant harm for asthma or chronic obstructive pulmonary diseases (COPD) (7-9). Prednisone (tablets at a dose of 40 mg once daily for 5 to 7 days) is a well-established oral steroid for acute asthma or exacerbation of COPD.<br>Hence, the main research question of this study is whether a 5-day treatment with orally administered prednisone can improve the cough related QoL as measured by the Leicester Cough Questionnaire (LCQ) (10-13) of adult primary care patients with post-infectious cough triggered by an URTI.
**Objective(s):**	Primary objective: To investigate whether a 5-day treatment with 40 mg (2 tablets of 20 mg) orally administered prednisone improves on day 14 the cough-related QoL of patients with post-infectious cough after an URTI.<br>Secondary objectives: To evaluate the impact of oral prednisone on:<br>• Cough-related QoL at other time-points (7, 28 days, and 3 months after randomization)<br>• Three cough-related QoL (LCQ) sub-domains, i.e. physical, psychological and social<br>• Overall cessation of cough (i.e. proportion of patients without cough symptoms)<br>• Safety outcomes, i.e. re-consultations with the general physician (GP) and/or hospitalisations and total adverse (AE) and serious adverse events (SAE).

**Outcome(s):**	Primary outcome:<br>• Cough-related QoL assessed by the LCQ score 14 days after randomisation<br>To assess the impact of the treatment on patients' QoL the LCQ will be used. The LCQ comprises 19 items and takes 5 to 10 minutes to complete (10). The LCQ is a validated QoL measurement tool for non-specific cough and assesses the impact of cough on various aspects of life, including emotions, sleeping behaviour, work and relationships. It contains 19 items which are divided over 3 domains: physical (8 items), psychological (7 items) and social (4 items), with a 7-point Likert response scale (11, 12). We will use the validated German version of the original LCQ (13).<br><br>Secondary outcomes:<br>• Cough-related QoL assessed by the LCQ score at 7 and 28 days and at 3 months after randomisation<br>• Cough-related QoL sub-domains physical, psychological, and social at 7 and 28 days and at 3 months after randomisation<br>• Overall cessation of cough 7, 14, 28 days and 3 months after randomization<br>• Incidence rate of re-consultations with the treating GP and/or hospitalisations within 3 months following randomisation<br>• Total AE within 3 months after randomization<br>• SAE within 3 months after randomization.
**Study design:**	OSPIC is a 1:1 randomised, parallel-group, placebo-controlled, superiority trial with blinded patients, physicians and outcome assessors in a primary health care setting.

**Inclusion / Exclusion criteria:**	Inclusion criteria:<br>• Age ≥ 18 years<br>• Patients seeing a GP for a dry or productive post-infectious cough (3 to 8 weeks) after URTI<br>• Patients able and willing to give informed consent by themselves and to fill in the LCQ on day 0 with the GP and to answer phone calls from the research staff/study nurse at day 7, 14, and 28, and at 3 months for outcome assessment<br><br>Exclusion criteria:<br>• Patients with hypersensitivity to prednisone or to one of the adjuvants in the drug's composition,<br>• Patients with known or suspected diagnoses associated with cough, such as: pneumonia or suggestive symptoms and signs (abnormal vital signs, i.e. heart rate >100/min, respiratory rate >25/min, fever), allergic rhinitis, sinusitis, bronchial asthma, chronic pulmonary disease (COPD), or gastroesophageal reflux disease,<br>• Patients with other chronic disease such as bronchiectasis, cystic fibrosis, cancer, tuberculosis, heart failure.<br>• Use of inhaled or oral corticosteroids within the last four weeks<br>• Immunodeficiency/immunocompromised state (e.g. cancer chemotherapy, HIV infection, administration of immune-suppressive agents)<br>• Pregnancy/breastfeeding, as evaluated through screening<br>• Regular treatment known to be associated with cough (e.g. angiotensin-converting enzyme inhibitors)<br>• Patients with a documented diagnosis of glaucoma or osteoporosis in the GP's patient health record<br>• Experienced fractures due to osteoporosis<br>• Patients with uncontrolled diabetes (as deemed by GPs who appraise whether the potential side effects of short-time corticosteroids on glucose levels exceed the hypothesised benefit on cough)
**Measurements and procedures:**	Once patients are informed about the OSPIC trial (by their GP) and written informed consent is obtained (day 0), they will be screened against the full eligibility criteria. Eligible participants will be randomly allocated to prednisone or placebo treatment. We select a dose of 40mg (2 tablets of 20 mg) of prednisone once daily over 5 days. Participants will participate in follow-up phone calls at days 7, 14 and 28, and at 3 months from randomization. During each of these phone calls, study research staff will administer the LCQ questionnaire and will collect data on symptoms, compliance, other treatments and doctor's visits, and adverse-events.

**Study Product / Intervention:**	Pre-randomised, identically looking, numbered and marked medication glass jars with 5 daily doses of 40 mg (2 tablets of 20 mg) of prednisone (intervention group) or placebo (control group) are provided by GPs to participants. Study participants should take 2 tablets orally, in the morning for 5 days. Oral and written instructions on how the medication should be taken will be provided to the study participants. In the event of a missed dose, patients are instructed to continue medication intake on the next day.<br>PREDNISON Galepharm Tabl. 20 mg as well as placebo tablets are manufactured according to Good Manufacturing Practice (GMP)-guidelines. The prednisone medication is manufactured by Galepharm AG, 8700 Küsnacht (ZH) and packaged and labelled by the Hospital Pharmacy of the University Hospital Basel. The PREDNISON tablets' active substance is Prednisonum; the tablets also contain Excipiens pro compresso. Swissmedic authorization 50821,<br>The content of the placebo tablets is as follows: Lactose monohydrate 140 mg, microcristalline cellulose 68 mg, Croscarmellose sodium 5 mg, Magnesium stearate 2mg. The placebo tablets were manufactured by Apotheke Hotz, Zürichstrasse 176, CH- 8700 Küsnacht.
**Control Intervention (if applicable):**	Placebo will be used as comparator to prevent various biases and to ensure a double-blinded assessment. From an ethical point of view a placebo-controlled trial seems justified since there is no established therapy for post-infectious cough.
**Number of Participants with Rationale:**	A total of N=204 patients need to be recruited. Intervention group: N=102; Control group: N=102.
**Study Duration:**	Estimated duration for the main investigational plan: 1,5 years.
**Study Schedule:**	Month and year of first-participant-in (planned): February 2020<br>Month and year of last-participant-out (planned): July 2021

**Investigator(s):**	Prof. Dr. med. Andreas Zeller, MSc<br>Head of the Centre for Primary Health Care<br>Centre for Primary Health Care (uniham-bb)<br>University of Basel<br>Kantonsspital Baselland<br>Rheinstrasse 26<br>CH-4410 Liestal<br>Phone +41 (0)61 925 20 75<br>Email: andreas.zeller@unibas.ch<br><br>Dr. med. Christoph Merlo<br>Head of the Institute of Primary and Community Care<br>University of Lucerne<br>Schwanenplatz 7<br>CH- 6004 Lucerne<br>Phone +41 41 410 88 85<br>Email: merlo.c@bluewin.ch<br><br>Prof. Dr. med. Jörg Leuppi, PhD<br>Head of the University Clinic of Medicine<br>Kantonsspital Baselland<br>Rheinstrasse 26<br>CH-4410 Liestal<br>Phone +41 61 925 21 80<br>Email: joerg.leuppi@ksbl.ch<br><br>Dr. med. Stefan Essig, PhD<br>Head of Research, Institute of Primary and Community Care<br>University of Lucerne<br>Schwanenplatz 7<br>CH- 6004 Lucerne<br>Phone +41 41 410 88 85<br>Email: stefan.essig@iham-cc.ch

**Study Centre(s):**	The study will be managed by the Centre for Primary Health Care (uniham-bb) in Basel in collaboration with the Institute of Primary and Community Care in Luzern.<br><br>Centre for Primary Health Care<br>Kantonsspital Baselland<br>Rheinstrasse 26<br>CH-4410 Liestal<br><br>Institute of Primary and Community Care<br>University of Lucerne<br>Schwanenplatz 7<br>CH- 6004 Lucerne<br><br>Patient recruitment will take place in approximatively 46 primary care practices (GP practices) in the German speaking part of Switzerland and will continue until the sample size (N=204) is reached. GPs have been recruited through existing research networks by the Centre for Primary Health Care, University of Basel, and the Institute of Primary and Community Care, University of Lucerne, from the Northwestern and central Switzerland, as well as the canton of St. Gallen.
**Statistical Considerations:**	Sample size was estimated to detect the minimal clinically important difference (MCID) of 1.3 points. A standard deviation of 3.3 points was chosen and the sample size was calculated for a two-sample t-test with a two-sided alpha threshold of 5%. Overall, to be able to detect an MCID of 1.3 points with a power of 80%, a total of N=204 patients need to be recruited. We expect a drop-out rate of 5-10% (14, 15).<br><br>Analysis of the primary outcome will follow the intention-to-treat principle (ITT). It will be based on the full analysis set (FAS) which includes all patients who were randomly allocated to one of the study arms. We will test if there is a statistically significant difference of the LCQ score between the intervention and control group on alpha level of 5%.<br><br>Analysis of secondary outcomes will be of exploratory nature and results will be interpreted for hypothesis generation. For safety analyses, total number and percentages for incidence rate of re-consultations at GP and/or hospitalisations within 3 months following randomisation will be calculated. Total AE and SAE stratified by WHO-UMC (16) causality categories within 3 months after randomisation will be presented as well. Missing data will be imputed for variables required to test the hypothesis. We assume that for patients who will complete the study only a few data will be missing (17). The reason for the missing data and whether it might be at-random or not, will be examined according to guidelines (18).
**GCP Statement:**	This study will be conducted in compliance with the protocol, the current version of the Declaration of Helsinki, the ICH-GCP or ISO EN 14155 (as far as applicable) as well as all national legal and regulatory requirements.

ABBREVIATIONS

AE	Adverse Event
ASR	Annual Safety Report
BASEC	Business Administration System for Ethical Committees, (https://submissions.swissethics.ch/en/)
CA	Competent Authority (e.g. Swissmedic)
CEC	Competent Ethics Committee
CI	Confidence Interval
COPD	Chronic Obstructive Pulmonary Disease
CRF	Case Report Form
ClinO	Ordinance on Clinical Trials in Human Research *(in German: KlinV, in French: OClin, in Italian: OSRUm)*
eCRF	Electronic Case Report Form
CTCAE	Common terminology criteria for adverse events
CTU	Clinical Trial Unit
DAC	Data Access Committee
DKF	Departement Klinische Forschung (Department of Clinical Research)
DMP	Data Management Plan
EKNZ	Ethikkommission Nordwest- und Zentralschweiz (Ethics Committee for North-West and Central Switzerland)
EMA	European Medicines Agency
FAS	Full Analysis Set
GCP	Good Clinical Practice
GMP	Good Manufacturing Practice
GP	General Physician
IB	Investigator's Brochure
Ho	Null hypothesis
H1	Alternative hypothesis
HFG	Humanforschungsgesetz (Law on human research)
HIV	Human Immunodeficiency Virus
HMG	Heilmittelgesetz (Therapeutic Products Act)
HRA	Federal Act on Research involving Human Beings *(in German: HFG, in French: LRH, in Italian: LRUm)*
ICC	Intra-cluster correlation
ICH-GCP	International Conference on Harmonisation – Good Clinical Practice
IMP	Investigational Medicinal Product
IPC	Intra-patient correlation
IIT	Investigator-initiated Trial
ISF	Investigator Site File
ITT	Intention to treat

KlinV	Verordnung über klinische Versuche in der Humanforschung *(in English: ClinO, in French OClin)*
KSBL	Kantonsspital Baselland
LCQ	Leicester Cough Questionnaire
LPTh	Loi sur les produits thérapeutiques
LRH	Loi fédérale relative à la recherche sur l'être humain
MCID	Minimal Clinically Important Difference
MD	Medical Device
MedDO	Medical Device Ordinance *(in German: MepV, in French: ODim)*
MICE	Multiple Imputation by Chained Equations
OClin	Ordonnance sur les essais cliniques dans le cadre de la recherche sur l'être humain *(in German : KlinV, in English : ClinO)*
PI	Principal Investigator
PPS	Per-protocol-set
RAP	Report and Analysis Plan
RCT	Randomised Clinical Trial
RMV	Routine monitoring visits
SAE	Serious Adverse Event
SD	Standard Deviation
SAP	Statistical Analysis Plan
SNCTP	Swiss National Clinical Trials Portal
SNSF	Swiss National Science Foundation
SOP	Standard Operating Procedure
SSGIM	Swiss Society of General Internal Medicine
SUSAR	Suspected Unexpected Serious Adverse Reaction
TMF	Trial Master File
QoL	Quality of Life
URTI	Upper Respiratory Tract Infection
WHO-UMC	World Health Organization Uppsala Monitoring Centre

STUDY SCHEDULE

	STUDY PERIOD						
	Enrolment	Randomisation	Treatment	Follow-up ($T_{days/months}$)			
TIME POINT	$T_{baseline}$	T_0	$T_{days\ 1\text{-}5}$	T_{day7}	T_{day14}	T_{day28}	$T_{3months}$
**ENROLMENT**							
Eligibility	X						
Informed consent	X						
Allocation		X					
**INTERVENTION**			X				
**ASSESSMENTS**							
Baseline characteristics		X					
LCQ		X		X	X	X	X
Adherence to treatment				X			
Cessation of cough				X	X	X	X
Re-Consultations*				X	X	X	X
Hospitalisations				X	X	X	X
Concomitant treatments		X		X	X	X	X
Adverse events				X	X	X	X
Serious adverse events				X	X	X	X

* Clinical follow-up visits with the GP are at the discretion of the treating GP and/or based on patient's needs

1. STUDY ADMINISTRATIVE STRUCTURE

The Department of Clinical Research of the University Hospital Basel (DKF) will carry study monitoring and onsite management, and will act as an independent Data Access Committee (DAC). Monitoring will be carried out according to the Standard Operating Procedures (SOPs) of the Clinical Trial Unit (CTU) and following the monitoring plan agreed upon with the Sponsor. The DKF will be responsible with the setup and maintenance of the data management plan (DMP) and will store the data at time of publication on secure servers, maintained and backed up by the IT-Department of the University Hospital Basel.

The DKF is also charged with providing statistical services: design of a statistical analysis plan (SAP) approved by the Principal Investigator, perform data analysis based on the SAP and contribute to scientific publication. Clinical data processing will follow the CTU guidelines, including for aspects concerning ethics and data coding.

Clinical Trial Unit (CTU)

Department of Clinical Research

c/o University Hospital Basel

Spitalstrasse 12

CH- 4031 Basel

It is not intended to form additional committees for this trial. General practitioners (GPs) will be connected directly with the research team located at the Kantonsspital Baselland. Adverse events will be reported to the research team as well as to local authorities as described in section 10. Safety.

Forty-six GP practices have confirmed in writing their support for the OSPIC Trial. A list of the collaborating GPs and family practices is provided in the Annex.

Study data will be collected by paper CRF at the GPs' practices and delivered to the CTU where it will undergo an automated read-out into an eCRF. All eCRFs will be then captured via an electronic system (secuTrial® database) based at the IT-Department of the University Hospital Basel. The secuTrial® will be used by authorized investigators only. The eCRF will be implemented by the Data management group at the CTU of the University Hospital Basel. The secuTrial® runs on a server maintained by the IT-Department of the University Hospital Basel.

Sponsor/PI:

Prof. Dr. med. Andreas Zeller

Head of the Centre for Primary Health Care

Centre for Primary Health Care (uniham-bb)

University of Basel

Kantonsspital Baselland

Rheinstrasse 26

CH-4410 Liestal

Phone +41 (0)61 925 20 75

Email: andreas.zeller@unibas.ch

Overall responsibility for the project

Study coordinator:

Dr. sc. med. Oana Brancati-Badarau

Research Associate

Centre for Primary Health Care (uniham-bb)

University of Basel

Kantonsspital Baselland

Rheinstrasse 26
CH-4410 Liestal
Phone +49 (0)176 23 92 89 13
Email: oana.brancati-badarau@unibas.ch
Study coordination

Statistician:

Dr. phil. nat. Tobias E. Erlanger
Statistician
Clinical Trial Unit (CTU)
Department of Clinical Research
University Hospital Basel
Spitalstrasse 12
CH- 4031 Basel
Phone +41 61 328 54 14
Email: tobias.erlanger@usb.ch
Statistical support

Contact person 1 Department of Clinical Research:

PD Dr. med. Lars G. Hemkens
Senior Clinical Epidemiologist
Department of Clinical Research
University Hospital Basel
Spitalstrasse 12
CH- 4031 Basel
Phone +41 61 265 34 07
Email: lars.hemkens@usb.ch
Support in terms of study design and methodology

Contact person 2 Department of Clinical Research:

Dr. phil. nat. Constantin Sluka
Data manager
Clinical Trial Unit (CTU)
Department of Clinical Research
University Hospital Basel
Spitalstrasse 12
CH- 4031 Basel
Phone +41 61 328 54 13
Email: constantin.alexander.sluka@usb.ch
Support in terms of study design and methodology

1.1 Sponsor, Sponsor-Investigator

Prof. Dr. med. Andreas Zeller

Head of the Centre for Primary Health Care

Centre for Primary Health Care (uniham-bb)

University of Basel

Kantonsspital Baselland

Rheinstrasse 26

CH-4410 Liestal

Phone +41 (0)61 925 20 75

Email: andreas.zeller@unibas.ch

The study protocol is written by Prof. Dr. med. Andreas Zeller with input from members of the research groups of the Centre for Primary Health Care, Basel and collaborating research partners at the Institute of Primary and Community Care, Lucerne, and the Department of Clinical Research, University Hospital Basel. This is an investigator driven study and will be conducted under the supervision of Prof. Dr. med. Andreas Zeller, Prof. Dr. Jörg Daniel Leuppi and Dr. Stefan Essig. Prof. Dr. Andreas Zeller is the Principal Investigator for the study and the main responsible for the entire project. Prof. Dr. Leuppi and Dr. Essig are also responsible with overseeing the conduct of the study.

Participant data will be collected by GPs on paper based CRFs developed by the Principal Investigator in collaboration with Dr. Essig, Dr. Merlo and Prof. Dr. Leuppi. The CTU at the University Hospital Basel will provide an electronic data capture solution (secuTrial® database) for the automated read-out and storage of the participant CRFs, and sub-sequent formatting and merging of these data with participant telephone interview data.

The CTU Basel is responsible with the development, testing and deployment of the Clinical Data Management Application (CDMA) and with the preparation and implementation of a Data Management Plan (DMP). Prof. Dr. med. Andreas Zeller will review drafts and approve a final version of the CDMA and of the Data Management Plan DMP as developed by the CTU Basel. A Report and Analysis Plan (RAP) will be developed by the CTU and reviewed and confirmed by the Principal Investigator.

Data will be analysed following the RAP and in collaboration with statisticians from the Department of Clinical Research Basel, in accordance with the agreement between the Sponsor and the Department of Clinical Research Basel. Prof. Dr. med. Andreas Zeller will be involved in every step connected to this study including responsible for project development and implementation, obtaining the collaboration of general practices for recruitment and enrolment of participants, interpretation of data, writing of scientific papers and study reports etc.

1.2 Principal Investigator(s)

Prof. Dr. med. Andreas Zeller, MSc

Head of the Centre for Primary Health Care

Centre for Primary Health Care (uniham-bb)

University of Basel

Kantonsspital Baselland

Rheinstrasse 26

CH-4410 Liestal

Phone +41 (0)61 925 20 75

Email: andreas.zeller@unibas.ch

Dr. med. Christoph Merlo
Head of the Institute of Primary and Community Care
University of Lucerne
Schwanenplatz 7
CH- 6004 Lucerne
Phone +41 41 410 88 85
Email: merlo.c@bluewin.ch

Prof. Dr. med. Jörg Leuppi, PhD
Head of the University Clinic of Medicine
Kantonsspital Baselland
Rheinstrasse 26
CH-4410 Liestal
Phone +41 61 925 21 80
Email: joerg.leuppi@ksbl.ch

Dr. med. Stefan Essig, PhD
Head of Research, Institute of Primary and Community Care
University of Lucerne
Schwanenplatz 7
CH- 6004 Lucerne
Phone +41 41 410 88 85
Email: stefan.essig@iham-cc.ch

1.3 Statistician ("Biostatistician")

Dr. phil. nat. Tobias E. Erlanger
Clinical Trial Unit
Department of Clinical Research
University Hospital Basel
Spitalstrasse 12
CH- 4031 Basel
Phone +41 61 328 54 14
Email: tobias.erlanger@usb.ch
Statistical support

1.4 Laboratory

Not applicable.

1.5 Monitoring institution

CTU offer from 25.10.2019 and Contract (Please see Appendix)
Clinical Trial Unit (CTU)
Department of Clinical Research
University Hospital Basel
Spitalstrasse 12

CH- 4031 Basel

1.6 Data Safety Monitoring Committee

Not applicable.

1.7 Any other relevant Committee, Person, Organisation, Institution

Data Management

CTU offer from 25.10.2019 and Contract (Please see Appendix)

Clinical Trial Unit (CTU)

Department of Clinical Research

University Hospital Basel

Spitalstrasse 12

CH- 4031 Basel

Data Access Committee (DAC)

An independent Data Access Committee will be set-up and maintained by the CTU Basel. The study metadata will be prepared and published by the CTU on a digital repository compliant with the FAIR principles (19). The CTU will ensure the secure transfer and storage of the data at time of publication on secure servers maintained and backed-up by the IT-Department of the University Hospital Basel.

Hospital Pharmacy offer from 30.09.2019 (Please see Appendix)

Spital-Pharmazie

Qualitätssicherung

Universitätsspital Basel

Rosetti-Bau, Spitalstrasse 26

CH-4031 Basel

2. ETHICAL AND REGULATORY ASPECTS

The decision of the CEC (EKNZ, leading EC) and Swissmedic concerning the conduct of the study will be made in writing to the Sponsor-Investigator before commencement of this study. The clinical study can only begin once approval from all required authorities has been received. Any additional requirements imposed by the authorities shall be implemented.

2.1 Study registration

The OSPIC trial will be registered on the international trial register platform clinicaltrials.gov and on the Swiss National Clinical Trials Portal (SNCTP), www.kofam.ch. A request for registration on the SNCTP will be made at the time of the BASEC ethics submission. After the study is approved by the EKNZ, the protocol will be submitted for registration on clinicaltrials.gov.

2.2 Categorisation of study

According to Article 19 ClinO the present clinical trial corresponds to Category B as the medicinal product used (the drug Prednisone) is authorised in Switzerland, but will not be used in accordance with the prescribing information. We will test the efficacy of Prednisone treatment which is widely prescribed for post-infectious cough, even though there is limited evidence for its use for this condition. A placebo will be used as comparator to prevent various biases and to ensure a double-blinded assessment.

2.3 Competent Ethics Committee (CEC)

This study will be conducted in North-Western and Central Switzerland. For the North-western and Central Switzerland ethical approval will be sought and obtained from the EKNZ (CEC), which will act as the leading ethics committee for the OSPIC trial. This study will be coordinated by a single centre, uniham-bb, Liestal, in cooperation with the IHAM & CC centre in Luzern; the centres will manage collaboration with GPs in the North-western and Central parts of Switzerland.

All changes to the research protocol and/or planned research activity, and all unanticipated problems involving risks to humans, including the planned or premature study end, and the final report will be reported to the EKNZ. No changes will be made to the protocol without prior Sponsor and EKNZ approval, except when necessary to eliminate apparent immediate hazards to study participants. Premature study end or interruption of the study will be reported within 15 days. The regular end of the study will be reported to the EKNZ within 90 days and the final study report shall be submitted within one year after the study end. Amendments to the study protocol will be reported according to chapter 2.10.

2.4 Competent Authorities (CA)

The study will seek and obtain approval from the Swissmedic (Competent Authority) before starting the trial. Premature study end or interruption of the study will be reported within 15 days and the regular end of the study will be reported to the CA within 90 days. Amendments to the study protocol will be reported according to chapter 2.10.

2.5 Ethical Conduct of the Study

The study will be carried out in accordance to the protocol and with principles enunciated in the current version of the Declaration of Helsinki, the guidelines of Good Clinical Practice (GCP) issued by ICH, in case of medical device: the European Regulation on medical devices 2017/745 and the ISO Norm 14155 and ISO 14971, the Swiss Law and Swiss regulatory authority's requirements. The CEC and regulatory authorities will receive annual safety and interim reports and be informed about study stop/end in agreement with local requirements.

2.6 Declaration of interest

The investigators listed immediately below certify that they have no affiliations with or involvement in any organization or entity with any financial interest (such as honoraria; educational grants; participation in speakers' bureaus; membership, employment, consultancies, stock ownership, or other equity interest; and expert testimony or patent-licensing arrangements), or non-financial interest (such as personal or professional relationships, affiliations, knowledge or beliefs) in the medicinal product discussed in this study protocol.

- Prof. Dr. med. Andreas Zeller: No conflicts of interest.
- Prof. Dr. med. Jörg Leuppi: No conflicts of interest.
- Dr. med. Christoph Merlo: No conflicts of interest.
- Dr. med. Stefan Essig: No conflicts of interest.

2.7 Patient Information and Informed Consent

The OSPIC study will be advertised through a study poster and information leaflet displayed in collaborating GP practices. All potential participants in the study will be provided with a participant information sheet and a consent form describing the study and providing sufficient information for participant to make an informed decision about their participation in the study.

Participants who present for a GP consultation and meet the inclusion criteria for the OSPIC trial will be informed about the study by their GP. First of all, participating GPs will explain the nature of the study, its purpose, the procedures involved: taking 2 pills of 20 mg for 5 days at breakfast time, completing a survey and having calls with research staff at 7, 14, 28 days and 3 months after the GP visit. GPs will also inform potential participants about the expected duration of the study, the potential risks and benefits and any discomfort that may occur during participation in the study.

Potential participants will be given full written and verbal explanations and will be allowed ample time to ask questions. The GP carrying the consent process will answer all queries and then ask patients if they are interested in participating in the study. Patients will also be informed that participating in the study is voluntary and that they may withdraw at any time. Participants will be informed verbally and in writing that they can change their minds at any time and that withdrawal of consent will not affect their subsequent medical assistance and treatment. After receiving oral and written information about the study, patients will be given as much time as necessary to ask questions and read the written information carefully before deciding whether or not they wish to participate in the study.

Patients will be informed that their medical records may be examined by authorised individuals other than their treating physician. Contact information for the main investigator will be provided and participants will be encouraged to contact the research team by mail or phone to ask for further details or in case of difficulties (24 hours a day, 7 days a week, including public holidays).

Participants who decide to take part in the study will read and consider the statement before signing and dating the informed consent form. The consent form will be signed and dated by the person taking the consent, the GP, at the same time as the participant signs. The participant will receive a copy of the signed document from the GP. The consent form must also be signed and dated by the GP and it will be retained as part of the study records.

2.8 Participant privacy and confidentiality

The investigator affirms and upholds the principle of the participant's right to privacy and that they shall comply with applicable privacy laws. Especially, anonymity of the participants shall be guaranteed when presenting the data at scientific meetings or publishing them in scientific journals.

Individual subject medical information obtained as a result of this study is considered confidential and disclosure to third parties is prohibited. Subject confidentiality will be further ensured by utilising subject identification code numbers to correspond to data collected on paper and treatment data in the computer files.

For data verification purposes, authorised representatives of the Sponsor-Investigator (qualified personnel of the KSBL or the CTU), a competent authority (e.g. Swissmedic), or an ethics committee (EKNZ) may require direct access to parts of the medical records relevant to the study, including

participants' medical history.

2.9 Early termination of the study

The Sponsor-Investigator may terminate the study prematurely according to certain circumstances, for example:

- ethical concerns,
- insufficient participant recruitment,
- when the safety of the participants is doubtful or at risk, respectively,
- alterations in accepted clinical practice that make the continuation of a clinical trial unwise,
- early evidence of benefit or harm of the experimental intervention.

2.10 Protocol amendments

The coordinating centre of the study (Centre for Primary Health Care, uniham-bb, Liestal) in agreement with the collaborating centre Institute for Primary and Community Care in Lucerne is allowed to amend the protocol. Suggestions for protocol amendments can be made by collaborators and participating GPs on the study. Communications with GPs for the purposes of amending the protocol will take place in written form.

Substantial amendments will only be implemented after approval of the EKNZ (CEC) and Swissmedic (CA) respectively.

Under emergency circumstances, deviations from the protocol to protect the rights, safety and well-being of human subjects may proceed without prior approval of the sponsor and the EKNZ and Swissmedic (CEC/CA). Such deviations shall be documented and reported to the sponsor and the EKNZ and Swissmedic (CEC/CA) as soon as possible.

All non-substantial amendments are communicated to the Swissmedic (CA) as soon as possible if applicable and to the EKNZ (CEC) within the Annual Safety Report (ASR).

3. BACKGROUND AND RATIONALE

3.1 Background and Rationale

Cough as a symptom of respiratory infections is very common in primary care consultations and is one of the most common causes to seek medical advice in general practices (1). Cough after an URTI can be very bothersome and disabling in daily activities and has a significant impact on physical and psycho-social health, leading to impairment in QoL (3). Post-infectious cough - also known as subacute cough - is defined as lasting 3 to 8 weeks, following an URTI (2). It results from a protracted inflammation of the bronchial mucosa after a viral infection, an epithelial damage with irritant-receptors laid open, and/or a temporary bronchial hyper-responsiveness (2, 4). The diagnosis is based on the clinical history and physical examination excluding other causes such as COPD or asthma.

Recommendations regarding the management of post-infectious cough in general practice are scarce and inconsistent. We conducted a systematic review and meta-analysis of RCTs assessing the patient-relevant benefits and potential harms of available treatments for post-infectious cough to provide a wide overview of treatment options for primary care patients with post-infectious cough (6). Only six RCTs assessing diverse treatment regimens (i.e. inhaled fluticasone propionate, inhaled budesonide, salbutamol plus ipratropium-bromide, montelukast, nociception-opioid-1- receptor agonist, codeine, and gelatine) were identified. Most studies had an unclear or high risk of bias, and meta-analyses indicated no benefit of these regimens (6).

None of the individual RCTs found clear patient-relevant benefits for patients with post-infectious cough lasting 3 to 8 weeks. One study, however, indicated benefits of inhaled corticosteroids (20). Overall, there were two RCTs assessing inhaled corticosteroids for post-infectious cough (20, 21). Pornsuriyasak et al. (21) included a total of 30 patients and found no benefit of inhaled steroids on cough outcomes at all. The abovementioned trial by Ponsioen et al. (20) including 135 patients with cough for ≥ 2 weeks indicated an effect of inhaled steroids on cough in the overall study population, which was explained by beneficial effects in the sub-group of non-smokers. However, the study included also a relevant number of patients (n=44; 33%) without post-infectious cough lasting 3 to 8 weeks and reported no separate results for this group.

Clinical guidelines and recommendations on the use of inhaled corticosteroids are incoherent (2, 4, 5). A Cochrane review published in 2013 evaluated studies in which inhaled corticosteroids were tested in individuals with post-infectious or chronic cough. For post-infectious cough, this review identified only the same two studies as we did with our more recent searches. All other included studies assessed the effect of corticosteroids in patients with chronic cough. The authors concluded that no recommendation can be proposed due to the high heterogeneity and inconsistency of the studies and their results (22). A recently published RCT assessing adult patient without asthma with acute lower respiratory tract infection found no benefit of a 5-day treatment with 20mg of prednisone compared to placebo in terms of duration or severity of cough (17). Further, in a placebo-controlled RCT among patients with acute sore throat for at most seven days, a single oral dose of 10mg of dexamethasone did not increase the proportion of patients with resolution of symptoms at 24 hours. Overall, 55.9% of participants also reported a cough during the illness (23).

Many of the symptoms in post-infectious cough are thought to be mediated by inflammatory processes that are also present in exacerbations of asthma or COPD (7, 8). For these conditions, there is strong evidence showing that short-term oral corticosteroids provide patient-relevant benefits (9). Prednisone (tablets at a dose of 40 mg once daily for 5 to 7 days) is a well-established oral steroid for acute asthma or exacerbation of COPD.

Our systematic search identified no RCTs which assessed short-term use of oral corticosteroids for post-infectious cough (6), and the updated search in October 2018 also found none. We have also screened multiple study registers using the International Clinical Trials Registry Platform from the World Health Organization (last search 25 October 2018), identifying no trial with a similar aim as the proposed study. There is one registered trial aiming to assess the efficacy of inhaled budesonide in adult patients with chronic cough (Registration ID: NCT02715167). Other planned studies will assess the efficacy of corticosteroids in children with acute or chronic cough (Registration IDs: ACTRN12616001713482; ACTRN12615000132549; ChiCTR-TRC-13003182; ACTRN12611000589987) (24).

In summary, there is no established evidence-based treatment option for this very frequent condition in primary care and there is considerable uncertainty for or against using inhaled or oral corticosteroids. Only a well-conducted randomised placebo-controlled trial would determine the benefits and harms of

oral corticosteroids to treat patients with post-infectious cough in primary care. Hence, the main research question of this study is whether a 5-day treatment with orally administered prednisone can improve the cough related QoL as measured by the LCQ of adult primary care patients with post-infectious cough triggered by an URTI.

3.2 Investigational Product (treatment) and Indication

Prednisone is a synthetic corticosteroid drug in the dispensing class category B, dispensing requiring a medical prescription (25). Physiologically, corticosteroids are produced in the adrenal cortex and involved in various processes, such as immune response and regulation of inflammation, protein catabolism, carbohydrate metabolism and stress response. Prednisone has a potency which is four times greater compared to natural cortisol. Yet, its mineralocorticoid effect is by 40% smaller compared to natural cortisol. Prednisone is particularly effective as an immunosuppressant drug and is used to treat various inflammatory diseases.

3.3 Preclinical Evidence

Not Applicable. Prednisone is an approved-drug in Switzerland.

3.4 Clinical Evidence to Date

Prednisone (tablets at a dose of 40 mg once daily for 5 to 7 days) is a well-established oral steroid for acute asthma or exacerbation of COPD. Many of the symptoms in post-infectious cough are thought to be mediated by inflammatory processes that are also present in exacerbations of asthma or COPD (7, 8). For these conditions, there is strong evidence showing that short-term oral corticosteroids provide patient-relevant benefits (9).

Oral corticosteroids are used for decades in primary care. For additional information please see section 3.1 ("Background and Rationale").

3.5 Dose Rationale: Rationale for the intended purpose in study

For this study we select a dose of 40mg (2 tablets of 20 mg) of prednisone once daily over 5 days because of pharmacokinetic evidence suggesting that a minimum dose of 20 mg prednisone daily is required for non-asthmatic patients to achieve an adequate anti-inflammatory effect (26) and because the first trial of its kind should use an adequate and sufficient dose to detect any potential effects, as concluded in a systematic review by El-Gohary et al. (5): "adequately powered research is warranted, in primary care, to further clarify the benefits and harms of corticosteroids for adults with acute and post-infectious cough. Because it is not clear from this review where the dose-response threshold lies, it would appear sensible to test the effects of high-dose (e.g. 40 mg prednisone daily) oral steroids first. If no effect is seen, it would seem unlikely that even high dose inhaled steroids would be effective". We select a treatment duration of 5 days since post-infectious cough is thought to be mediated by inflammatory processes comparable to those in exacerbations of asthma or COPD. For these conditions there is strong evidence that short-term oral corticosteroids for 5 days provide patient-relevant benefit without relevant harm (9).

3.6 Explanation for choice of comparator (or placebo)

Placebo will be used as comparator to prevent various biases and to ensure a double-blinded assessment. From an ethical point of view a placebo-controlled trial seems justified since there is no established therapy for post-infectious cough.

3.7 Risks / Benefits

So far, recommendations regarding the management of post-infectious cough in general practice are scarce and inconsistent. Results from this trial will determine the clinical effectiveness of oral corticosteroids for the treatment of post-infectious cough and may establish the first treatment option with clear patient-relevant benefits at low costs in this common condition. Participation in this study can help other patients with post-infectious cough to receive effective treatment.

Due to the short intervention of 5 days of prednisone, no treatment modifications are planned unless in the (unlikely) occasion of side effects. Even though the likelihood is very low Adverse Events (AE), such as

allergic reactions to the study drug, psychotic, or pre-psychotic episode, or Serious Adverse Events (SAE), sepsis, venous thromboembolism, fracture, can occur (27). In terms of side effects, a recently published retrospective cohort showed even a short time use of corticosteroids increases the incidence of severe adverse events such as sepsis or venous thromboembolism (28). Therefore, safety issues even with short time corticosteroids regimens are crucial. We feel that using the follow-up proposed for our trial will these concerns. A certain reassurance in terms of safety comes from the randomized-controlled trial conducted by Hay (17). In this study, assessing the effects of oral corticosteroids for acute lower respiratory tract infection in adults without asthma (prednisone 40mg once daily for 5 days, i.e. same dose as in our trial), no serious adverse events were observed (17). In any of the cases of adverse events occurring, as well as when other urgent reasons arise (pregnancy, new cancer diagnosis, infection other than URTI) (27), prednisone treatment will be stopped immediately. Other side-effects have been documented during short-term prednisone treatment: increase of appetite and activity, increased risk of infection, headache, glucose metabolism disorder (28). Study participants will be followed-up at several time points from enrolment in the study and experienced and trained study staff will call patients on day 7,14, 28 and at 3 months and will ask participants to answer questions about outcomes and also the occurrence of AE or SAE. At the time of enrolment, participants will also be advised by their GP to contact the family practice or the study team in the event of any side-effects. We think that using the follow-up proposed for this study will mitigate the safety issues potentially posed even with short time corticosteroids.

Patients will not receive any financial compensation for study participation as the burden for them is minimal and they do not have any additional expenses for travelling, medical devices or other items.

3.8 Justification of choice of study population

Patients with post-infectious cough and who are older than 18, and present to GP practices will be informed about the study and invited to participate. Patients who decide to participate will be screened for eligibility. All participating subjects will sign an informed consent.

Patients who are unable to follow the study instructions or not able to give informed consent cannot participate. Female patients who are pregnant, breastfeeding or who are planning to get pregnant will be excluded from the study. Participants must also be able and willing to participate in the follow-up research interviews conducted by telephone.

4. STUDY OBJECTIVES

4.1 Overall Objective

The purpose of this study is to assess whether a 5-day treatment with orally administered prednisone provides patient-relevant benefits by improving the cough-related QoL of patients with post-infectious cough triggered by an URTI and seeking care in adult primary care practices. The study aims to describe an efficacy and safety profile for a 5-day prednisone treatment compared to a 5-day course of placebo.

4.2 Primary Objective

To investigate whether a 5-day treatment with orally administered prednisone will be superior to placebo and improve the cough-related QoL after 14 days in patients with post-infectious cough after an URTI.

4.3 Secondary Objectives

To evaluate the impact of oral prednisone on:

- Cough-related QoL at other time-points (7, 28 days and 3 months after randomization)
- Three cough-related QoL (LCQ) sub-domains, i.e. physical, psychological, and social
- Overall cessation of cough, i.e. proportion of patients without cough symptoms 7, 14, and 28 days after randomization

4.4 Safety Objectives

To evaluate the impact of oral prednisone on:

- Safety outcomes, i.e. re-consultations at GP and/or hospitalisations
- Total AE within 3 months after randomization
- SAE within 3 months after randomization.

To assess any potential harms, we will evaluate any AE and specifically SAE according to the WHO-UMC causality categories “certain”, “probable”, “possible”, and “unlikely” (16).

5. STUDY OUTCOMES

5.1 Primary Outcome

The primary outcome is *cough-related QoL assessed by the LCQ score 14 days after randomisation.*

To assess the impact of the treatment and control medication on patients' QoL, the LCQ will be used. The LCQ takes 5 to 10 minutes to complete (10). The LCQ has already been used in a similar randomised-controlled trial assessing the effectiveness of montelukast in the treatment of post-infectious cough (15). The LCQ is a validated QoL measurement tool for non-specific cough, developed for self-administration and assesses the impact of cough on various aspects of life, including emotions, sleeping behaviour, work and relationships. It contains 19 items which are divided over 3 domains: physical (8 items), psychological (7 items) and social (4 items); with a 7-point Likert scale (11). The LCQ is short, easy to administer, suitable for capturing longitudinal developments in cough and cough-related wellbeing, and can be useful in clinical trials assessing new medications for cough (11). We will use the validated German version of the original LCQ (13), which is kindly provided by Prof. Dr. J. Müller-Quernheim, Head of Pneumonology, Department of Medicine, University of Freiburg im Breisgau, Kilianstrasse 5, 79106 Freiburg im Breisgau, Germany.

5.2 Secondary Outcomes

- Cough-related QoL assessed by the LCQ score 7, 28 days and 3 months after randomisation.
- Cough-related QoL sub-domains physical, psychological, and social 7, 14, 28 days and 3 months after randomisation.
- Overall cessation of cough 7, 14, 28 days and 3 months after randomisation.
- Incidence rate of re-consultations at GP and/or hospitalisations within 3 months following randomisation.

5.3 Other Outcomes of Interest

None.

5.4 Safety Outcomes

- Incidence rate of re-consultations at GP and/or hospitalisations within 3 months following randomisation
- Total AE within 3 months after randomization
- SAE within 3 months after randomization
- Changes in glucose levels for patients with pre-study controlled diabetes that are deemed by GP to exceed the hypothesized benefit on cough.

6. STUDY DESIGN

6.1 General study design and justification of design

OSPIC is a 1:1 randomised, parallel-group, placebo-controlled, superiority trial with blinded patients, physicians and outcome assessors in a primary health care setting.
Recruitment will take place in GP practices in the Northwestern, Central Switzerland and the Canton of St. Gallen. These GPs will recruit 5 to 10 patients over an 18-months period to reach the target sample size (N=204).

To ensure that the study is conducted in accordance with GCP-guidelines, GPs will receive study-specific training prior to study start. In addition, GPs will be provided with a tailored GCP training specific for the responsibilities they assume for this study, including training on providing patient information and consenting and on detection, documentation and reporting of adverse events. The training consists of a video and written transcript of the key points presented in the video. The video training material was developed by the *Institut für Hausarztmedizin Bern* together with CTU Bern based on CTUs GCP course material. This GCP training was accepted by the *Kantonale Ethikkommission des Kantons Bern* for two recently submitted clinical trials: Optimizing Pharmacotherapy In the Multimorbid Elderly in Primary CAre: the OPTICA Trial and the Efficacy of Metamizole versus Ibuprofen and a Short Educational Intervention versus Standard Care for patient with Acute and Subacute Low Back Pain: A Randomized, Factorial Trial. EMISI Studie (PI Prof. M. Wertli, Bern) The video can be accessed at https://video.aum.iml.unibe.ch/BIHAM-Videos/, under the name file *Instruktionsvideo für Hausärzte-innen_v111018.mp4*. A transcript of the instruction video is attached to the section 17. Appendices. GPs will have to confirm that they watched the video and read the training material before they start recruiting patients. A platform like ILIAS (or similar) will be used to deliver the training and submit the confirmation.

Patients with post-infectious cough presenting in practices will be given full written and verbal explanations of the trial purpose, potential benefits and risks, and then will be invited to participate. Patients who agree to join the study will be screened against the full eligibility criteria. Participants will have sufficient time to ask questions and it will be stated clearly that non-participation does not influence the standard of care in any way. Formal written consent will be taken from those agreeing to participate.
Once patients are informed about the OSPIC trial (by their GP) and written informed consent is obtained (day 0), they will be randomly allocated to prednisone or placebo treatment. We select a dose of 40mg (2 tablets of 20 mg) of prednisone once daily over 5 days because of pharmacokinetic evidence suggesting that a minimum dose of 20 mg prednisone daily is required for non-asthmatic patients to achieve an adequate anti-inflammatory effect (26) and because the first trial of its kind should use an adequate and sufficient dose to detect any potential effects, as concluded in a systematic review by El-Gohary et al. (5): "adequately powered research is warranted, in primary care, to further clarify the benefits and harms of corticosteroids for adults with acute and post-infectious cough. Because it is not clear from this review where the dose-response threshold lies, it would appear sensible to test the effects of high-dose (e.g. 40 mg prednisone daily) oral steroids first. If no effect is seen, it would seem unlikely that even high dose inhaled steroids would be effective". We select a treatment duration of 5 days since post-infectious cough is thought to be mediated by inflammatory processes comparable to those in exacerbations of asthma or COPD. For these conditions there is strong evidence that short-term oral corticosteroids for 5 days provide patient-relevant benefit without relevant harm (9).
After informed consent is obtained, GPs will provide participants with the pre-randomized study medication. Oral and written instructions on how the medication should be taken will be provided to the study participants. In the event of a missed dose, patients are instructed to continue medication intake on the next day. Participants who complete the study are required to return empty medication glass jars to their GPs as soon as this convenient for them (e.g. on the occasion of the next consultation). Participants will be asked by the research staff/ study nurse about their medication intake at the first phone call on day 7.
The expected duration of participant's participation is around 3 months. The participation includes day 0 (enrolment, information on the study, informed consent, allocation to study group, completion of the LCQ with their GP, administration of study drugs), the treatment period of 5 days, and the follow-up phone call by the research staff/study nurse on days 7, 14 , 28 and at 3 months after randomization. The telephone calls for the study follow-up are expected to last around 15 minutes each. In case phone calls are not answered, follow-up phone calls will be performed several times and participants will be sent reminder emails. If the participant is not reached for the follow-up call at day 7, then a call will be made in the next 2

days (days 7 +2). When participants are not reached for the follow-up calls at days 14 and 28, they will be called again on the next two days (day 14 +2; days 28 +2). If participants are not reached by telephone at 3 months, they will be called by the research staff during the next 7 days (3 months +7 days).

To include 204 participants the recruitment period will last 18 months and cover two winter seasons when the incidence of URTIs is very high and post-infectious cough is very common. The length of the recruitment period was especially defined to include more than one winter season, to ensure successful recruitment and even though coughing is prevalent throughout the year and patients can be affected in summer as well.

All GPs, clinical investigators and research staff/study nurses involved in the study as well as all patients will remain blinded with respect to the randomisation throughout the trial. The study medication is labelled, packed and dispensed by the University Hospital Basel Pharmacy according to the randomisation procedure. If safety concerns require to know the patient's allocated intervention, the GP can request an unblinding provided by the CTU using the secuTrial®. Each unblinding is documented in the EDC's integrated audit trail system.

At baseline visit, patients will be randomly assigned in a 1:1 ratio either to the active treatment or the control group. Patients are considered being smokers, when answering to the baseline questions that they had smoked "more than 100 cigarettes in their life", that they smoke "daily" or "sometimes" (29).

6.2 Methods of minimising bias

6.2.1 Randomisation

At baseline visit, patients will be randomly assigned in a 1:1 ratio either to the active treatment or the control group. Pre-randomised, identically looking, numbered and marked medication glass jars with 5 daily doses of 40 mg (2 tablets of 20 mg) of prednisone (intervention group) or placebo (control group) are provided by GPs to participants. The randomisation procedure will be implemented by the CTU of the University Hospital Basel. The CTU Basel will generate a randomisation list with a 1:1 treatment allocation. This list will be the basis for labelling and packing of the study medication in cooperation with the University Hospital Basel Pharmacy. The study medication will be directed to the GPs who will hand participants the pre-randomized medication glass jars in the order of reception of the medication glass jars. Following this procedure, participants will therefore be randomly allocated to wither prednisone or placebo medication. With the randomisation list being only accessible by the CTU Basel and the University Hospital Basel Pharmacy, the treatment allocation is concealed from patients, physicians and other involved personal.

6.2.2 Blinding procedures

All GPs, clinical investigators and research staff/study nurses involved in the study as well as all patients will remain blinded with respect to the randomisation throughout the trial. The study medication is labelled, and packed and dispensed by the University Hospital Basel Pharmacy according to the randomisation list provided by the CTU Basel. Participants in the study will receive a medication guide stating that they are taking either placebo or prednisone tablets (40 mg) daily for a period of 5 days as part of the OSPIC study. In case participants require hospitalizations or they consult a different doctor (not their GP), they are encouraged to take the medication guide with them. GPs will not have access to the randomization list and in case of urgency they have to request the unblinding by making a request with the OSPIC study team.

6.2.3 Other methods of minimising bias

None.

6.3 Unblinding Procedures (Code break)

If safety concerns require to know the patient's allocated intervention, the GP can request an unblinding by making a request with the OSPIC study team who will inform the CTU as soon as possible (during

working hours). After receiving the unblinding request the CTU can break the code by using the secuTrial®. Each unblinding is documented in the EDC's integrated audit trail system.

7. STUDY POPULATION

The aim is to enrol a total of 204 patients with post-infectious cough within a period of 18 months. Patients will be recruited by participating GPs running a general practice in North-western and Central Switzerland. The geographical area is large enough to recruit the required number of patients in the indicated timeframe as nearly 40% of adults can be affected by post-infectious cough after an acute respiratory infection (15, 28). Patient recruitment in primary practices in the German speaking part of Switzerland will continue until the sample size is reached. It is envisaged that the recruitment period will last about 18 months: First-patient-in in February 2020 and last-patient-out in July 2021. We expect that most participants will be enrolled during the two winter seasons when the incidence of URTIs is very high and post-infectious cough is very common. The length of the recruitment period was specifically defined to include more than one winter season, to ensure successful recruitment and even though coughing is prevalent throughout the year and patients can be affected in summer as well. In case of unforeseen difficulties with patient recruitment and lack of a reasonable number of participants after 9 months (e.g. less than 1/3 of target study population enrolled), the recruitment areas can be enlarged since the investigators have established cooperation with institutes of "Hausarztmedizin" in the Eastern part of Switzerland and in the French speaking part of Switzerland. Additionally, the number of randomised patients per GP (limited to 10) can be increased. If the enrolment goals are not met, the study will be submitted to other regional ethics committees in order to expand this study geographically.

7.1 Eligibility criteria

Participants fulfilling all of the following <u>inclusion</u> criteria are eligible for the study:

- Age ≥ 18 years
- Seeing a GP for a dry or productive post-infectious cough (3 to 8 weeks) after an URTI
- Able and willing to give informed consent by themselves

The presence of any one of the following <u>exclusion</u> criteria will lead to exclusion of the participant:

- Patients with hypersensitivity to prednisone or to one of the adjuvants in the drug's composition
- Patients with known or suspected diagnoses associated with cough, such as: pneumonia, allergic rhinitis, sinusitis, bronchial asthma, chronic pulmonary disease (COPD), gastroesophageal reflux disease
- Patients with other chronic disease such as bronchiectasis, cystic fibrosis, cancer, tuberculosis, heart failure
- Use of inhaled or oral corticosteroids within the last four weeks
- Immunodeficiency/immunocompromised state (e.g. cancer chemotherapy, HIV infection)
- Pregnancy/breastfeeding, as evaluated through screening
- Regular treatment known to be associated with cough (e.g. angiotensin-converting enzyme inhibitors)
- Patients with a documented diagnosis of glaucoma or osteoporosis in the GP's patient health record
- Experienced fractures due to osteoporosis
- Patients with uncontrolled diabetes (as deemed by GPs who appraise whether the potential side effects of short-time corticosteroids on glucose levels exceed the hypothesised benefit on cough)

It is at the discretion of the treating GP to physically examine the patient. GPs will screen all female participants of reproductive age (postmenarche to menopause) using a battery of questions and will exclude all females who are pregnant, plan or are actively trying to get pregnant or women who are potentially pregnant. Women potentially pregnant are defined as those women who respond that they are not using safe contraception or that they think they are pregnant or that they may be pregnant. Safe contraception is considered to be the use of the following contraception methods: oral contraceptive drugs, birth control implant such as Implanon NXT®, intrauterine device or coil such as Mirena®, every 3-months contraceptive injection e.g. Depo-Provera 150® or always using condoms during intercourse.

Women with anamnestic risk of pregnancy shall not be included in this study (unprotected sexual intercourse in the last two weeks). Pregnancy testing (laboratory or at-home tests) is not required for this study. Please see section 10.1.2 for additional information on handling risk of pregnancy for this study.
The GP may perform different tests if necessary (i.e. laboratory test, pulmonary function test). Also, GPs will appraise whether the potential side effects of short-time corticosteroids on glucose levels exceed the hypothesized benefit on cough, particularly in diabetic patients.

7.2 Recruitment and screening

Family practice will be identified through existing research networks by the Centre for Primary Health Care, University of Basel, and the Institute of Primary and Community Care, University of Lucerne, through personal contact, and by mail. In total 472 GPs of this network in North-western and Central Switzerland have already been approached by an online survey in November 2016. Of these, 183 GPs replied (39%). A case vignette (32-year old, healthy non-smoker without asthma, who has had dry cough for 4 weeks) was presented to them and they were asked about their management of post-infectious cough, namely if they would prescribe topical or oral steroids and whether they would take part in a trial assessing the treatment of post-infectious cough. Results were presented at the SSGIM (Swiss Society of General Internal Medicine) congress in Lausanne in May 2017. Overall, 52% (n=94) of GPs indicated to agree to participate in an intervention study assessing the efficacy of corticosteroids in patients with post-infectious cough (30).

Patient recruitment will take place in primary care practices in the German speaking part of Switzerland and will continue until the sample size is reached. Study leaflets and posters will be distributed to collaborating GPs to advertise the study in their family practices. To ensure uniform questioning during the trial (LCQ) and to minimise differences, GPs and research staff/study nurses will receive a trial specific training by CTU and study team members. In case of any queries, Prof. Dr. med. Andreas Zeller or the study nurse can be contacted at any time via phone or email. It is envisaged that the recruitment period will last about 18 months: First-patient-in in February 2020 and last-patient-out in July 2021. We expect that most participants will be enrolled during the two winter seasons. However, clinical experience indicates numerous cases of post-infectious cough also during warmer periods of the year (31).

Patients with post-infectious cough presenting in practices will be given full written and verbal explanations of the trial purpose, potential benefits and risks. They will have sufficient time to ask questions and it will be stated clearly that non-participation does not influence the standard of care in any way. Patients who agree to participate will be screened for eligibility. Formal written consent will be taken from those agreeing to participate. The paper CRF will be completed by the GP and the medication glass jars containing prednisone or placebo will be handed to the participant. The number of randomised patients per GP is limited to 10. In case of recruitment problems (scheduled numbers of study participants not reached at predefined milestones) this limit can be increased.

Patients will not receive any financial compensation for study participation as the burden for them is minimal and they do not have any additional expenses for travelling, medical devices or other items. GPs will get a refund of 100 CHF per recruited patient to cover their fixed costs. The 100 CHF value for compensation is based on a review of financial data from basic providers in the canton Zürich published in "Schweizerische Ärztezeitung" in 2004 (32).

7.3 Assignment to study groups

At baseline visit, patients will be randomly assigned in a 1:1 ratio either to the active treatment or the control group. The randomisation procedure will be implemented by the CTU of the University Hospital Basel and a randomization list will be generated (see also section 6.2.1). This list will be provided to the University Hospital Basel Pharmacy for the purpose of labelling and packaging the medication. The medication packages will be delivered to GPs who will distribute the medication in the order of reception and therefore randomly allocate recruited participants to either the treatment or the control medication.

7.4 Criteria for withdrawal / discontinuation of participants

Due to the short intervention of 5 days, no treatment modifications are planned unless in the (unlikely) occasion of AE (e.g. allergic reactions to the study drug, psychotic, or pre-psychotic episode) or occurrence

of SAE (e.g. sepsis, venous thromboembolism, and fracture) (27) or other urgent reasons (pregnancy, cancer diagnosis, infection other than URTI). In any of those cases the treatment will be stopped immediately. Treatment will also be discontinued when study participants withdraw their informed consent. Study participants are given the possibility to withdraw from the OSPIC trial without justification at every stage and at any time.

In the follow-up phone calls participants will be asked about concurrent medication, including if they started a treatment with antibiotics. In the case that participants are prescribed and start the antibiotics treatment during the study's 5-days medication, then those participants will be discontinued from the study.

8. STUDY INTERVENTION

8.1 Identity of Investigational Products (treatment)

Participants in the study will be given by their GP identically looking, numbered and marked medication glass jars with 5 daily doses of 40 mg (2 tablets of 20 mg per dose) of prednisone (intervention group) or placebo (control group). Oral and written instructions on how the medication should be taken will be provided to the study participants. The dosing schedule is very convenient for the participant as the medication needs to be taken only once a day during breakfast and for a clearly defined and limited timeframe of 5 days.

PREDNISON Galepharm Tabl. 20 mg will be delivered by Galepharm AG, 8700 Küsnacht (ZH). For further information and details see product information of "PREDNISON Galepharm Tabl. 20 mg" (https://compendium.ch/prod/prednison-galepharm-tabl-20-mg/de). PREDNISON Galepharm as well as placebo tablets are manufactured according to Good Manufacturing Practice (GMP)-guidelines.

The University Hospital Pharmacy at the University Hospital Basel will procure the Prednison 20 mg Galepharm and the visually identical placebo tablets and the packaging material: medication glass jars and medication labels. The Pharmacy will be responsible for the manufacturing and the documentation of the investigational product and placebo used in this study.

8.1.1 Experimental Intervention (treatment)

Patients in the intervention group will receive 2 white tablets of PREDNISON Galepharm Tabl 20 mg (oral corticosteroid) to be administrated orally once daily for 5 days. One tablet contains the active substance Prednison (20 mg).

For further information and formulation details see product information for PREDNISON Galepharm Tabl. 20 mg, Swissmedic authorization 50821, Galepharm AG Zürich (https://compendium.ch/prod/prednison-galepharm-tabl-20-mg/de).

8.1.2 Control Intervention (standard/routine/comparator treatment)

Patients in the control group will receive identically looking placebo (10 pills) manufactured by Apotheke Hotz, Zürichstrasse 176, CH- 8700 Küsnacht. Each white tablet has a break mark, an average diameter of 9.00 mm, and an average height of 2.7 mm, identical to the verum tablets. Placebo tablets will be administered orally (40 mg of placebo once daily; 2 tablets of 20 mg) for 5 days. The content of the placebo tablets is as follows: Lactose monohydrate 140 mg, microcristalline cellulose 68 mg, Croscarmellose sodium 5 mg, Magnesium stearate 2 mg.

8.1.3 Packaging, Labelling and Supply (re-supply)

The University Hospital Basel Pharmacy will package and label the study medication with alphanumeric codes and dispense it according to the randomisation procedure i.e. 10 identically looking tablets of prednisone (intervention group) and placebo (control group). Tablets will be packaged in numbered and labelled medication glass jars with 5 daily doses of 40 mg (2 tablets of 20 mg) Prednison or placebo. The randomization list is provided by the Clinical Trial Unit Basel. Labeled glass jars containing 10 tablets each will be prepared as packages of 10 glass jars at the Hospital Pharmacy pick up by the study staff and delivered to every collaborating GP by study staff.

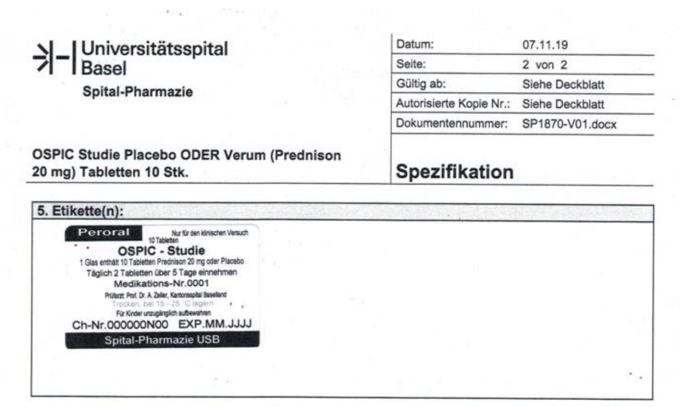

Figure: Labelling sticker for the medication glass jars

8.1.4 Storage Conditions

The medication must be stored in a dry place and at ambient temperature (15 – 25 °C). All medication (placebo and prednisone) is supplied, stored and eventually destroyed according to standard procedures at the University Hospital Basel Pharmacy (Please see Appendix Hospital Pharmacy Offer, 30Sep2019). The packed medication will be stored at the Pharmacy of University Hospital Basel at room-temperature up to the point of pick-up for distribution to GPs. The Hospital Pharmacy will continuously monitor room temperature and has a separate room with restricted access for all investigational medicinal products. The GPs and participant should store the glass jar in a dry place at room temperature.

8.2 Administration of experimental and control interventions

8.2.1 Experimental Intervention

Two tablets of PREDNISON Galepharm Tabl. 20 mg will be taken orally by the patient once daily during breakfast. The intervention group will receive prednisone for five days. Follow-up time will be 3 months from the start of treatment. In the event of a missed dose, patients are instructed to continue medication intake on the next day. Please see Appendix OSPIC Medication Guide_V1.0.

8.2.2 Control Intervention

Two tablets of placebo will be taken orally by the patient once daily during breakfast. The control group will receive placebo for five days. Follow-up time will be 3 months from the start of treatment. In the event of a missed dose, patients are instructed to continue medication intake on the next day. Please see Appendix OSPIC Medication Guide_V1.0.

8.3 Dose modifications

Due to the short intervention of 5 days, treatment modifications are not anticipated. However, treatment modifications are planned in the (unlikely) occasion of AE (e.g. allergic reactions to the study drug, psychotic, or pre-psychotic episode) or occurrence of SAE (e.g. sepsis, venous thromboembolism, fracture) (27) or other urgent reasons (pregnancy, cancer diagnosis, infection other than URTI). In any of those cases the treatment will be stopped immediately.

Treatment will also be discontinued when study participants withdraw their informed consent.

Treating doctors can change to open-label treatment and adjust treatment if they deem necessary for the benefit of the patients, such as in cases when the patients' clinical condition is worsening or the patient presents to the GP for consultation before the 5-day treatment is over. Throughout the trial, participants' treatment can be re-evaluated by their GPs based on clinical needs and GPs independently decide about additional treatment options. Please also see 7.4 Criteria for withdrawal/ discontinuation of participants.

8.4 Compliance with study intervention

Oral and written instructions on how the medication should be taken will be provided to the study participants. Participants are required to return empty medication glass jars to their GPs as soon as this convenient for them (e.g. on the occasion of the next consultation). Participants will be asked by the research staff/ study nurse about their medication intake at the first phone call on day 7. In the event of a missed dose, patients are instructed to continue medication intake on the next day.

GPs will inform the patient in depth on the importance to adhere to the 5-day medication intake to ensure effectiveness of the treatment. The GPs will also remind patients on the importance for the trial conduct to be available for the follow-up phone calls. Furthermore, the dosing schedule is very convenient for the participant as the medication needs to be taken only once a day during breakfast and for a clearly defined and limited timeframe (5 days). Experienced and trained research staff/study nurses will call patients by phone on day 7, 14 28 and at 3 months to complete the LCQ and to answer questions about other outcomes, in particular about medication intake (only on day 7), possible re-consultations with their GP, possible hospitalisation and the occurrence of AE or even SAE.

8.5 Data Collection and Follow-up for withdrawn participants

Participants in the study will be asked at each follow-up call at day 7, 14, 28 and after 3 months from enrolment whether they are still happy to participate in the study. Participants who wish to withdraw have the possibility to either (1) inform their GP who then records this in the participant file; (2) inform the study team directly by phone or mail; (3) inform the research staff at one of the follow-up phone calls. Participants who have withdrawn from the study will not be followed-up if they do not wish to be contacted. Data collected until the time of withdrawal will be used for the intention to treat analysis.

8.6 Trial specific preventive measures

Any co-treatment or co-medication (i.e. antitussives, inhalation, herbal teas, and homoeopathic pharmaceuticals) is permitted except for the use of corticosteroids. The use of any other medical intervention by study participants will be recorded in electronic Case Report Forms (eCRF) to analyse potential influence on outcomes.

GPs will screen all female participants of reproductive age (postmenarche to menopause) using a battery of questions and will exclude all females who are pregnant, plan or are actively trying to get pregnant or women who are potentially pregnant. GPs will advise participants that they need to use contraceptives for the duration of the study and that they should inform the GP or the study team in case they suspect they have become pregnant. Women with anamnestic risk of a pregnancy shall be excluded from this study. If a participant gets pregnant during follow-up, course and outcome of the pregnancy should be followed up carefully by GPs, documented and reported. Please see section 10.1.2 for additional information on handling risk of pregnancy for this study.

8.7 Concomitant Interventions (treatments)

See section 8.6 ("Trial specific preventive measures").

8.8 Study Drug Accountability

The University Hospital Basel Pharmacy is responsible for all manufacturing and logistic documentation of the study medication up to the point of distribution to the GPs. Study medication packages will be stored in appropriate conditions at room temperature in a separate room dedicated to investigational medicinal products before being handed over to the study team which is responsible for delivering it to collaborating GPs All participating GPs will receive a package with the required and pre-randomized medication necessary to recruit up to 10 participants. GPs will be in charge for the adequate storage of the study medication package and dispensing it according to protocol.

The Hospital Pharmacy documents in its accountability log the amount, the date and medication numbers of every manufactured medication batch and the picking up by study staff.

The GP documents the acknowledgement of receipt, the inventory at the site, the dispensing of the medication to the patient, and the return or alternative disposition of unused medication. The documentation should include dates, quantities, batch number, expiry date, and medication number, and this documentation should be available to the study monitor for review upon request.

8.9 Return or Destruction of Study Drug

Return or destruction of left-over study medication are handled according to standard procedures and are the responsibility of the local study team.

Participants who complete the study are required to return empty medication glass jars to their GPs as soon as this convenient for them (e.g. on the occasion of the next consultation). Participants will be asked by the research staff/ study nurse about their medication intake at the first phone call on day 7.

In the case that the study medication intake must be stopped prematurely due to, for example, diagnosis of another serious illness or pregnancy, those participants will be asked to return the remaining study medication to their GP.

GPs who choose to stop collaboration in the study before reaching the recruitment target will have to return all study medication and documentation to the local study team in Liestal. In case that at the study end there is spare medication that was not used, those drugs will be returned to the local study team in Liestal who will be responsible to dispose of it.

Unused or expired medication may be returned by the local study team to the Hospital Pharmacy for destruction. In this case the destruction of the medication will be performed by an authorised company via high temperature incineration.

9. STUDY ASSESSMENTS

9.1 Study flow chart(s) / table of study procedures and assessments

Study Schedule

	**STUDY PERIOD**						
	Enrolment	Randomisation	Treatment	Follow-up ($T_{days/months}$)			
TIME POINT	$T_{baseline}$	T_0	$T_{days\ 1\text{-}5}$	T_{day7}	T_{day14}	T_{day28}	$T_{3months}$
**ENROLMENT**							
Eligibility	X						
Informed consent	X						
Allocation		X					
**INTERVENTION**			X				
**ASSESSMENTS**							
Baseline characteristics		X					
LCQ		X		X	X	X	X
Adherence to treatment				X			
Cessation of cough				X	X	X	X
Re-Consultations*				X	X	X	X
Hospitalisations				X	X	X	X
Concomitant treatments		X		X	X	X	X
Adverse events				X	X	X	X
Serious adverse events				X	X	X	X

* Clinical follow-up visits with the GP are at the discretion of the treating GP and/or based on patient's needs

Study Assessments Flowchart

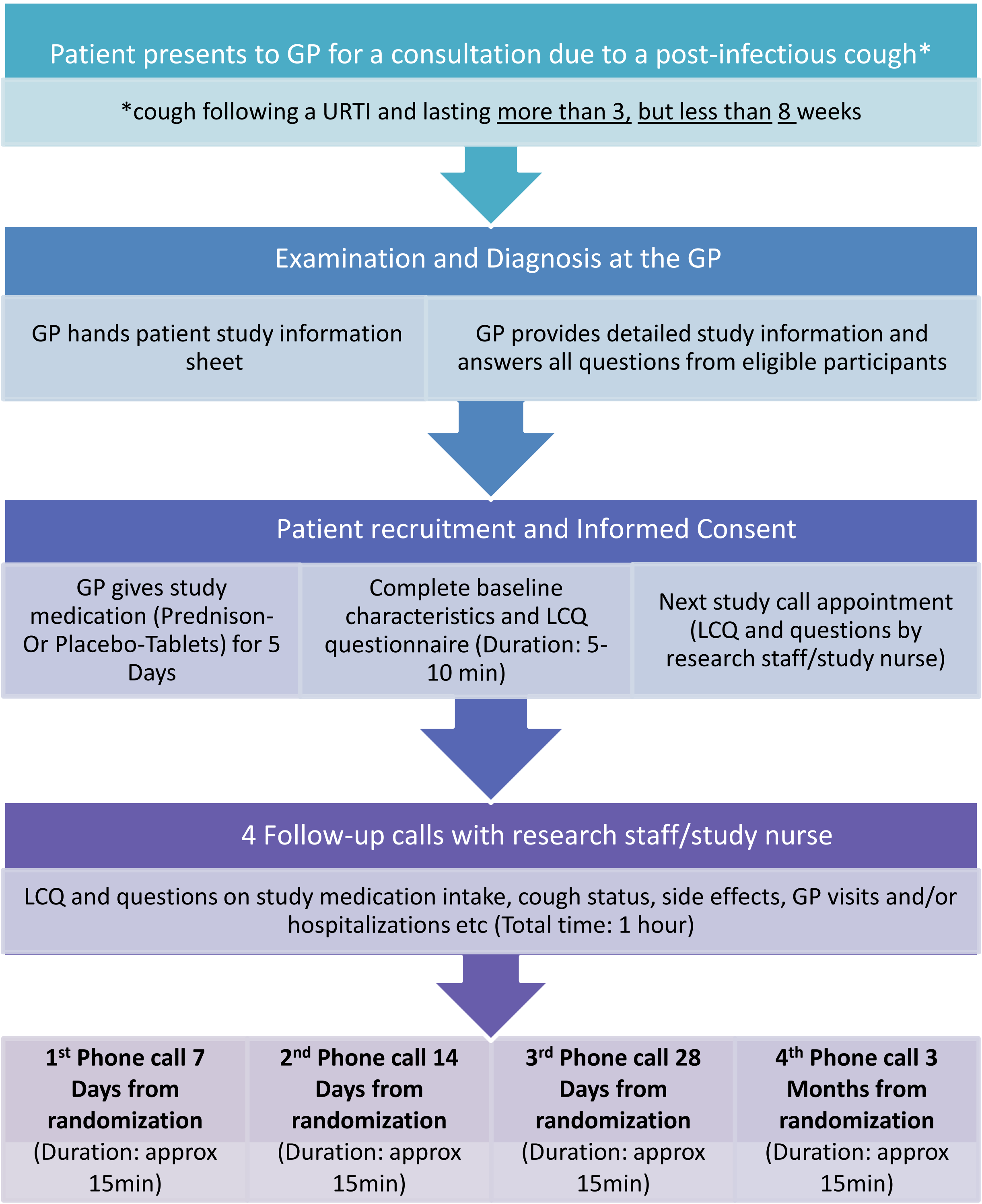

9.2 Assessments of outcomes

On days 7, 14, and 28 and at 3 months patients will be called by a trained and experienced research staff/study nurse, and asked to complete the LCQ on the phone. Appointments for the next phone calls will be set during the previous phone call. At the follow-up phone calls the research staff/ study nurses will also inquire about the presence of cough (binary variable yes/no), adherence (day 7), number of re-consultations with the GP, hospitalisations for potential illness deterioration and the occurrence of AE and SAE events. Incidence rates of AE and SAE will be assessed in WHO-UMC (16) causality categories “certain”, “probable”, “possible”, and “unlikely” during 3 months following randomisation.

In case the phone is not answered, follow-up phone calls will be performed several times over the next days: at day 7 (+ 2 days), at day 14 (+2 days), and 28 (+2 days) and at 3 months (+7 days). Participants will also be sent reminder email provided the email was given by the participant. Data at follow-up will be recorded with eCRF. For each enrolled study participant, an eCRF is maintained. eCRFs must be kept current to reflect subject status at each phase during the course of study. Participants must not be identified in the eCRF by name or initials and birth date. Appropriate coded identification (patient ID) is used. GPs and the research staff/study nurses will be authorized to do paper and eCRF entries. Source data will be available at all GPs to document the existence of the patients. To ensure uniform questioning during the trial (LCQ) and to minimize differences GPs and research staff/study nurses will receive a trial specific training and standard operating procedure leaflet prior to study start.

9.2.1 Assessment of primary outcome

At baseline GPs will hand participants the LCQ and will be available to answer question. Participants will complete the LCQ on paper. For the follow-up the LCQ will be administered by trained research staff and recorded electronically.

The LCQ total score (addition of domain scores) ranges from 3-21; a higher score corresponds to a better health status (30). Total and individual domain LCQ scores will be calculated. Please also see section 5.1.

9.2.2 Assessment of secondary outcomes

A study research staff or research staff/study nurse will call participants at follow-up on day 7 (+ 2 days), day 14 (+2 days), and 28 (+2 days) and at 3 months (+7 days) to assess: 1) the impact of the treatment and placebo on patients' QoL at 7 and 28 days, as well as 3 months after randomisation, 2) cough-related QoL captured with the LCQ sub-domains physical, psychological, and social 7, 14, and 28 days, and 3 months after randomisation, 3) the overall cessation of cough 7, 14, and 28 days, and within 3 months after randomisation, 4) the incidence rate of re-consultations at GP and/or hospitalisations within 3 months following randomisation, 5) the total AE within 3 months after randomisation, 6) SAE within 3 months after randomisation. The secondary outcomes related to QoL will be measured as described for the primary endpoint. All other secondary outcomes will be measured using a study developed non-validated survey.

9.2.3 Assessment of other outcomes of interest

No other outcomes are planned to be assessed.

9.2.4 Assessment of safety outcomes

9.2.4.1 Adverse events

Corticosteroid potential side effects and complications are systematically recorded from the time of randomization (inform consent obtained at baseline) until the last follow-up calls at 3 months from randomization. Participants are informed and asked to immediately contact the GP or the study team in case of any possible side-effects. In case participants cannot reach the GP, they should visit the nearest hospital. This information is provided verbally and in writing to patients (Appendices Studieninformation_OSPIC_V1.0 and OSPIC Medication Guide_V1.0). For safety reasons, the study team will also contact the corresponding GP to ensure patient follow-up is arranged as soon as possible. In case of persistent coughing at 3 months from randomization, the patient will be advised to visit the GP again for new assessment and necessary further investigations.

GPs and research staff/study nurses are instructed to document time of onset, duration, resolution, actions to be taken, assessment of intensity and relationship with study treatment. See section 10 (Safety) for AE definition and procedures.

9.2.4.2 Laboratory parameters

Not applicable.

9.2.4.3 Vital signs

At baseline (day 0, first presentation at GP) GPs will decide what diagnostics are necessary and, if applicable, will record for the purposes of the study the following: CRP test, white blood cell count, body temperature, blood pressure, pulse, O_2 saturation, previous or present X-Ray, previous or present lung function assessment. After the inclusion in the study it is at the discretion of the treating GP to re-assess each participant at the general practice, when and as often as clinically needed regarding physical examination, lab testing, performing X-rays (e.g. chest), or decide to hospitalize the patient if indicated.

9.2.5 Assessments in participants who prematurely stop the study

GPs will inform the patient on the importance to adhere to the 5-day medication intake in a manner consistent with usual care recommendations to follow the treatment as prescribed. The GPs will remind participants on the importance for the trial conduct to be available for the follow-up phone calls. Participants are encouraged to answer the follow-up questions. If patients prematurely stop the study or do not answer the follow-up call, the study team can contact the GP to ask about possible GP visits (AEs or SAEs) or hospitalizations (i.e. for pneumonia). Data will be collected until the time of withdrawal and will be analysed in the intention to treat analysis.

9.3 Procedures at each visit

9.3.1 Baseline Day 0, Visit 1

All patients presenting to the GP with a diagnosis of post-infectious cough following an URTI will be informed about the study, allowed sufficient time to ask questions and invited to participate. Patients who agree to participate are going to be screened for eligibility by taking their medical history, physical examination including vital signs, lab tests (if indicated and at the discretion of the GP). Informed consent will be obtained in writing from all participants. GPs will complete an individual CRF with participant's baseline socio-demographic information: age, sex, smoking behaviour, information on household smoking, symptoms, current treatment, doctor consultations, and results from diagnostic tests, when performed. Participants will also be asked to complete the standardized LCQ questionnaire on day 0. Participants will be instructed on how to complete the LCQ and that the GP can answer any questions they may have. Participants are also informed about the follow-up phone calls by the research staff/study nurse and that the next phone call appointment will be at day 7 of the trial. After completion of the LCQ the physician will enter all relevant patient information and contact data in the paper CRF and send the data to the CTU in order to facilitate access by the research staff/study nurse. Follow-up calls are carried by the CTU at the University Hospital Basel.

After completion of the LCQ the GP gives the trial medication to the patient.

9.3.2 Day 7 (+2 days), Phone Call 1

Patients will be called by study research staff/study nurses to complete the LCQ and to answer other questions regarding cough status, side effects, concomitant therapy, cessation of cough, re-consultations/hospitalisation, AE, SAE. If participants cannot be reached, the study nurse/research staff will send a reminder email and try calling back in the next 2 days from day 7.

9.3.3 Day 14 (+2 days), Phone Call 2

Patients will be called by study research staff/study nurses to complete the LCQ and to answer other questions regarding cough status, side effects, concomitant therapy, cessation of cough, re-consultations/hospitalisation, AE, SAE. If participants cannot be reached, the study nurse/research staff will send a reminder email and try calling back in the next 2 days.

9.3.4 Day 28 (+2 days), Phone Call 3

Patients will be called by study research staff/study nurses to complete the LCQ and to answer other questions regarding cough status, side effects, concomitant therapy, cessation of cough, re-consultations/hospitalisation, AE, SAE. If participants cannot be reached, the study nurse/research staff will send a reminder email and try calling back in the next 2 days.

9.3.5 Month 3 (+7 days), Phone Call 4

Patients will be called by the research staff/study nurses to complete the LCQ and to answer other questions regarding cough status, side effects, concomitant therapy, cessation of cough, re-consultations/hospitalisation, AE, SAE. If participants cannot be reached, the study nurse/research staff will send a reminder email and try calling back in the next 7 days.

10. SAFETY

10.1 Drug studies

During the entire duration of the study, adverse events (AEs) of special interest, as listed in the follow-up CRF, and all serious adverse events (SAEs) are collected, fully investigated and documented in source documents and case report forms (CRFs). Study duration encompasses the time from when the participant signs the informed consent (at randomization) until the last protocol-specific procedure at follow-up (month 3 from randomization) has been completed. However, we expect that the incidence of AEs or SAEs will be low, as the study uses an approved standard treatment for different conditions (asthma, COPD) which have a comparable pathogenesis to post-infectious cough. Please refer to section 10.1.2 for details on reporting.

10.1.1 Definition and assessment of (serious) adverse events and other safety related events

An **Adverse Event (AE)** is any untoward medical occurrence in a patient or a clinical investigation participant administered a pharmaceutical product and which does not necessarily have a causal relationship with the study procedure. An AE can therefore be any unfavourable and unintended sign (including an abnormal laboratory finding), symptom, or disease temporally associated with the use of a medicinal (investigational) product, whether or not related to the medicinal (investigational) product.

A **Serious Adverse Event (SAE)** is classified as any untoward medical occurrence that:

- results in death,
- is life-threatening,
- requires in-patient hospitalization or prolongation of existing hospitalisation,
- results in persistent or significant disability/incapacity, or
- is a congenital anomaly/birth defect.

In addition, important medical events that may not be immediately life-threatening or result in death, or require hospitalisation, but may jeopardise the patient or may require intervention to prevent one of the other outcomes listed above should also usually be considered serious.

Examples of such events are intensive treatment in an emergency room or at home for allergic bronchospasm, blood dyscrasias or convulsions that do not result in hospitalisation, or development of drug dependency or drug abuse.

SAEs should be followed until resolution or stabilisation. Participants with ongoing SAEs at study termination (including safety visit) will be further followed up until recovery or until stabilisation of the disease after termination.

Assessment of Causality

Prof. Dr. med. Andreas Zeller (Sponsor-Investigator) and Prof. Dr. med. Jörg Leuppi will review all SAEs and make a causality assessment of the event to the study drug, based on the criteria listed in the ICH E2A guidelines:

Relationship	Description
Definitely	Temporal relationship<br>Improvement after de-challenge*<br>Recurrence after re-challenge

	(or other proof of drug cause)
Probably	Temporal relationship<br>Improvement after de-challenge<br>No other cause evident
Possibly	Temporal relationship<br>Other cause possible
Unlikely	Any assessable reaction that does not fulfil the above conditions
Not related	Causal relationship can be ruled out
*Improvement after de-challenge only taken into consideration, if applicable to reaction	

Unexpected Adverse Drug Reaction

An "unexpected" adverse drug reaction is an adverse reaction, the nature or severity of which is not consistent with the applicable product information (e.g. Investigator's Brochure for drugs that are not yet approved and Product Information for approved drugs, respectively).

Suspected Unexpected Serious Adverse Reactions (SUSARs)

Prof. Dr. med. Andreas Zeller (Sponsor-Investigator) and Prof. Dr. med. Jörg Leuppi evaluate any SAE that has been reported regarding seriousness, causality and expectedness. If the event is related to the investigational product and is both serious and unexpected, it is classified as a SUSAR.

Note: In case of double-blinded studies, unblinding is needed in order to determine a SUSAR. Treatment allocation should not be disclosed to the investigator, nor to the study staff, in order not to make the subject ineligible.

Assessment of Severity

To assess any potential harms, the study team evaluates AE of interest and specifically SAE according to the WHO-UMC causality categories "certain", "probable", "possible", and "unlikely" (16).

The severity grading scale used for the study are described in the „Common Terminology Criteria for Adverse Events", CTCAE Version 5.0 (https://ctep.cancer.gov/protocolDevelopment/electronic_applications/ctc.htm#ctc_50) terminology is going to be used for adverse events occurring throughout this study: Grade 1 - Mild, Grade 2 - Moderate, Grade 3 - Severe, Grade 4 - Life-threatening, Grade 5 - Death.

10.1.2 Reporting of serious adverse events (SAE) and other safety related events

Reporting of SAEs

All participant SAEs captured during the follow-up interviews will be recorded by study nurses/staff who will report them within a maximum of 24 hours via the eCRF secuTrial© to Prof. Dr. med. Andreas Zeller, the Sponsor-Investigator for the study.

Participant SAEs that are reported to GPs or self-reported by participants by directly contacting the OSPIC study team in Liestal will be recorded and reported within a maximum of 24 hours via email or post to Prof. Dr. med. Andreas Zeller, the Sponsor-Investigator for the study.

Prof. Dr. med. Andreas Zeller will sign off, evaluate the SAE for causality and, if necessary, report them to the EKNZ.

SAEs resulting in death are reported to the ENKZ (via Prof. Dr. med. Andreas Zeller) via BASEC within 7 days.

Reporting of SUSARs

A SUSAR needs to be reported to EKNZ (via Prof. Dr. med. Andreas Zeller, Sponsor-Investigator) via BASEC and to Swissmedic for category B within 7 days, if the event is fatal, or within 15 days (all other events).

Prof. Dr. med. Andreas Zeller, the Sponsor-Investigator, must inform all Investigators participating in the clinical study of the occurrence of a SUSAR.

Reporting of Safety Signals

All suspected new risks and relevant new aspects of known adverse reactions that require safety-related measures, i.e. so-called safety signals, must be reported to Prof. Dr. med. Andreas Zeller, the Sponsor-Investigator, within 24 hours. Prof. Dr. med. Andreas Zeller must report the safety signals within 7 days to the EKNZ (local event via local Investigator) via BASEC and to Swissmedic in case of a category B study.

Prof. Dr. med. Andreas Zeller, the Sponsor-Investigator must immediately inform all participating Investigators about all safety signals.

Reporting and Handling of Pregnancies

The evidence to date regarding corticosteroid exposure in pregnancy and select pregnancy and birth outcomes is limited and inconsistent (41). The evidence to date suggests that first trimester corticosteroid use may confer a small increase in the odds of cleft lip in foetuses, with or without cleft palate. However, data are conflicting and it is unknown to what extent the underlying maternal disease may contribute. There is little support that systemic corticosteroid use in pregnancy independently causes increases in risks of preterm birth, low birth weight, or preeclampsia. There is not sufficient evidence to determine whether corticosteroids could contribute to gestational diabetes mellitus. This evidence is particularly true for long-term application of corticosteroid in pregnancy (>4 weeks) (41).

In the present study corticosteroids are given for 5 days (short-term). According to Embryotox, an internet-based register on pharmaco-vigilance and embryo-toxicology (University of Charité, Berlin, Germany) the 'Planning a therapy or planning a pregnancy during treatment' may also take place 'during pregnancy for a specific systemic or local treatment with prednisone. If therapeutically possible, the maintenance dose should not exceed 10 mg / day between week eight and eleven. Emergency treatment and therapy for acute disease exacerbation are not subject to any dose restrictions '. (German original text: «*Planung einer Therapie oder Planung einer Schwangerschaft unter Therapie*» *darf* «*eine indizierte systemische oder lokale Behandlung mit Prednison auch in der Schwangerschaft erfolgen. Wenn es therapeutisch möglich ist, sollte die Erhaltungsdosis zwischen Woche acht und elf möglichst 10 mg/Tag nicht überschreiten. Notfallbehandlungen und die Therapie einer akuten Krankheitsexazerbation unterliegen keinen Dosisbeschränkungen.*» (https://www.embryotox.de/arzneimittel/details/prednison/).

GPs will screen all female participants of reproductive age (postmenarche to menopause) using a battery of questions and will exclude all females who are pregnant, plan or are actively trying to get pregnant or women who are potentially pregnant. Women with anamnestic risk of pregnancy shall not be included in this study (unprotected sexual intercourse in the last two weeks). Pregnant participants must immediately be withdrawn from the clinical study. If a participant gets pregnant during follow-up, the course and the outcome of the pregnancy should be followed up carefully by GPs, documented and reported. Any pregnancy during the treatment phase of the study and within 30 days after discontinuation of study medication will be reported to Prof. Dr. med. Andreas Zeller (Sponsor-Investigator) within 24 hours. The course and outcome of the pregnancy should be followed up carefully, and any abnormal outcome regarding the mother or the child should be documented and reported.

Periodic reporting of safety

An annual safety report is submitted once a year via Prof. Dr. med Andreas Zeller (Sponsor-Investigator) to the local EKNZ and to Swissmedic in case of a category B.

The annual safety report contains information from all participating GP practices. The Sponsor-Investigator prepares it, and then submits it to the ethics committee.

10.1.3 Follow up of (Serious) Adverse Events

Every possible step will be taken to ensure that all AEs of interest or SAEs are identified and documented. GPs are asked to follow-up all patients with suspected AEs of interest or SAEs and to inform the study Sponsor, Prof. Dr. med. Andreas Zeller. Given the short duration of the treatment with the trial drug, it is expected that the incidence of AEs or SAEs is reasonably small. Please see more information in section 10.1 Drug studies.

11. STATISTICAL METHODS

11.1 Hypothesis

Null hypothesis:

A 5-day treatment with 40 mg (2 tablets of 20 mg) oral prednisone once daily for patients consulting a GP due to post-infectious cough after an URTI (3 to 8 weeks) will be superior to placebo and improve cough-related QoL after 14 days after randomisation (more than the minimally clinical important difference (MCID) of 1.3 of the Leicester QoL score).

Alternative hypothesis:

A 5-day treatment with 40 mg (2 tablets of 20 mg) oral prednisone once daily for patients consulting a GP due to post-infectious cough after an URTI (3 to 8 weeks) will significantly change cough-related QoL after 14 days after randomisation compared to patients who only got placebo.

11.2 Determination of Sample Size

Sample size was estimated to have 80% power to detect the minimal clinically important difference (MCID) set at 1.3 points (14).
To be able to detect an MCID of 1.3 points with a power of 80%, a total of N=204 patients need to be recruited for both arms. This was calculated without considering intra-patient correlation (IPC) correlation rho (ρ) between baseline and follow-up and without potential intra-cluster correlation (ICC) of patients treated by the same physician. To further increase power, we ignored ρ by calculating the sample size for a two-sample t-test with a two-sided alpha threshold of 5%. Sample size estimation was based on the assumption that individual LCQ scores are normally distributed. Chung et al. reported a standard deviation (SD) of 3.3 points (24). A recent trial with a design and study population similar to ours (15) reported a SD of 2.9.
We decided to use the more conservative assumption of 3.3 points. Due to the fact that the number of recruited patients per GP is limited to 10, ICC might remain small.

We expect a drop-out rate of 10%, similar to that in the abovementioned recent trial (15).

A less conservative choice of an SD of 2.9 and a ρ of 0.4, with the other parameters remaining the same, would have required a sample size of N=120. Hence, we assume that by the conservative choice of SD and by neglecting ρ, our calculation will sufficiently compensate for loss of power due to ICC and drop-outs. To compute the t-test, the current version of the *R* language and environment (www.r-project.org) function “power.t.test” of the stats package was applied.

11.3 Statistical criteria of termination of trial

None. No interim analyses are planned.

11.4 Planned Analyses

Detailed description of analyses will be defined in a statistical analysis plan (SAP) before unblinding the trial. The SAP will follow the ICH E9 Guideline Statistical Principles for Clinical Trials (33). Changes to the SAP will be under version control at the CTU, University Hospital Basel. The full study protocol will be published in a peer-reviewed journal under open access (e.g. Trials).

Data will be analyzed using R.

11.4.1 Datasets to be analysed, analysis populations

The full analysis set (FAS) will contain all patients who were randomly allocated to one of the trial arms.
The per-protocol-set (PPS) will include all patients with full i) adherence to the allocated 5-day treatments (took all doses as defined by the study protocol) and ii) complete primary outcome and LCQ (11) score at baseline. We will consider adjustments for time-varying post-randomization confounding (34).

11.4.2 Primary Analysis

Analysis of the primary objective will follow the intention-to-treat (ITT) principle. It will be based on the full analysis set (FAS). FAS will include all patients who gave informed consent. Patient data will be analysed according to their treatment allocation.

We will test if there is a difference of the LCQ score between the intervention and control group 14 days after randomization. A treatment effect of 1.3 points increase will be considered as MCID.
The hypothesis will be tested using analysis of covariance (ANCOVA). We will report the treatment effect with 95% confidence intervals (CI). Covariates of the ANCOVA model will be baseline LCQ scores, duration of cough, age, sex, and smoking status. Patients are considered as smokers, when they answer that they had smoked "more than 100 cigarettes in their life", they smoke "daily" or "sometimes" to the participant survey (29).
Baseline characteristics of patients in the FAS will be presented stratified by group and summarized in a table.
To assess the robustness of our primary analysis, the following sensitivity analyses will be performed:

- · Analysis of the primary outcome without imputing data (complete case analysis)
- · Analysis of the primary outcome using the per-protocol data set (PPS) which is defined above
- · Exploring interactions between covariates of the ANCOVA model of the primary analysis
- · Exploring how the effect of the intervention varies among GP practices. For this, a linear mixed-model with treatment group as a fixed-effect and GP practices as a random effect will be fit.

We will conduct one subgroup analysis, comparing effects on the primary outcome in current smokers vs. current non-smokers. Subgroup effects will be analyzed by interaction tests and interpreted fully exploratory. We expect that effects are more pronounced in non-smokers according to reports by Ponsioen et al. (20).

Statistical analysis will be performed by the CTU of the University Hospital Basel using R language and environment (www.r-project.org).

11.4.3 Secondary Analyses

The analysis of secondary outcomes will be of exploratory nature and results will be interpreted only for hypothesis generation. Summary statistics of cough-related QoL assessed by the LCQ score 7, 28 days, and 3 months after randomization, cough-related QoL sub-domains physical and psychological, and social 7, 14, 28 days, and 3 months after randomization will be presented in tables and figures. Total number and percentages will be calculated for overall cessation of cough 7, 14, 28 days, and 3 months after randomization, incidence rate of re-consultations at GP and/or hospitalizations within 3 months following randomization.

11.4.4 Interim analyses

No interim analysis will be performed.

11.4.5 Safety analysis

Total number and percentages will be calculated for incidence rate of re-consultations at GP and/or hospitalizations within 3 months following randomization, and total AE and SAE stratified by WHO-UMC causality categories (16) within 3 months after randomization.

11.4.6 Deviation(s) from the original statistical plan

Changes to the SAP will be justified and reported under version control at the CTU, University Hospital Basel. The full study protocol will be published in a peer-reviewed journal under open access (e.g. Trials).

11.5 Handling of missing data and drop-outs

We expect 5-10% dropouts. Based on a recent similar study, we assume that for patients who completed the study, only few data will be missing (17). The reason for missing data and whether it might be at-random or not, will be examined according to guidelines (EMA (European Medicines Agency) (18). If missing data is assumed to be "not at random", sensitivity analyses will be performed. If missing data is of type "missing completely at random" or "missing at random" data will be imputed using the method of multiple imputation by chained equations (MICE). Variables to be used in the imputation models will be chosen during assessment of kind and frequency of missing data. Multiple imputation will be performed using the Rpackage "mice" (35). The imputation procedure will be defined in the SAP.

12. QUALITY ASSURANCE AND CONTROL

12.1 Data handling and record keeping / archiving

Data acquisition will be performed by GPs, entering data in a paper case report form. Study data will be collected at the CTU and captured via a secuTrial© database based at the IT Department of the University Hospital Basel. The data collected with GPs (paper CRF) is entered into the study eCRF by trained study nurses of the CTU. Study data entered into the eCRF are only accessible by authorized persons. An integrated audit trail will maintain a record of initial entries and any changes made; time and date of entry; and user name of person authorizing entry or change.

Data about adverse events will be reported by GPs to the research team at the study centre in Liestal who will then review the adverse events and report to local authorities, if necessary. The CTU at the University Hospital Basel will monitor and prepare the data for export and transfer to the Sponsor for archiving. The data will be archived for 10 years by Prof. Dr. med. Andreas Zeller.

12.1.1 Case Report Forms

Paper CRF will be delivered to GPs. Data at baseline will be recorded with paper CRF at the GP's practice. All paper CRFs will be collected at the CTU and manually entered into an electronic CRF and then control and, if needed, correction of content is performed (eCRF). Data from follow-up will be added into the participant eCRF. For each enrolled study participant, an individual eCRF is maintained. Data entry will be performed by trained research staff/study nurses. eCRFs will be kept current to reflect subject's status at each phase during the course of study. The principal investigator will be responsible for assuring that the data entered into the eCRF is complete, accurate, and that the entry and updates are performed in a timely manner. The eCRF will be implemented by the Data management group at the CTU of the University Hospital Basel. Participants will not be identified in the eCRF by name or initials and birth date. Appropriate coded identification (patient ID) is used. The research staff/study nurses will be authorized to do eCRF entries. Source data to document the existence of the patients will be available at GP practices participating in the study.

12.1.2 Specification of source documents

Source data will include the original documents in the participant files: eligibility screen, the Informed Consent Form, the worksheet/paper CRF which includes socio-demographic and medical information at baseline, study medication dispensing, randomisation number, LCQ questionnaire and concomitant medication, and worksheets recording follow-up data (LCQ, medication intake, other medication, symptoms, adverse events, etc.). The signed informed consent and the participant lists will be stored in paper form with the GP until the end of the study. The worksheets/paper CRFs and LCQ questionnaires completed at baseline will be sent to the CTU for data entry into the eCRF.

12.1.3 Record keeping / archiving

All study data must be archived for a minimum of 10 years after study termination or premature termination of the clinical trial. The storage location will be at the study center in Liestal (uniham-bb). The Clinical Data Management application (CDMA) will be locked after eCRF data entry is completed, all data has been monitored and raised queries have been resolved. The complete study dataset will be exported from the database and transferred to the study statistician as well as the principal investigator according to the DMP. The exported data will be archived for 10 years by the principal investigator Prof. Dr. med Andreas Zeller on a hard drive, in a password protected folder and stored in a locked cabinet at the Centre for Primary Health Care (uniham-bb).

12.2 Data management

GPs will complete the paper CRF which will then be read-out and imported in an eCRF at the CTU. Data will be controlled and a correction of the content of the read-out will be carried to ensure that correctly filled paper CRF is read completely and accurately. In case the paper CRF raises queries, these will be resolved through inquiries with individual GPs. Read-out CRF data will be formatted and merged with phone interview data in the eCRF. Study data entered into the eCRF are only accessible by authorized persons.

An integrated audit trail will maintain a record of initial entries and any changes made; time and date of entry; and user name of person authorizing entry or change. For each patient enrolled an eCRF must be completed. The principal investigator will be responsible for assuring that the data entered into the eCRF is complete, accurate, and that the entry and updates are performed in a timely manner. The eCRF will be implemented by the Data management group at the CTU of the University Hospital Basel. Data entry will be performed by trained research staff/study nurses.

Password protection and user right management ensures that only authorised study personal, data managers and local authorities (if necessary) will have access to the data during and after the study.

Direct access to source documents will be permitted for purposes of monitoring, audits and inspections. Monitoring will be carried by the CTU at the University Hospital Basel, as described in Section 12.3 Monitoring.

The CTU at the University Hospital Basel will monitor and prepare the data for export and transfer to the Sponsor for archiving.

Metadata describing the type, size and content of the datasets will be shared along with the study protocol and eCRF on the public repository *dataverse.harvard.edu*. Additionally, the eCRF templates will be uploaded on *medical-data-models.org* and all variables will be annotated by their Unified Medical Language System Concept Unique Identifier (UMLS CUI) to improve findability for other clinicians. Researchers who wish to reuse data may submit a project synopsis at *dfk.unibas.ch/contact*. It is the responsibility of those researchers to seek a new approval for their study from the ethics committee.

12.2.1 Data Management System

Study data will be read-out and entered into the study eCRF. The eCRF is implemented by the Data management group at the CTU, University Hospital Basel. The secuTrial® database runs on a server maintained by the IT-Department of the University Hospital Basel. Additional storage capacity can be added as needed.

12.2.2 Data security, access and back-up

Study data entered into the eCRF are only accessible by authorized persons. Password protection and user right management ensures that only authorised study personal, data managers and local authorities (if necessary) will have access to the data during and after the study. User administration and user training is performed by the CTU Basel according to predefined processes., according to the Standard Operating procedures of the DKF.. Back-up of secuTrial® study data is performed regularly according to the processes of the IT-Department of the University Hospital Basel.

12.2.3 Analysis and archiving

The CDMA will be locked after eCRF data entry is completed, all data has been monitored and raised queries have been resolved. The complete study dataset will be exported from the database and transferred to the study statistician as well as the principal investigator through a secure channel without breaking the blinding. The statistical analysis will be performed completely independent by the involved statistician at the CTU of the University Hospital Basel. The exported data will be archived for 10 years by the principal investigator. The CTU will store long-term the CDMA.

12.2.4 Electronic and central data validation

The data managers of the CTU Basel will implement validation rules in the secuTrial®. When data gets saved in an eCRF, the read-out content will be controlled and corrected, if needed. IF paper CRF is incorrectly filled, inquiries will be made with individual GPS. Data will be validated for completeness and discrepancies. An integrated audit trail will maintain a record of initial entries and any changes made; time and date of entry, and user name of person authorizing entry or change.

12.3 Monitoring

Data monitoring will be performed by the CTU of the University Hospital Basel. Monitoring will be carried out according to the SOP's of the CTU and on the basis of the monitoring plan, agreed upon with the Sponsor. One routine monitoring visit (RMV) will be performed in 8 randomly assigned centres after inclusion of 1 to 3 participants at those sites. In 8 centres with the highest number of randomized participants, a second RMV will be scheduled after 'last patient/ last visit' at each site. A follow-up report

will be written by the CTU staff.

Direct access to source documents will be permitted for purposes of monitoring, audits and inspections. The local investigators ensure that source data and documents are made accessible to the study monitor and answer questions by the study monitor. Detailed description of monitoring activities will be defined in the study specific monitoring plan.

The CTU staff will also provide support for site initiation visits. Please see Appendix CTU Offer_October 2019 for more information.

12.4 Audits and Inspections

Regulatory authorities can audit this trial independently from the investigator/sponsor. Study documentation and data are accessible to auditors/inspectors and questions are answered during inspections. All involved parties must keep the participant data strictly confidential.

12.5 Confidentiality, Data Protection

During the study, confidentiality will be guaranteed. The principal investigator will guarantee the study's compliance with national and international data security. All data will be coded by the GPs and data will be stored and analysed in a coded way. Password protection and user right management is used for the eCRF and ensures that only authorised study personal, data managers and local authorities (if necessary) will have access to the data during and after the study. Participant contact information will be collected for carrying follow-up calls and will be filled in the paper CRF form by the GP. Only research staff conducting the follow-up interviews will have access to the participants' contact data. Participant lists will be kept at the GP practices for the entire duration of the study. After the end of the study, the lists will be sent to the PI and included in the Investigator Site File (ISF). The ISF will be archived for 10 years according to ICH-GCP. The study team at the CTU will maintain a separate participant/contact list, which will be included into the ISF at the end of the study.

Third persons will not gain any insight into source data. All involved parties must keep the participant data strictly confidential. The study protocol and dataset will be accessible to any regulatory authority after publication for at least 10 years. For more information please refer to section 13. Publication and Dissemination Policy.

12.6 Storage of biological material and related health data

Not applicable.

13. PUBLICATION AND DISSEMINATION POLICY

The Department of Clinical Research of the University Hospital Basel (DKF) will act as an independent Data Access Committee (DAC) and store the CDMA at time of publication on secure servers, maintained and backed up by the IT-Department of the University Hospital Basel. Researchers who wish to reuse data may submit a project synopsis at dkf.unibas.ch/contact. Metadata describing the type, size and content of the datasets will be shared along with the study protocol and eCRF on the public repository dataverse.harvard.edu. Additionally, the eCRF templates will be uploaded on medical-data-models.org and all variables will be annotated by their Unified Medical Language System Concept Unique Identifier (UMLS CUI) to improve findability for other clinicians. The results of this study will be published in a peer-reviewed medical journal, independent of the results. All results from this study will be published in an anonymized way. We will consider all relevant reporting guidelines of the EQUATOR-network for publication of the study protocol and any results publications (36-40). We will also update the study team's published systematic review (6) using the data of the OSPIC study as soon as possible.

The OSPIC trial will be registered on the international trial register www.clinicaltrials.gov and the national register www.kofam.ch where we will provide a publicly available synopsis of the study protocol. A data sharing statement referring researchers to the DKF for data access will be contained in the study protocol and publication. The study protocol will be published in a peer-reviewed journal. The entries in the trial registry will be kept up-to-date and completed with study results after completion of the trial.

14. FUNDING AND SUPPORT

14.1 Funding

The OSPIC study is fully funded by the SNSF (Swiss National Science Foundation) through an Investigator Initiated Clinical Trials grant (IICT 2018 call, 33IC30_179657 3) for a duration of 3 years, starting on 1 June 2019.

14.2 Other Support

The OSPIC study is supported by the research groups of the Centre for Primary Health Care, University of Basel (uniham-bb) and of the Institute of Primary and Community Care, Lucerne. The study is also supported by Prof. Dr. med. Jörg Leuppi, Kantonsspital Baselland, Liestal. The Centre for Primary Health Care has its offices in the Kantonsspital Baselland and will act as the coordinating centre for the conduct of the study. The Centre (uniam-bb) has office and infrastructure for a total of 5 researchers as well as storage facilities according to Swissmedic requirements. Storage facilities can be accessed and used throughout the trial. All collaborating investigators mentioned above support this project and make substantial non-financial contributions.

The CTU at the University Hospital Basel is supporting the study and provides monitoring, data management and statistical services, in accordance with the electronic agreement from 25 October, 2019 (Appendix CTU Offer October 2019). The study is also supported by the Pharmacy department of the University Hospital Basel, based on the electronic offer from 30 September, 2019 (Appendix CTU Offer September 2019).

15. INSURANCE

Insurance will be provided by the Sponsor. The study is insured by Helvetia Schweizerische Versicherungsgesellschaft AG, Dufourstrasse 40, 9001 St. Gallen.

A copy of the certificate shall be filed in each investigator site file and the trial master file.

16. REFERENCES

1. Rosendal M, Carlsen AH, Rask MT, Moth G. Symptoms as the main problem in primary care: A cross-sectional study of frequency and characteristics. Scand J Prim Health Care. 2015;33(2):91-9.

2. Kardos P, Berck H, Fuchs KH, Gillissen A, Klimek L, Morr H, et al. Guidelines of the German Respiratory Society for diagnosis and treatment of adults suffering from acute or chronic cough. Pneumologie. 2010;64(11):701-11.

3. French CT, Fletcher KE, Irwin RS. A comparison of gender differences in health-related quality of life in acute and chronic coughers. Chest. 2005;127(6):1991-8.

4. Braman SS. Postinfectious cough: ACCP evidence-based clinical practice guidelines. Chest. 2006;129(1 Suppl):138-46.

5. El-Gohary M, Hay AD, Coventry P, Moore M, Stuart B, Little P. Corticosteroids for acute and subacute cough following respiratory tract infection: a systematic review. Fam Pract. 2013;30(5):492-500.

6. Speich B, Thomer A, Aghlmandi S, Ewald H, Zeller A, Hemkens LG. (2018). Treatments for subacute cough in primary care: systematic review and meta-analyses of randomized clinical trials. Br J Gen Pract, 68(675), e694-e702.

7. Bardin PG, Fraenkel DJ, Sanderson G, Lampe F, Holgate ST. Lower airways inflammatory response during rhinovirus colds. Int Arch Allergy Immunol. 1995;107(1-3):127-9.

8. Trigg CJ, Nicholson KG, Wang JH, Ireland DC, Jordan S, Duddle JM, et al. Bronchial inflammation and the common cold: a comparison of atopic and non-atopic individuals. Clin Exp Allergy. 1996;26(6):665-76.

9. Leuppi JD, Schuetz P, Bingisser R, Bodmer M, Briel M, Drescher T, Duerring U, Henzen C, Leibbrandt Y, Maier S, Miedinger D, Muller B, Scherr A, Schindler C, Stoeckli R, Viatte S, von Garnier C, Tamm M, Rutishauser J. Short-term vs conventional glucocorticoid therapy in acute exacerbations of chronic obstructive pulmonary disease: the REDUCE randomized clinical trial. JAMA. 2013;309(21):2223-31.

10. Ward N. The Leicester Cough Questionnaire. Journal of physiotherapy, 2016;62(1):53.

11. Birring SS, Prudon B, Carr AJ, Singh SJ, Morgan MDL, Pavord ID. Development of a symptom specific health status measure for patients with chronic cough: Leicester Cough Questionnaire (LCQ). Thorax, 2003;58(4):339-43.

12. Berkhof FF, Boom LN., ten Hertog, NE., Uil SM., Kerstjens HA., & van den Berg JW. The validity and precision of the Leicester Cough Questionnaire in COPD patients with chronic cough. Health and quality of life outcomes, 2012;10(1):4.

13. Schupp JC, Fichtner UA, Frye BC, Heyduck-Weides K, Birring SS, Windisch W, Criée C-P, Müller-Quernheim J, Farin E. Psychometric properties of the German version of the Leicester Cough Questionnaire in sarcoidosis. PLoS ONE, 2018;13(10):e0205308. https://doi.org/10.1371/journal.pone.0205308

14. Chung KF, Widdicombe JG. Pharmacology and therapeutics of cough. Springer;2009, pp. 311.

15. Wang et al. Montekulast for postinfectious cough in adults: a double-blind randomized placebo-controlled trial. Lancet Respir Med 2014;2:35–43.

16. WHO-UMC (World Health Organization Uppsala Monitoring Centre) 2017; https://www.whoumc.org, accessed on 19.10.2017.

17. Hay A; Little P, Harnden A, Thompson M, Wang K, Kendrick D, Orton E, Brookes ST, Young GJ, May M, Hollinghurst S, Carroll FE, Downing H, Timmins D, Lafond N, El-Gohary M, Moore M. JAMA. 2017 Aug 22;318(8):721-730. doi: 10.1001/jama.2017.10572.

18. European Medicines Agency. EMA. Guideline on Missing Data in Confirmatory Clinical Trials; EMA/CMP/EWP/1776/99 Rev.1; 2010.

19. Wilkinson MD, Dumontier M, Aalbersberg IJ, Appleton G, Axton M, Baak A, Blomberg N, Boiten JW, da Silva Santos LB, Bourne PE, Bouwman J, Brookes AJ, Clark T, Crosas M, Dillo I, Dumon O, Edmunds S, Evelo CT, Finkers R, Gonzalez-Beltran A, Gray AJ, Groth P, Goble C, Grethe JS, Heringa J, 't Hoen PA, Hooft R, Kuhn T, Kok R, Kok J, Lusher SJ, Martone ME, Mons A, Packer AL, Persson B, Rocca-Serra P, Roos M, van Schaik R, Sansone SA, Schultes E, Sengstag T, Slater T, Strawn G, Swertz MA, Thompson M, van der Lei J, van Mulligen E, Velterop J, Waagmeester A, Wittenburg P, Wolstencroft K, Zhao J, Mons B. The FAIR Guiding Principles for scientific data management and stewardship. Sci Data. 2016 Mar 15;3:160018. doi:

10.1038/sdata.2016.18. Erratum in: Sci Data. 2019 Mar 19;6(1):6. PMID: 26978244; PMCID: PMC4792175.

20. Ponsioen BP, Hop WC, Vermue NA, Dekhuijzen PN, Bohnen AM. Efficacy of fluticasone on cough: a randomised controlled trial. Eur Respir J. 2005;25(1):147-152.

21. Pornsuriyasak P, Charoenpan P, Vongvivat K, Thakkinstian A. Inhaled corticosteroid for persistent cough following upper respiratory tract infection. Respirology. 2005;10(4):520-4.

22. Johnstone KJ, Chang AB, Fong KM, Bowman RV, Yang IA. Inhaled corticosteroids for subacute and chronic cough in adults. Cochrane Database Syst Rev. 2013(3):CD009305.

23. Hayward GN, Hay AD, Moore MV, Jawad S, Williams N, Voysey M, Cook J, Allen J, Thompson M, Little P, Perera R. Effect of oral dexamethasone without immediate antibiotics vs placebo on acute sore throat in adults: a randomized clinical trial. Jama, 2017;317(15), 1535-1543.

24. O'Grady KF, Grimwood k, Torzillo PJ, Rablin S, Lovie-Toon Y, Kaus M, Arnold D, Roberts J, Buntain H, Adsett D, King A, Scott M, Anderson J, Toombs M, Chang AB. Effectiveness of a chronic cough management algorithm at the transitional stage from acute to chronic cough in children: a multicenter, nested, single-blind, randomised controlled trial. The Lancet Child & Adolescent Health. 2019; doi.org/10.1016/S2352-4642(19)30327-X.

25. Swiss compendium. Product information, PREDNISON Galepharm Tabl 20 mg. https://compendium.ch/product/618-prednison-galepharm-tabl-20-mg.

26. McAllister WA, Winfield CR, Collins JV. Pharmacokinetics of prednisone in normal and asthmatic subjects in relation to dose. Eur J Clin Pharmacol.1981;20:141-5.

27. Morice AH, McGarvey L, Pavord I. Recommendations for the management of cough in adults. Thorax 2006;61(Suppl I): i1–i24. doi: 10.1136/thx.2006.065144.

28. Walijee AK. Short term use of oral corticosteroids and related harms among adults in the United States: population-based cohort study. BMJ 2017;357: j1415 http://dx.doi.org/10.1136/bmj.j1415.

29. Ryan H, Trosclair A, Gfroerer J. Adult Current Smoking: Differences in Definitions and Prevalence Estimates. J Environ Public Health 2012;2012:918368.

30. Strassle T, Essig S, Merlo C, Zeller A. Post-infectious cough - How to treat? Oral presentation at the congress of the Swiss Society of General Medicine, Lausanne, 2017.

31. Spector SL. The common cold: current therapy and natural history. Journal of allergy and clinical immunology, 1995;95(5):1133-38.

32. Megroz R. Was kostet eine Stunde Arzt? Wirtschaftliche und andere Uberlegungen. Schweiz Arzteztg. 2004;85(24):1286-90.

33. ICH E9 1998 Guideline Statistical Principles for Clinical Trials, Step 4 Version. 30. Ryan H, Trosclair A, Gfroerer J. Adult Current Smoking: Differences in Definitions and Prevalence Estimates. J Environ Public Health 2012;2012:918368.

34. Hernan MA, Robins JM. Per-Protocol Analyses of Pragmatic Trials. N Engl J Med. 2017;377 (14):1391-8.

35. Buuren S, & Groothuis-Oudshoorn K. Mice: Multivariate imputation by chained equations in R. Journal of statistical software, 2011;45(3).

36. Chan A-W, Tetzlaff JM, Altman DG, Laupacis A, Gotzsche PC, Krleža-Jerić K, Hrobjartsson A, Mann H, Dickersin K, Berlin J, Dore C, Parulekar W, Summerskill W, Groves T, Schulz K, Sox H, Rockhold FW, Rennie D, Moher D. SPIRIT 2013 Statement: Defining standard protocol items for clinical trials. Ann Intern Med 2013;158:200-7.

37. Chan A-W, Tetzlaff JM, Gotzsche PC, Altman DG, Mann H, Berlin J, Dickersin K, Hrobjartsson A, Schulz KF, Parulekar WR, Krleža-Jerić K, Laupacis A, Moher D. SPIRIT 2013 Explanation and Elaboration: Guidance for protocols of clinical trials. BMJ 2013;346:e7586.

38. Hoffmann T, Glasziou P, Boutron I, Milne R, Perera R, Moher D, Altman DG., Barbour V, Macdonald H, Johnston M, Lamb S, Dixon-Woods M, McCulloch P, Wyatt J, Chan Phelan A-W, and Michie S. Better reporting of interventions: Template for intervention description and replication (TIDieR) checklist and guide" BMJ, 2014;348:g1687.

39. Schulz, K. F., Altman, D. G., & Moher, D. CONSORT 2010 statement: updated guidelines for reporting parallel group randomised trials. BMC medicine, 2010;8(1):18.

40. International Conference on Harmonisation Working Group. "ICH harmonised tripartite guideline: guideline for good clinical practice E6 (R1)." International Conference on Harmonisation of Technical Requirements for Registration of Pharmaceuticals for Human Use. Vol. 10. 1996.

41. Bandoli G, Palmsten K, Forbes Smith CJ, Chambers CD. A review of systemic corticosteroid use in pregnancy and the risk of select pregnancy and birth outcomes. Rheum Dis Clin North Am. 2017;43(3):489-502. doi: 10.1016/j.rdc.2017.04.013

17. APPENDICES

1. IMP: PREDNISON Galepharm Swissmedic_29Oct2019
   PREDNISON Galepharm Compendium_29Oct2019

2. List of study sites /GPs

3. Other

- Case Report Form, OSPIC Fragebogen für den Hausarzt (CRF)_final V2.0
- Case Report Form Follow-up (CRF) V1.0_19Dec2019
- OSPIC Fragebogen (CRF)_DataManagement V2.0
- Patient Information and informed consent, Studieninformation_OSPIC V2.0
- Other material to patients, OSPIC_Leaflet V1.1
- Other material to patients, OSPIC_Poster V1.1
- Other material to patients, OSPIC Medication Guide V2.0
- OSPIC_GCP Drehbuch Instruktionsvideo für Hausärzte_11.10.18_v1.5_final
- Clinical Study Agreement with GPs, OSPIC_Clinical_Study_Agreement_Hausarzt_V1.0
- Clinical Study Agreement Project leader, OSPIC_Agreement_PI_local project leader_V1.0
- Agreement with CTU, CTU Offer Contract 25 Oct 2019
- Agreement with the Pharmacy Department at University Hospital Basel, Pharmacy Offer 30 Sep 2019
- SNSF Funding Grant
- Insurance documentation
